# Supplementary material for: Efficient iron single-atom catalysts for selective ammoxidation of alcohols to nitriles
Source: Nat Commun. 2022 Apr 6;13:1848. doi: 10.1038/s41467-022-29074-1 (PMC8986860; doi:10.1038/s41467-022-29074-1)
Supplement: Supplementary file 1 — Supplementary Information [file 41467_2022_29074_MOESM1_ESM.pdf]

Efficient iron single-atom catalysts for selective ammoxidation of alcohols to nitriles

Kangkang Sun[1,2], Hongbin Shan[1], Helfried Neumann[2,*], Guo-Ping Lu[1,*], and Matthias Beller[2,*]

[1] School of Chemistry and Chemical Engineering, Nanjing University of Science & Technology, Xiaolingwei 200, Nanjing 210094 (P. R. China).

[2] Leibniz-Institute for Catalysis, Albert-Einstein-Str. 29a, 18059 Rostock, Germany.

* Correspondence to: Matthias.Beller@catalysis.de, glu@njust.edu.cn, and Helfried.Neumann@catalysis.de

Supplementary Tables

Supplementary Table 1. Comparison of performance of different catalysts reported in literature on the synthesis of aryl nitriles.

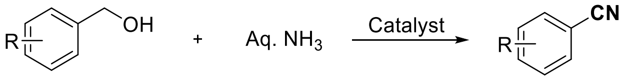

Entry	Catalyst		Oxidant	T(°C)	Solvent	Yield(%)	Ref.
1	$Ru(OH)_x/Al_2O_3$	10 mol% Ru	Air/6atm	120	THF	72	1
2	Pt/GO	2.5 mol% Pt	O_2/3atm	90	CH_3CN	96	2
3	Ag/N-CS-1	3 mol% Ag	O_2/1atm	80	EtOH	94	3
4	Ru/MnO_2-r	0.025-0.099 mol% Ru	O_2/5atm	100	*t*-amyl alcohol	>99	4
5	Co_3O_4-NGr/C or Fe_2O_3-NGr/C	4 mol% Co/4.5 mol% Fe	O_2/5atm	130	*t*-amyl alcohol	>99	5
6	Meso-Co-N/C(800)	0.5 mol% Co	O_2/5atm	130	*t*-amyl alcohol	95	6
7	meso-N/C-900	0.5 mmol/50mg	O_2/5atm	130	*t*-amyl alcohol	90	7
8	Co@NC	4 mol% Co	O_2/1atm	50	H_2O	93	8
9	NCI-Fe/Cu	0.67 mol% Fe/1.33 mol% Cu	O_2/5atm	130	*t*-amyl alcohol	>99	9
**10**	**Fe_1-N-C**	3.9 mol% Fe	**Air/1atm**	**35**	**H_2O**	**>99**	**This work**

Supplementary Table 2. Comparison of performance of different catalysts reported in literature on the synthesis of aliphatic nitriles.

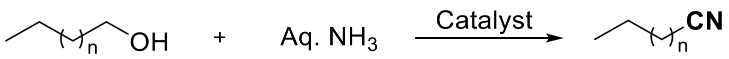

Entry	Substrate	Catalyst	Oxidant	T(°C)	Solvent	Yield(%)	Ref.
1	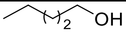	Ag/N-CS-1 3 mol%	O_2/0.1MPa	80	EtOH	0	3
2	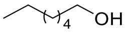	Ru/MnO_2-r 0.099 mol%	O_2/0.5MPa	130	*t*-amyl alcohol	37.8	4
3	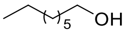	Fe_2O_3-NGr/C 6 mol%	O_2/0.5MPa	140	Water:n-heptane	60	5

4	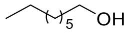	Meso-Co-N/C(800) 1 mol%	O_2/0.5MPa	140	Water:n-heptane	12	6
5	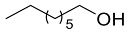	meso-N/C-900 70mg	O_2/0.5MPa	130	*t*-amyl alcohol	0	7
6	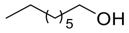	Co@NC 4 mol%	O_2/0.1MPa	50	H_2O	0	8
7	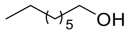	Fe_1-N-C 3.9 mol%	air/2MPa	130	H_2O	61	This work
8	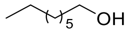	Fe_2O_3-NGr/C 3.9 mol%	air/2MPa	130	H_2O	23	comparison
9	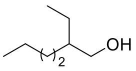	Fe_1-N-C 3.9 mol%	air/2MPa	130	H_2O	95	This work
10	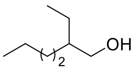	Fe_2O_3-NGr/C 3.9 mol%	air/2MPa	130	H_2O	26	comparison
11	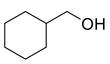	Fe_1-N-C 3.9 mol%	air/2MPa	130	H_2O	82	This work
12	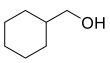	Fe_2O_3-NGr/C 3.9 mol%	air/2MPa	130	H_2O	7	comparison

Supplementary Table 3. Catalyst evaluation for the ammoxidation of benzyl alcohol[a].

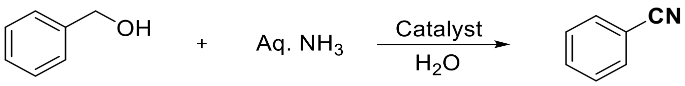

1a **2a** **3a**

Entry	Catalyst	Time (h)	Yield(%)
1	Fe_1-N-C	12	66
2	Fe_1-N-C	24	>99
3	Fe-N-C1	12	48
4	Fe-N-C2	12	48
5	Fe-N-C3	12	54
6[c]	Fe-N-C4	12	42
7[d]	Fe-N-C5	12	35

[a] Reaction conditions: 1a (0.2 mmol), 3.9 mol% Fe, aq. $NH_3.H_2O$ (150 mg, 25-28 wt%), 35°C, 0.1 MPa air, 1.5 mL H_2O; [b] Conversion and yield were determined by GC analysis using hexadecane as an internal standard; [c] The Fe-N-C4 material was obtained from the carbonation of Fe-ZIF-TEA; [d] The Fe-N-C5 material was obtained from the carbonation of Fe-ZIF-NaOH.

Supplementary Table 4. K-edge EXAFS fitting parameters of Fe_1-N-C.

Sample	Path	CN	R(Å)	$\sigma^2(10^{-3}Å^2)$	ΔE_0 (eV)	R-factor
Fe_1-N-C	Fe-N	4.3±0.1	1.97±0.01	9.5±0.5	-3.6±1.1	0.014

Supplementary Table 5. Elemental analysis of Fe_1-N-C and Fe/NC.

Entry	N (%)	C (%)	H (%)
Fe_1-N-C	4.63	67.15	2.217
Fe/NC	3.96	75.05	1.993

Supplementary Table 6. ICP-OES analysis of Fe_1-N-C.

Materials	Element	Metal Content (mg/kg)

Fe_1-N-C	Fe	21915
Fe_1-N-C[a]	Fe	21899

[a] Fe_1-N-C is the catalyst after six cycles. Digestion procedure: Approximately 10 mg of the sample was mixed with 8 ml of aqua regia and 2 ml hydrofluoric acid. The digestion was performed in a microwave-assisted sample preparation system at ~ 220°C and ~ 50 bar pressure. The digested solution was filled up to 100 ml and measured with ICP-OES.

Supplementary Figures

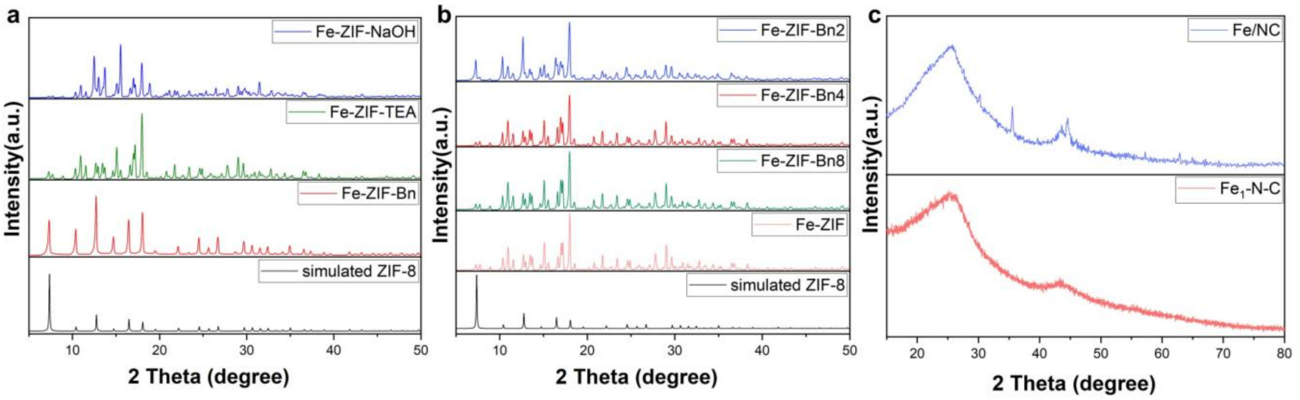

Supplementary Figure 1 XRD patterns. a simulated ZIF-8, Fe-ZIF-Bn, Fe-ZIF-TEA, and Fe-ZIF-NaOH; **b** simulated ZIF-8, Fe-ZIF, Fe-ZIF-Bn8, Fe-ZIF-Bn4, and Fe-ZIF-Bn2; **c** Fe_1-N-C and Fe/NC.

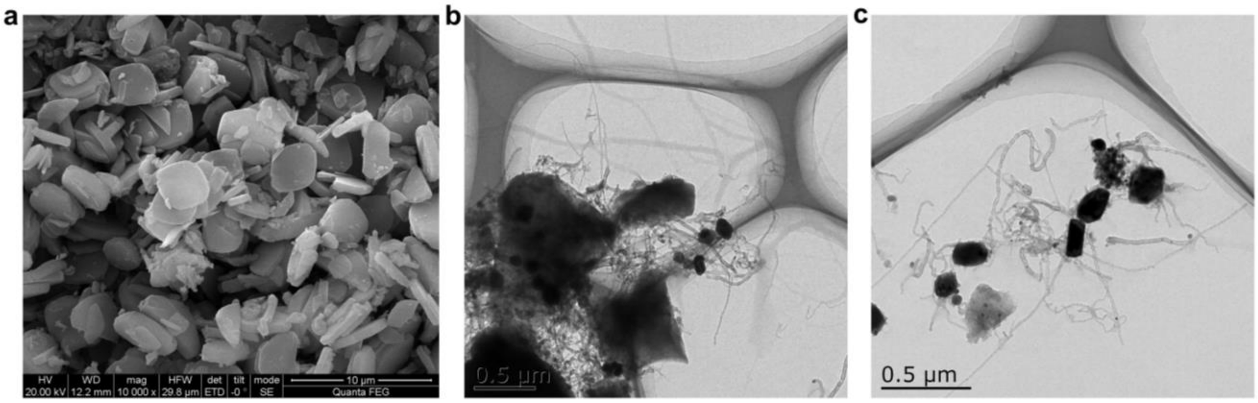

Supplementary Figure 2 Representative electron microscopy images. a SEM image of Fe-ZIF and **b**, **c** TEM images of Fe/NC.

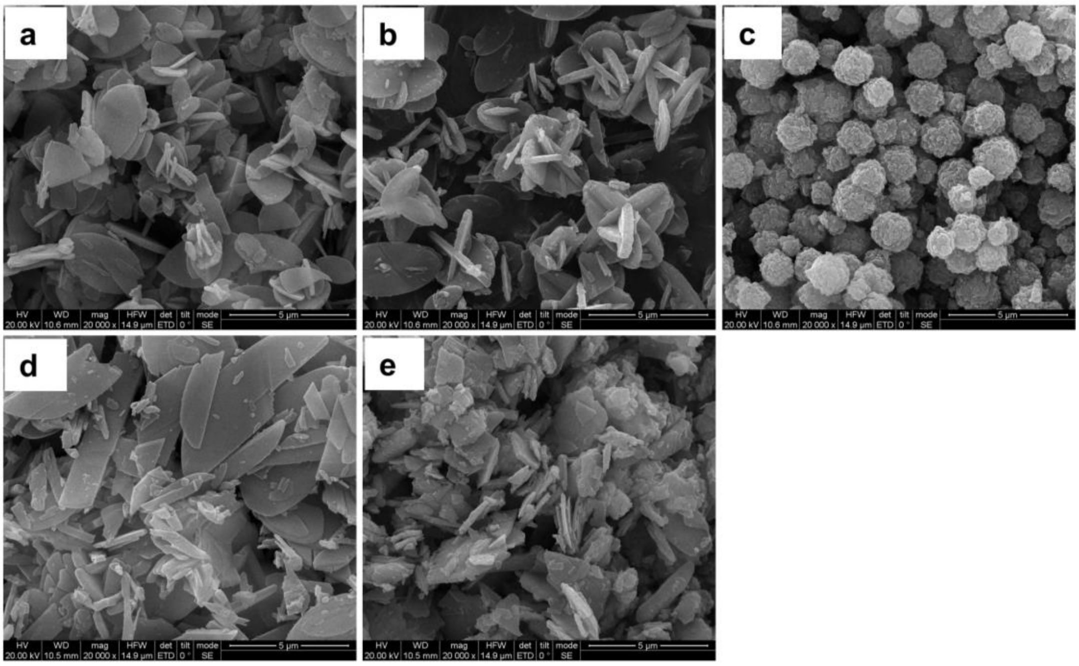

Supplementary Figure 3 SEM images. **a** Fe-ZIF-Bn8, **b** Fe-ZIF-Bn4, **c** Fe-ZIF-Bn2, **d** Fe-ZIF-TEA, and **e** Fe-ZIF-NaOH.

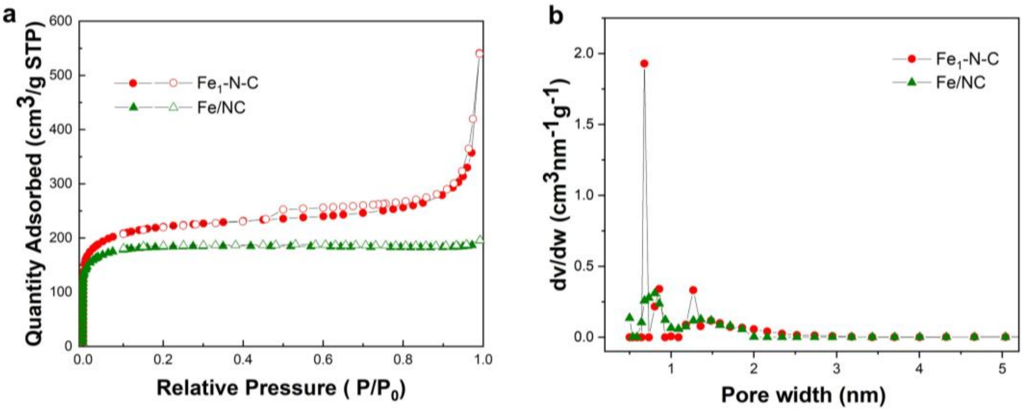

Supplementary Figure 4 BET surface area and pore size analysis. **a** Nitrogen adsorption–desorption isotherms and **b** the pore size distributions of Fe_1-N-C and Fe/NC.

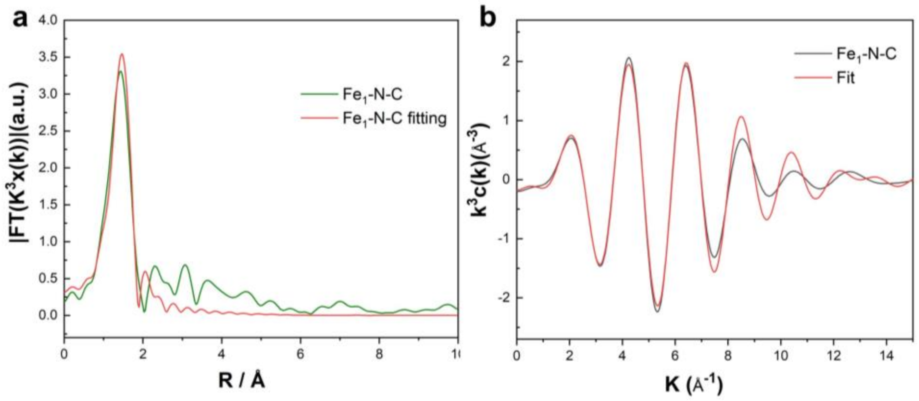

Supplementary Figure 5 The EXAFS spectra and corresponding fitting curves of Fe_1-N-C. **a** R space, **b** K space.

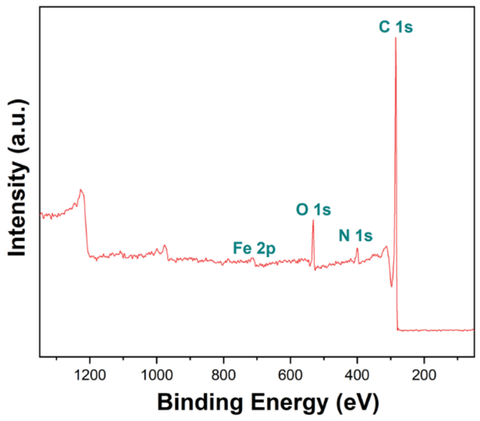

Supplementary Figure 6 XPS spectra. XPS survey spectrum of the sample Fe_1-N-C.

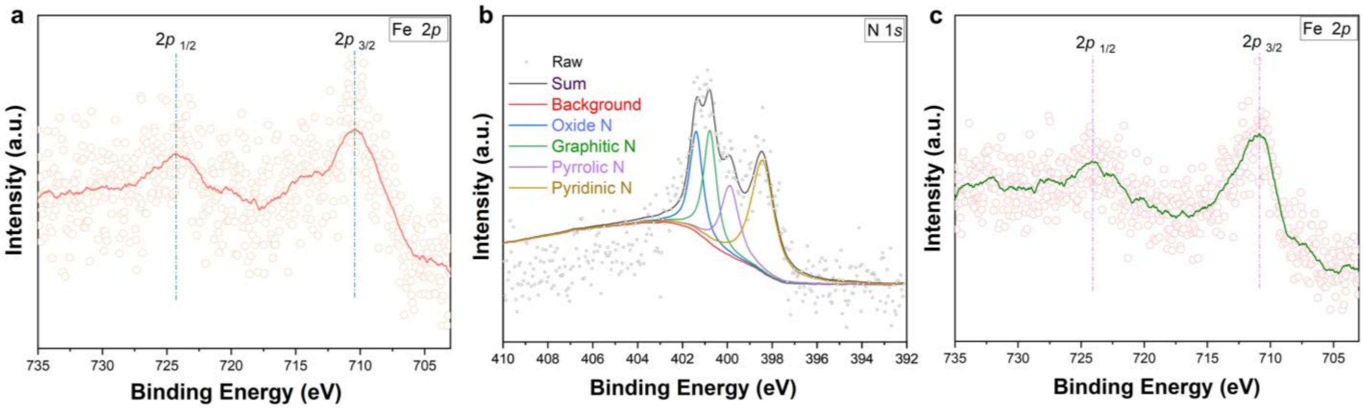

Supplementary Figure 7 XPS spectras. a Fe 2*p* and **b** N 1*s* XPS spectra of Fe_1-N-C; **c** Fe 2*p* XPS spectra of Fe/NC.

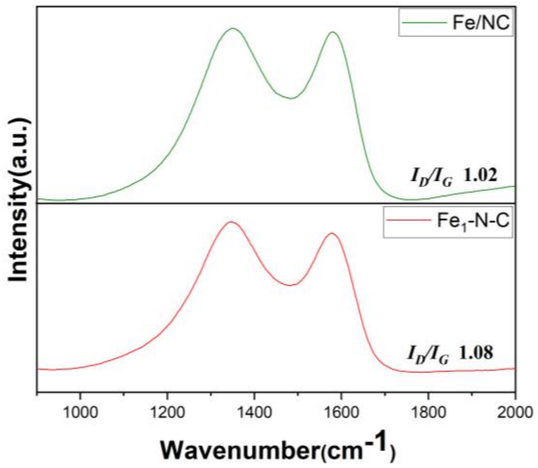

Supplementary Figure 8 Raman spectra. Fe_1-N-C and Fe/NC.

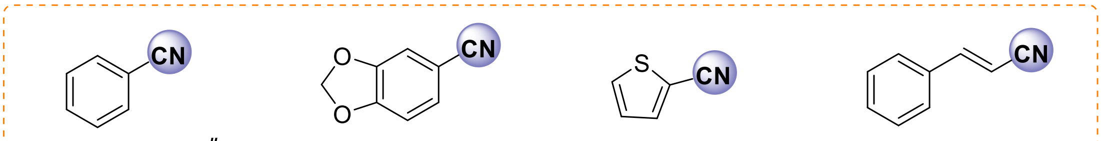

3a: 95%, 84%#   3r: 92%   3x: 85%   3aa: 88%

Supplementary Figure 9 Gram-scale syntheses of nitriles catalyzed by Fe_1-N-C. Reaction conditions: 20 mmol of substrate, 100 mL H_2O, 150 mg of aq. $NH_3.H_2O$ (25-28 wt%) for each 0.2 mmol substrate, 1 g Fe_1-N-C, 0.1 MPa of oxygen, 70°C, 24 h; #20 mmol of substrate, 100 mL H_2O, 150 mg of aq. $NH_3.H_2O$ for each 0.2 mmol substrate, 250 mg Fe_1-N-C, 2 MPa of air, 100°C, 10 h.

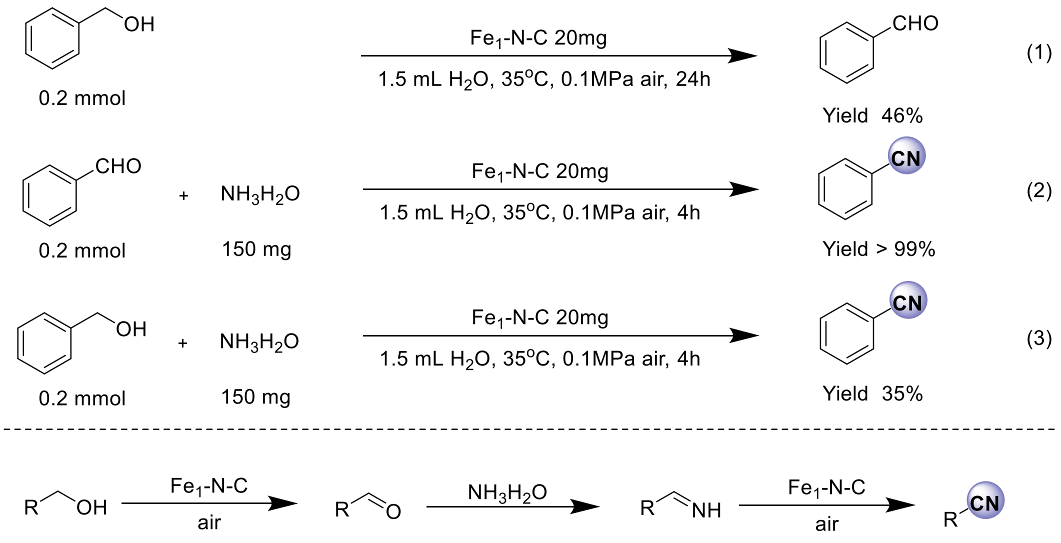

Supplementary Figure 10 Mechanistic studies. Control experiments and plausible reaction pathway for the ammoxidation of alcohol over Fe_1-N-C.

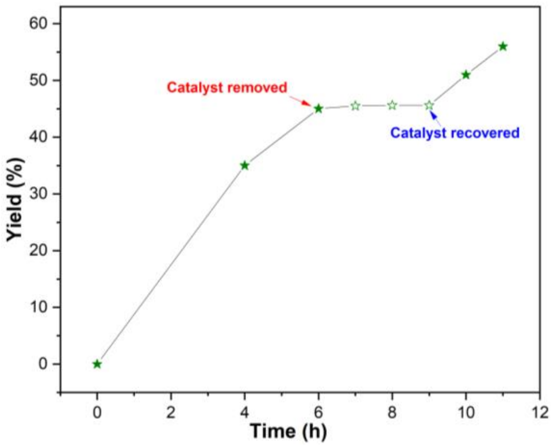

Supplementary Figure 11 Hot filtration experiments. The ammoxidation of alcohol over Fe_1-N-C.

NMR data

Benzonitrile (3a)

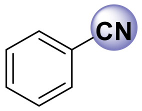

^{1}H NMR (500 MHz, Chloroform-d) δ 7.68 – 7.55 (m, 3H), 7.46 (t, J = 7.9 Hz, 2H). **^{13}C NMR** (126 MHz, Chloroform-d) δ 132.85, 132.17, 129.18, 118.90, 112.44.

p-Tolunitrile (3b)

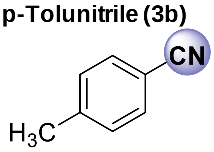

^{1}H NMR (300 MHz, Chloroform-d) δ 7.54 (d, J = 8.2 Hz, 2H), 7.27 (d, J = 7.9 Hz, 2H), 2.42 (s, 3H). **^{13}C NMR** (75 MHz, Chloroform-d) δ 143.74, 132.03, 129.86, 119.17, 109.28, 21.83.

Anisonitrile (3c)

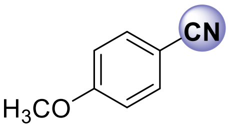

^{1}H NMR (300 MHz, Chloroform-d) δ 7.58 (d, *J* = 9.0 Hz, 2H), 6.94 (d, *J* = 9.0 Hz, 2H), 3.85 (s, 3H). **^{13}C NMR** (75 MHz, Chloroform-d) δ 162.86, 133.99, 119.25, 114.77, 103.95, 55.56.

m-Tolunitrile (3d)

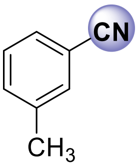

^{1}H NMR (300 MHz, Chloroform-d) δ 7.47 – 7.43 (m, 2H), 7.42 – 7.38 (m, 1H), 7.37 – 7.31 (m, 1H), 2.38 (s, 3H). **^{13}C NMR** (75 MHz, Chloroform-d) δ 139.24, 133.66, 132.49, 129.28, 129.00, 119.05, 112.24, 21.16.

3-Methoxybenzonitrile (3e)

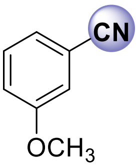

^{1}H NMR (300 MHz, Chloroform-d) δ 7.40 – 7.33 (m, 1H), 7.23 (dt, *J* = 7.6, 1.2 Hz, 1H), 7.15 – 7.09 (m, 2H), 3.82 (s, 3H). **^{13}C NMR** (75 MHz, Chloroform-d) δ 159.65, 130.35, 124.49, 119.32, 118.76, 116.86, 113.18, 55.54.

2-Methoxybenzonitrile (3f)

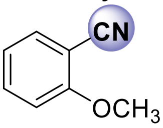

^{1}H NMR (300 MHz, Chloroform-d) δ 7.58 – 7.50 (m, 2H), 7.03 – 6.94 (m, 2H), 3.92 (s, 3H). **^{13}C NMR** (75 MHz, Chloroform-d) δ 161.24, 134.43, 133.75, 120.78, 116.54, 111.31, 101.77, 56.01.

3,4-Dimethoxybenzonitril (3g)

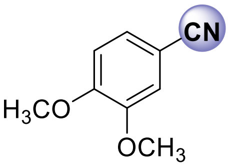

^{1}H NMR (500 MHz, DMSO-d6) δ 7.41 (dd, *J* = 8.4, 1.9 Hz, 1H), 7.38 (s, 1H), 7.11 (d, *J* = 8.4 Hz, 1H), 3.82 (d, *J* = 15.3 Hz, 6H). **^{13}C NMR** (126 MHz, DMSO-d6) δ 153.24, 149.40, 126.86, 119.75, 114.85, 112.53, 103.06, 56.35, 56.27.

3,4-Dimethylbenzonitrile (3h)

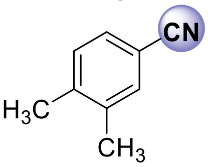

^{1}H NMR (500 MHz, Chloroform-d) δ 7.56 (s, 1H), 7.52 (d, *J* = 7.8 Hz, 1H), 7.32 (d, *J* = 7.8 Hz, 1H), 2.26 (d, *J* = 17.9 Hz, 6H). **^{13}C NMR** (126 MHz, Chloroform-d) δ 143.24, 138.52, 132.97, 130.85, 130.04, 119.59, 108.94, 20.07, 19.37.

4-Chlorobenzonitrile (3i)

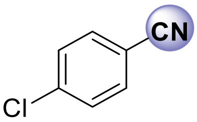

^{1}H NMR (300 MHz, Chloroform-d) δ 7.63 – 7.57 (m, 2H), 7.50 – 7.43 (m, 2H). **^{13}C NMR** (75 MHz, Chloroform-d) δ 139.57, 133.40, 129.72, 117.99, 110.80.

4-Bromobenzonitrile (3j)

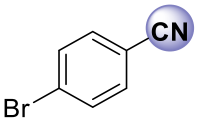

^{1}H NMR (300 MHz, Chloroform-d) δ 7.66 – 7.60 (m, 2H), 7.55 – 7.50 (m, 2H). **^{13}C NMR** (75 MHz, Chloroform-d) δ 133.43, 132.66, 128.04, 118.07, 111.26.

4-Nitrobenzonitrile (3k)

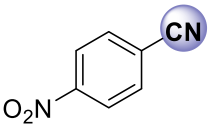

^{1}H NMR (400 MHz, Chloroform-d) δ 8.32 – 8.27 (m, 2H), 7.85 – 7.81 (m, 2H). **^{13}C NMR** (101 MHz, Chloroform-d) δ 150.05, 133.51, 124.31, 118.35, 116.83.

3,4,5-Trimethoxybenzonitrile (3l)

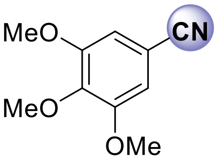

^{1}H NMR (500 MHz, Chloroform-d) δ 6.86 (s, 2H), 3.89 (d, *J* = 10.7 Hz, 9H). **^{13}C NMR** (126 MHz, Chloroform-d) δ 153.62, 142.21, 119.02, 109.51, 106.77, 61.11, 56.44.

4-Isopropylbenzonitrile (3n)

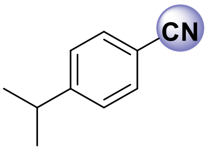

^{1}H NMR (500 MHz, Chloroform-d) δ 7.55 (d, *J* = 8.0 Hz, 2H), 7.29 (d, *J* = 8.0 Hz, 2H), 2.93 (hept, *J* = 7.0 Hz, 1H), 1.23 (d, *J* = 6.9 Hz, 6H). **^{13}C NMR** (126 MHz, Chloroform-d) δ 154.41, 132.27, 127.34, 119.22, 109.64, 34.43, 23.57.

Trifluoro-p-tolunitrile (3o)

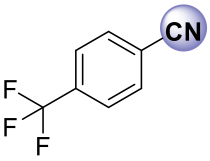

^{1}H NMR (500 MHz, Chloroform-d) δ 7.81 (d, *J* = 8.5 Hz, 2H), 7.76 (d, *J* = 8.5 Hz, 2H). **^{13}C NMR** (126 MHz, DMSO-d6) δ 133.78, 133.45, 133.19, 126.70, 126.67, 126.64, 126.61, 124.76, 122.59, 117.99, 115.96.

1-Naphthonitrile (3p)

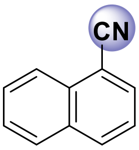

^{1}H NMR (300 MHz, Chloroform-d) δ 8.25 (ddt, *J* = 8.3, 1.5, 0.8 Hz, 1H), 8.09 (dt, *J* = 8.4, 1.1 Hz, 1H). **^{13}C NMR** (75 MHz, Chloroform-d) δ 133.31, 132.95, 132.66, 132.38, 128.68, 128.63, 127.58, 125.18, 124.95, 117.85, 110.21.

Quinoline-6-carbonitrile (3q)

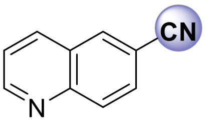

^{1}H NMR (500 MHz, DMSO-d6) δ 9.04 (s, 1H), 8.56 (s, 1H), 8.42 (d, *J* = 8.3 Hz, 1H), 8.09 (d, *J* = 8.7 Hz, 1H), 7.97 (d, *J* = 8.5 Hz, 1H), 7.63 (dd, *J* = 8.5, 4.0 Hz, 1H). **^{13}C NMR** (126 MHz, DMSO-d6) δ 153.99, 148.90, 137.11, 135.37, 130.84, 130.50, 127.70, 123.46, 119.05, 109.55.

Piperonylonitrile (3r)

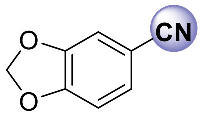

^{1}H NMR (500 MHz, DMSO-d6) δ 7.41 – 7.36 (m, 2H), 7.09 (d, *J* = 8.0 Hz, 1H), 6.16 (s, 2H). **^{13}C NMR** (126 MHz, DMSO-d6) δ 151.92, 148.29, 128.98, 119.37, 111.75, 109.81, 104.24, 102.94.

2,3-Dihydrobenzo[b][1,4]dioxine-6-carbonitrile (3s)

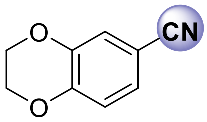

^{1}H NMR 1H NMR (500 MHz, DMSO-d6) δ 7.38 (d, *J* = 2.0 Hz, 1H), 7.29 (dd, *J* = 8.4, 2.0 Hz, 1H), 7.03 (d, *J* = 8.4 Hz, 1H), 4.34 – 4.32 (m, 2H), 4.30 – 4.28 (m, 2H). **^{13}C NMR** (126 MHz, DMSO-d6) δ 148.31, 144.24, 126.38, 121.37, 119.25, 118.77, 103.71, 64.98, 64.44.

4-Phenoxybenzonitrile (3t)

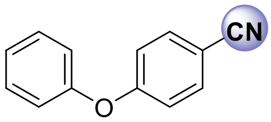

^{1}H NMR (500 MHz, DMSO-d6) δ 7.81 (d, *J* = 8.8 Hz, 2H), 7.46 (t, *J* = 7.9 Hz, 2H), 7.26 (t, *J* = 7.4 Hz, 1H), 7.12 (d, *J* = 8.0 Hz, 2H), 7.07 (d, *J* = 8.7 Hz, 2H). **^{13}C NMR** (126 MHz, DMSO-d6) δ 161.57, 154.88, 135.05, 130.89, 125.62, 120.71, 119.17, 118.41, 105.55.

2-Bromo-4,5-dimethoxybenzonitrile (3u)

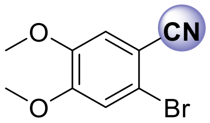

^{1}H NMR (500 MHz, Chloroform-d) δ 7.06 (s, 1H), 7.04 (s, 1H), 3.92 (s, 3H), 3.88 (s, 3H). **^{13}C NMR** (126 MHz, Chloroform-d) δ 153.23, 148.55, 117.68, 117.64, 115.53, 115.28, 106.91, 56.53, 56.41.

4-Cyano-1,1'-biphenyl (3v)

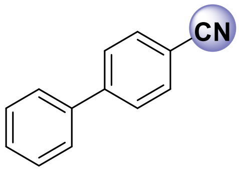

^{1}H NMR (500 MHz, DMSO-d6) δ 7.94 – 7.85 (m, 4H), 7.74 (d, *J* = 7.2 Hz, 2H), 7.51 (t, *J* = 7.5 Hz, 2H), 7.44 (t, *J* = 7.3 Hz, 1H). **^{13}C NMR** (126 MHz, DMSO-d6) δ 145.11, 138.73, 133.32, 129.64, 129.23, 128.04, 127.55, 119.34, 110.51.

p-Methoxybenzylnitrile (3w)

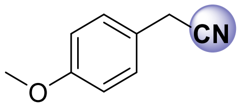

^{1}H NMR (500 MHz, Chloroform-d) δ 7.25 – 7.19 (m, 2H), 6.92 – 6.86 (m, 2H), 3.80 (s, 3H), 3.67 (s, 2H). **^{13}C NMR** (126 MHz, Chloroform-d) δ 159.38, 129.14, 121.87, 118.34, 114.56, 55.39, 22.82.

Thiophene-2-carbonitrile (3x)

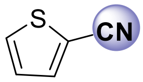

^{1}H NMR (400 MHz, Chloroform-d) δ 7.64 (dd, *J* = 3.8, 1.2 Hz, 1H), 7.61 (dd, *J* = 5.1, 1.2 Hz, 1H), 7.13 (dd, *J* = 5.1, 3.8 Hz, 1H). **^{13}C NMR** (101 MHz, Chloroform-d) δ 137.45, 132.61, 127.68, 114.27, 109.92.

2-Cyanopyridine (3y)

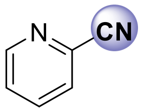

^{1}H NMR (400 MHz, Chloroform-d) δ 8.67 (ddd, *J* = 4.9, 1.8, 1.0 Hz, 1H), 7.79 (td, *J* = 7.8, 1.7 Hz, 1H), 7.65 (dt, *J* = 7.8, 1.1 Hz, 1H), 7.48 (ddd, *J* = 7.8, 4.8, 1.2 Hz, 1H). **^{13}C NMR** (101 MHz, Chloroform-d) δ 151.16, 137.06, 134.03, 128.57, 126.96, 117.19.

3-Cyanoquinoline (3z)

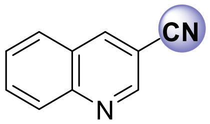

^{1}H NMR (500 MHz, DMSO-d6) δ 9.16 (d, *J* = 2.2 Hz, 1H), 9.07 (d, *J* = 2.2 Hz, 1H), 8.11 (t, *J* = 9.2 Hz, 2H), 7.97 (td, *J* = 7.6, 6.6, 1.6 Hz, 1H), 7.77 (t, *J* = 7.5 Hz, 1H). **^{13}C NMR** (126 MHz, DMSO-d6) δ 150.51, 148.49, 142.95, 133.49, 129.52, 129.33, 128.86, 126.34, 117.91, 106.28.

3-(3-Pyridinyl)-2-propenenitrile (3ab)

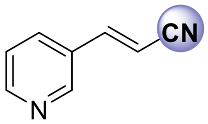

^{1}H NMR (500 MHz, DMSO-d6) δ 8.81 (d, *J* = 2.3 Hz, 1H), 8.62 (dd, *J* = 4.8, 1.6 Hz, 1H), 8.10 (dt, *J* = 8.0, 2.0 Hz, 1H), 7.71 (d, *J* = 16.7 Hz, 1H), 7.48 (dd, *J* = 8.0, 4.8 Hz, 1H), 6.63 (d, *J* = 16.8 Hz, 1H). **^{13}C NMR** (126 MHz, DMSO-d6) δ 152.03, 149.72, 148.05, 134.59, 130.11, 124.46, 118.83, 99.46.

2-methyl-3-phenyl-acrylonitrile (3ac)

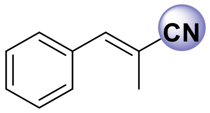

^{1}H NMR 1H NMR (500 MHz, Chloroform-d) δ 7.44 – 7.39 (m, 2H), 7.39 – 7.36 (m, 1H), 7.33 (d, *J* = 7.5 Hz, 2H), 7.21 (s, 1H), 2.15 (s, 3H). **^{13}C NMR** (126 MHz, Chloroform-d) δ 144.51, 134.16, 129.38, 129.34, 128.74, 121.34, 109.70, 16.86.

NMR spectra

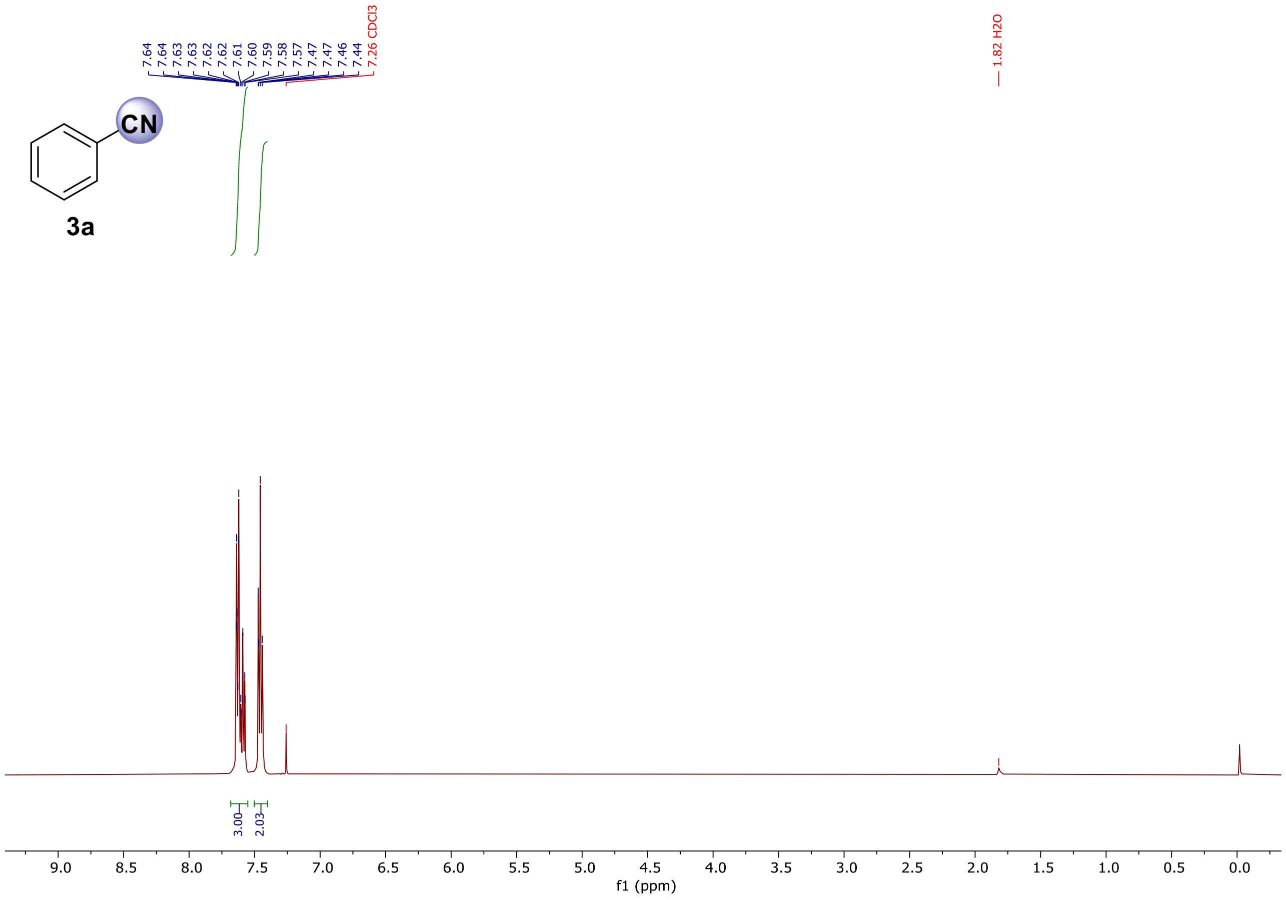

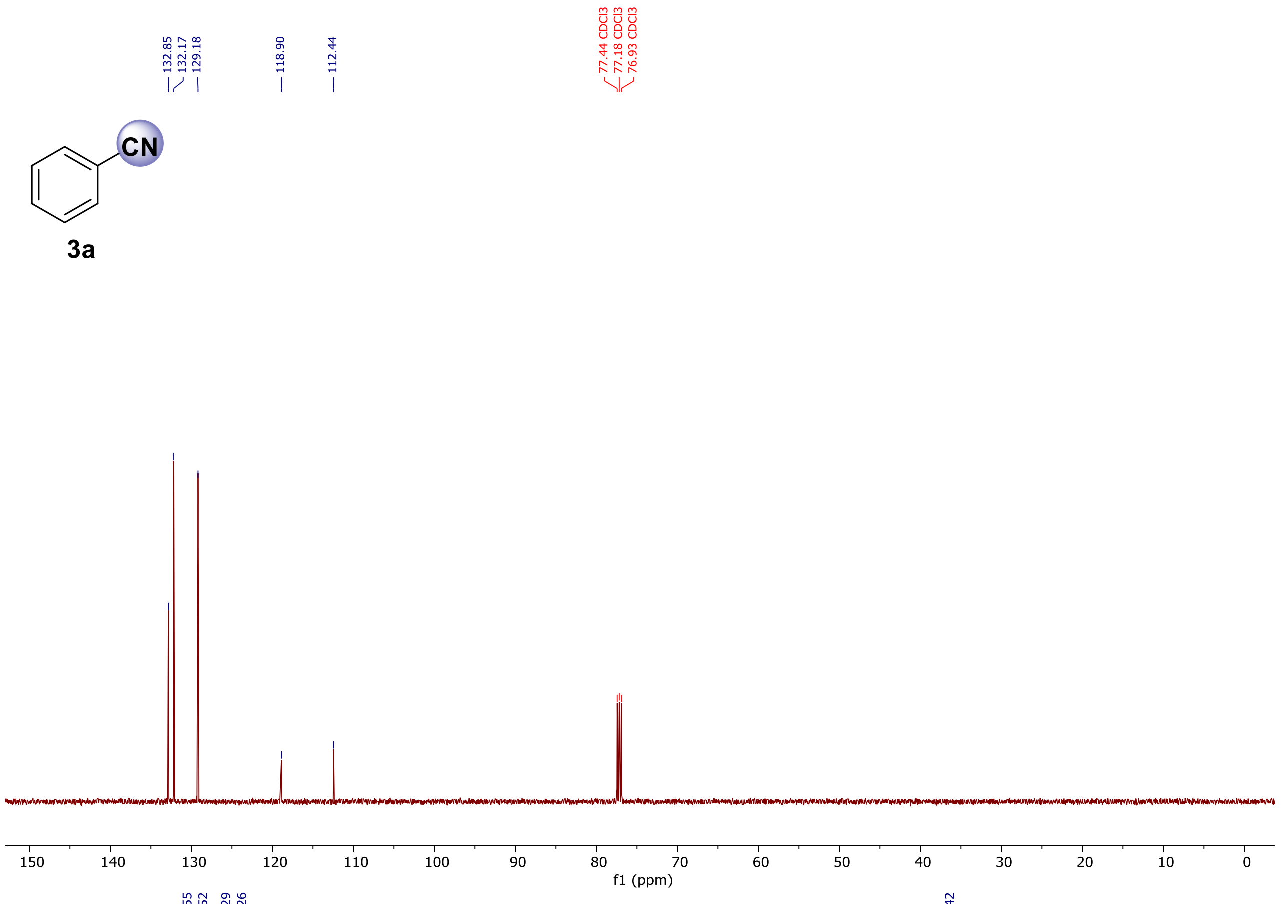
132.85
132.17
129.18
118.90
112.44
77.44 CDCl3
77.18 CDCl3
76.93 CDCl3
CN
3a
150 140 130 120 110 100 90 80 70 60 50 40 30 20 10 0
f1 (ppm)

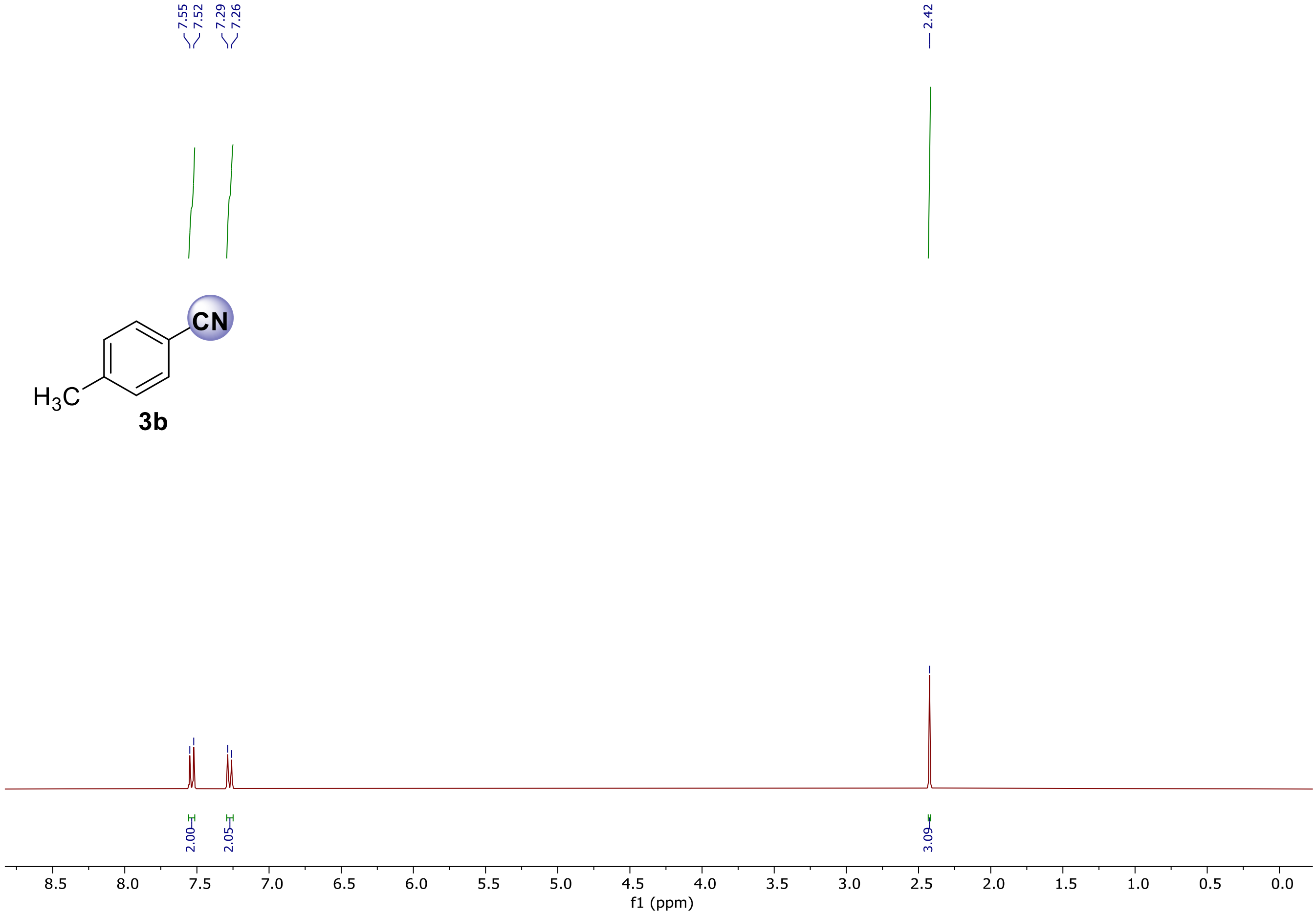
7.55
7.52
7.29
7.26
2.42
CN
H3C
3b
2.00
2.05
3.09
8.5 8.0 7.5 7.0 6.5 6.0 5.5 5.0 4.5 4.0 3.5 3.0 2.5 2.0 1.5 1.0 0.5 0.0
f1 (ppm)

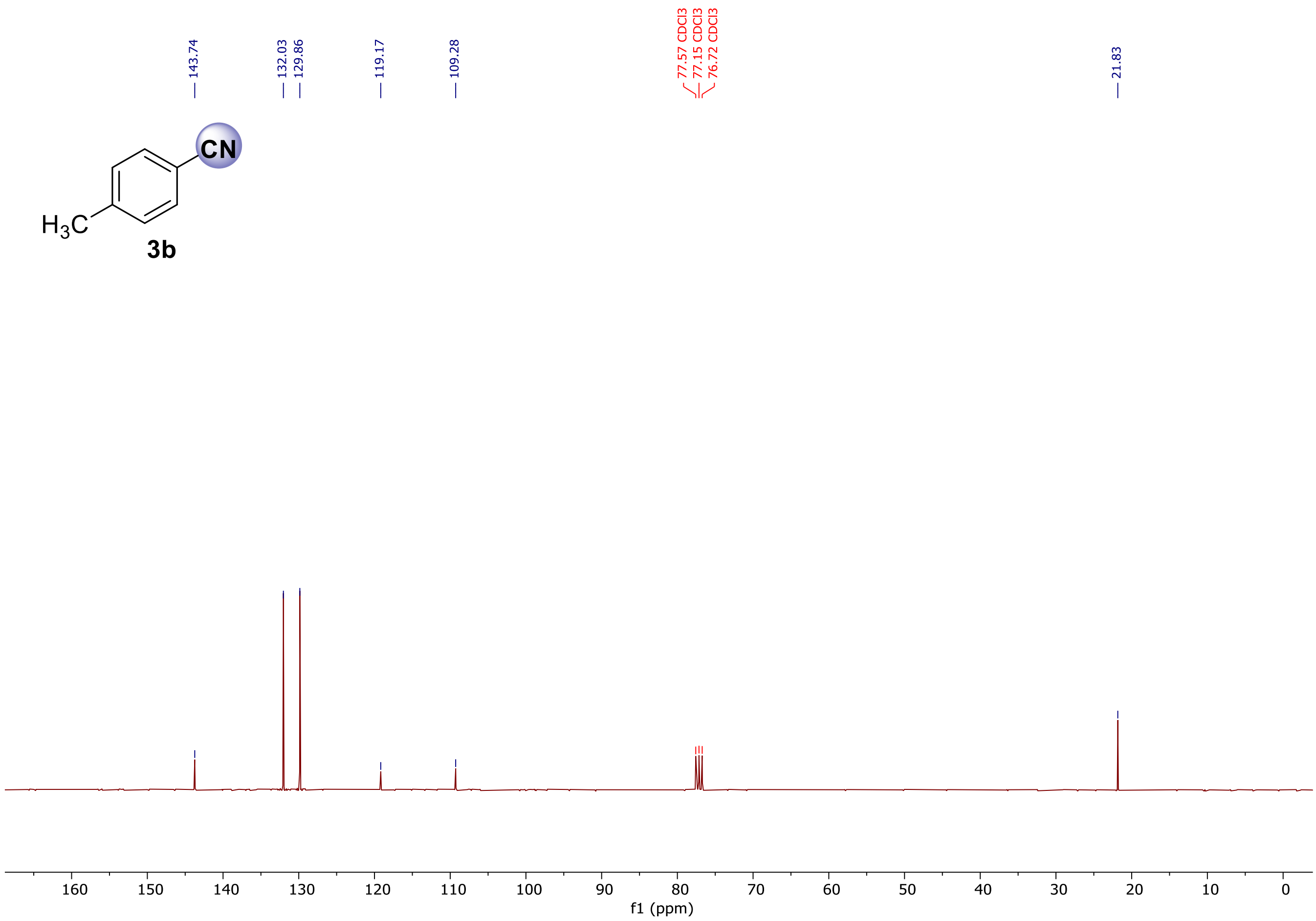
143.74
132.03
129.86
119.17
109.28
77.57 CDCl3
77.15 CDCl3
76.72 CDCl3
21.83
CN
H_3C
3b
160 150 140 130 120 110 100 90 80 70 60 50 40 30 20 10 0
f1 (ppm)

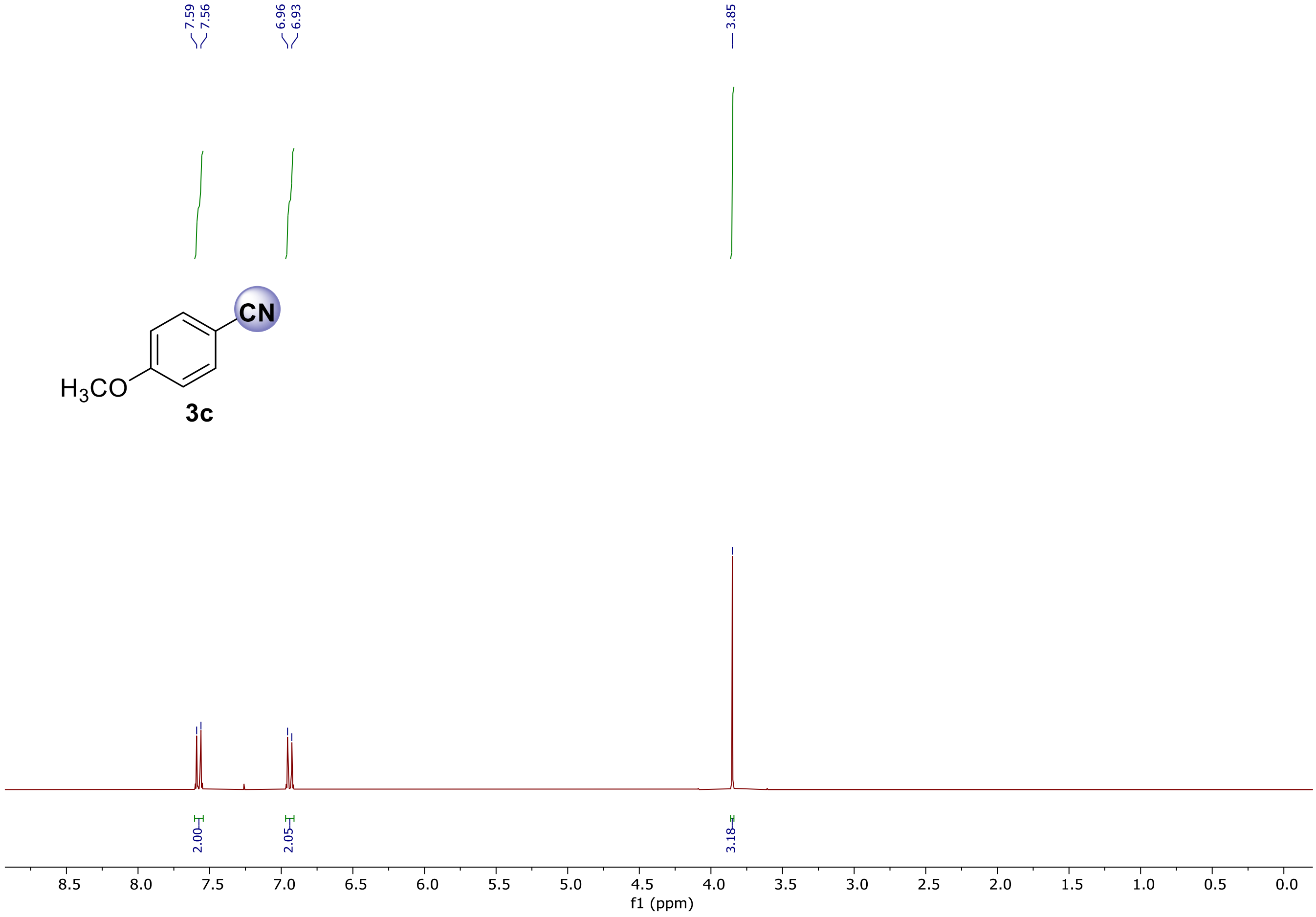
7.59
7.56
6.96
6.93
3.85
CN
H_3CO
3c
2.00
2.05
3.18
8.5 8.0 7.5 7.0 6.5 6.0 5.5 5.0 4.5 4.0 3.5 3.0 2.5 2.0 1.5 1.0 0.5 0.0
f1 (ppm)

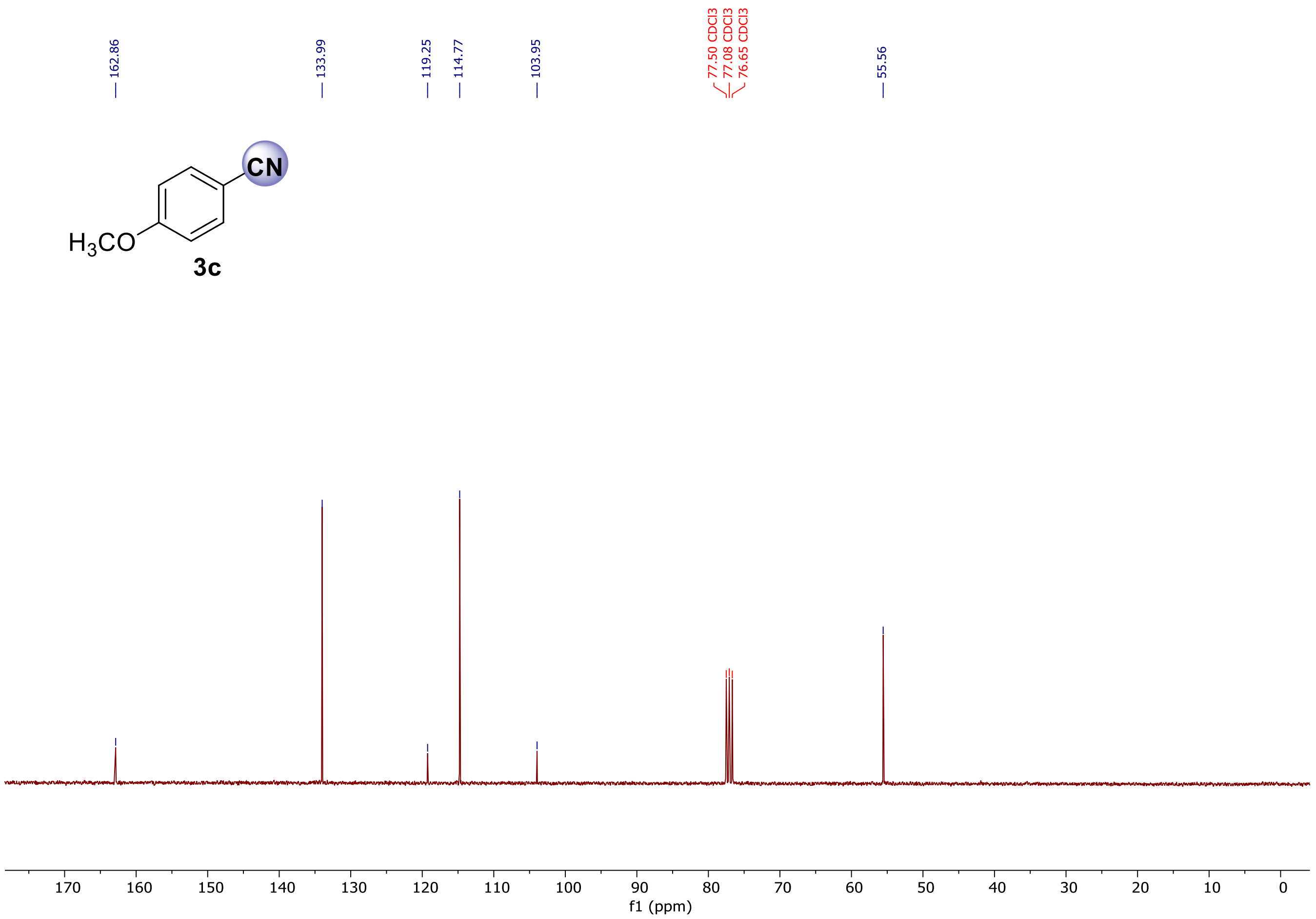
162.86
133.99
119.25
114.77
103.95
77.50 CDCl3
77.08 CDCl3
76.65 CDCl3
55.56
CN
H3CO
3c
170 160 150 140 130 120 110 100 90 80 70 60 50 40 30 20 10 0
f1 (ppm)

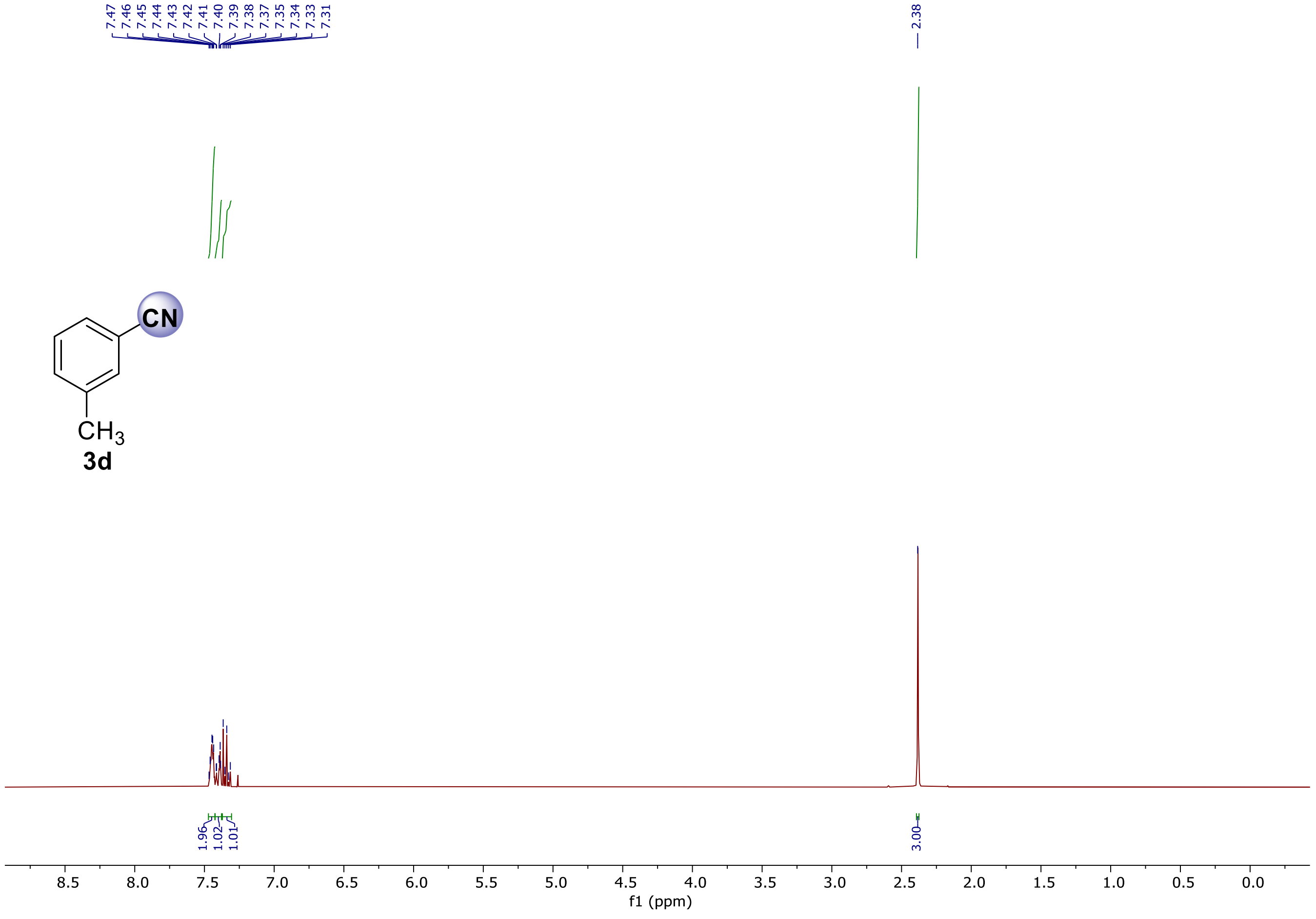
7.47
7.46
7.45
7.44
7.43
7.42
7.41
7.40
7.39
7.38
7.37
7.35
7.34
7.33
7.31
2.38
CN
CH3
3d
1.96
1.02
1.01
3.00
8.5 8.0 7.5 7.0 6.5 6.0 5.5 5.0 4.5 4.0 3.5 3.0 2.5 2.0 1.5 1.0 0.5 0.0
f1 (ppm)

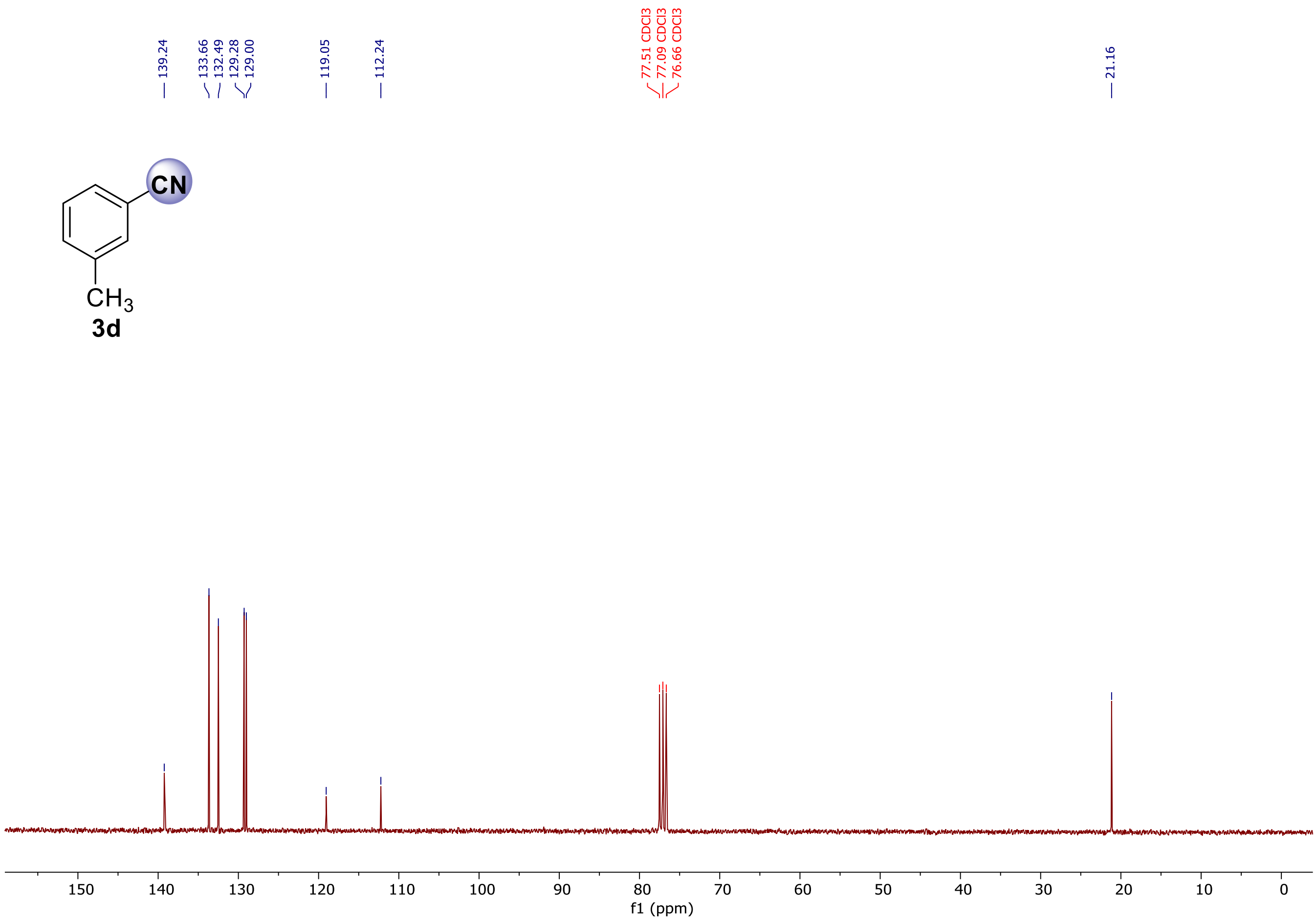
139.24
133.66
132.49
129.28
129.00
119.05
112.24
77.51 CDCl3
77.09 CDCl3
76.66 CDCl3
21.16
CN
CH3
3d
150 140 130 120 110 100 90 80 70 60 50 40 30 20 10 0
f1 (ppm)

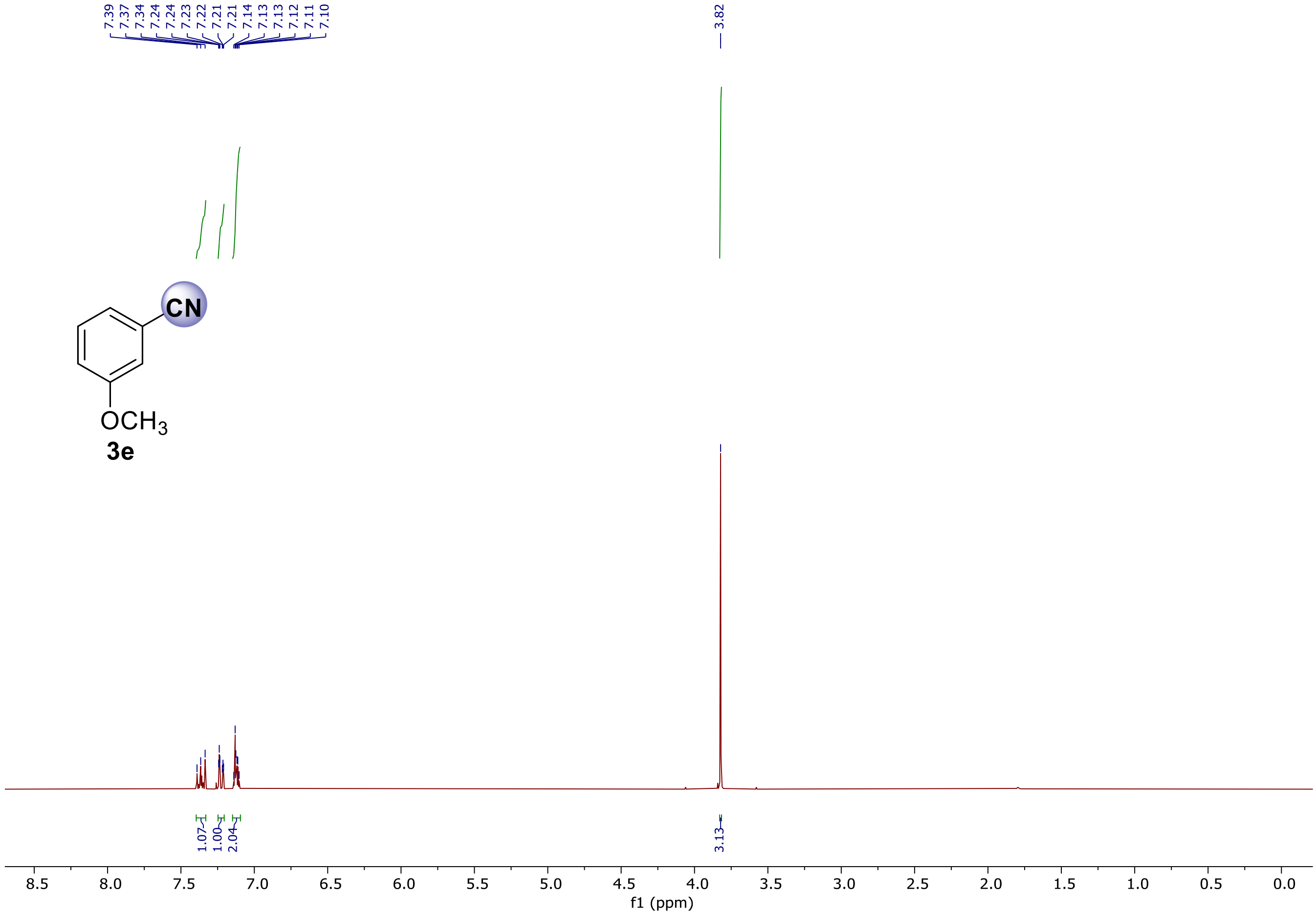
7.39
7.37
7.34
7.24
7.24
7.23
7.22
7.21
7.21
7.14
7.13
7.13
7.12
7.11
7.10
3.82
CN
OCH3
3e
1.07
1.00
2.04
3.13
8.5 8.0 7.5 7.0 6.5 6.0 5.5 5.0 4.5 4.0 3.5 3.0 2.5 2.0 1.5 1.0 0.5 0.0
f1 (ppm)

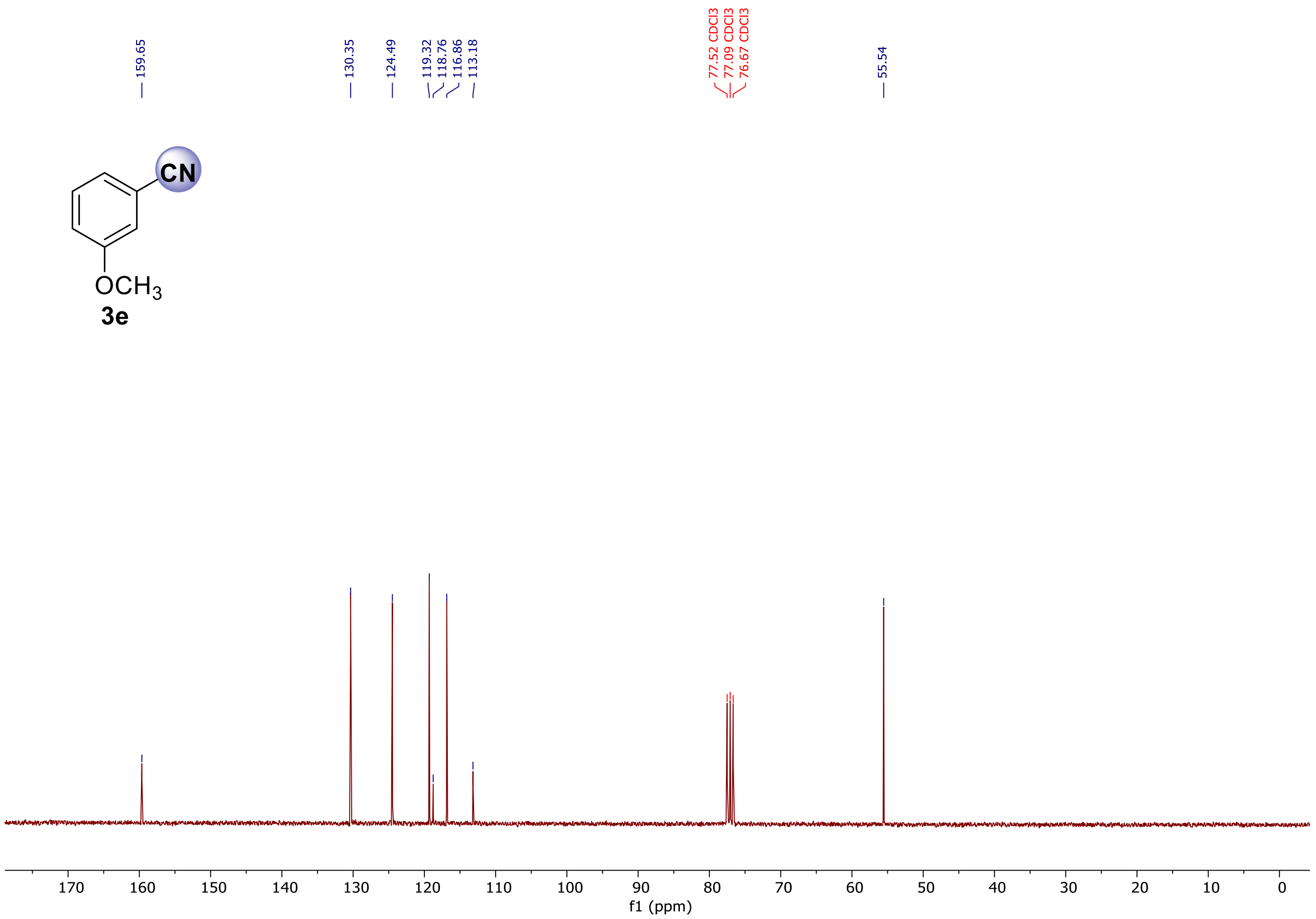

159.65
130.35
124.49
119.32
118.76
116.86
113.18
77.52 CDCl3
77.09 CDCl3
76.67 CDCl3
55.54
CN
OCH3
3e
f1 (ppm)

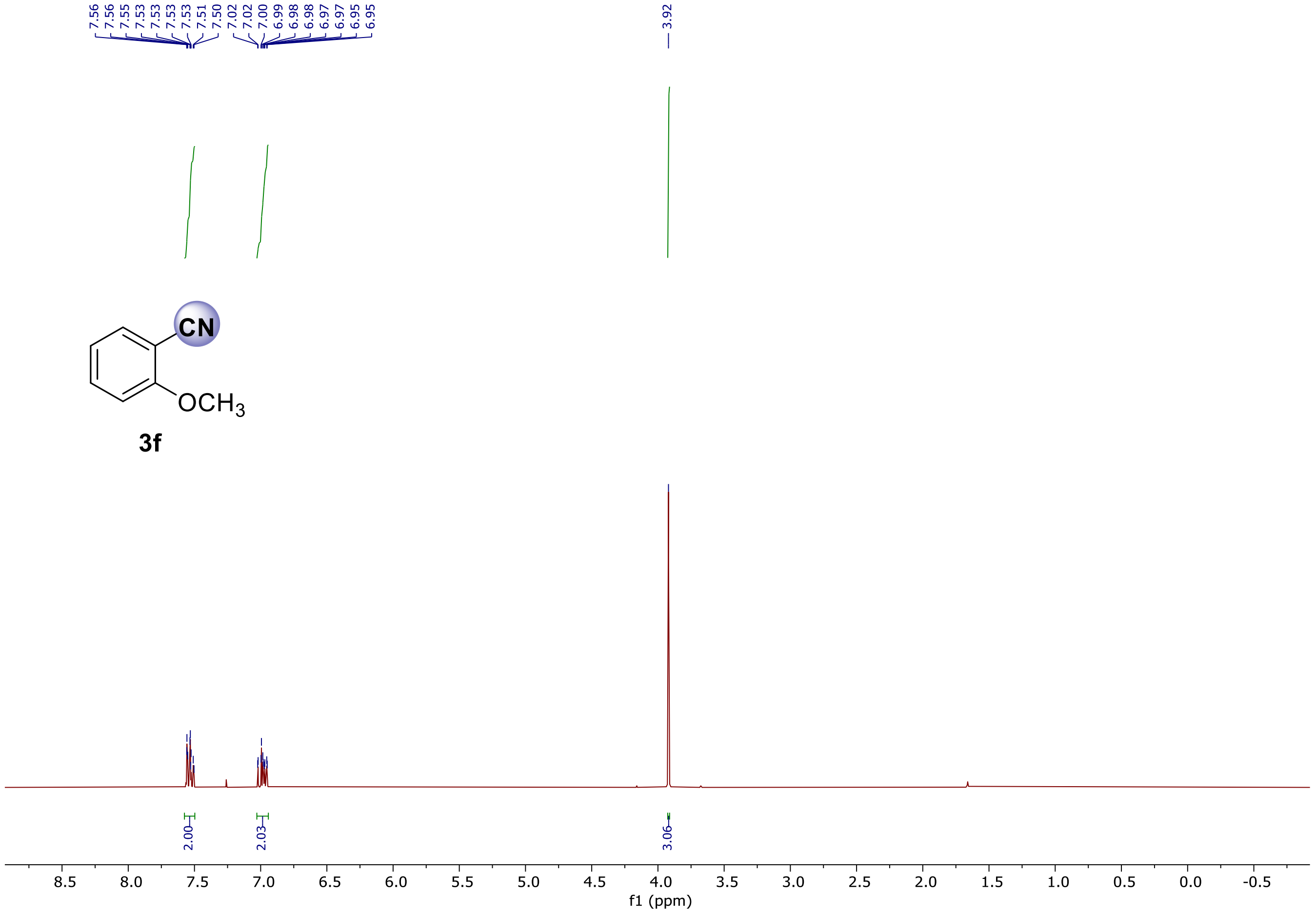

7.56
7.56
7.55
7.53
7.53
7.53
7.53
7.51
7.50
7.02
7.02
7.00
6.99
6.98
6.98
6.97
6.97
6.95
6.95
3.92
CN
OCH3
3f
2.00
2.03
3.06
f1 (ppm)

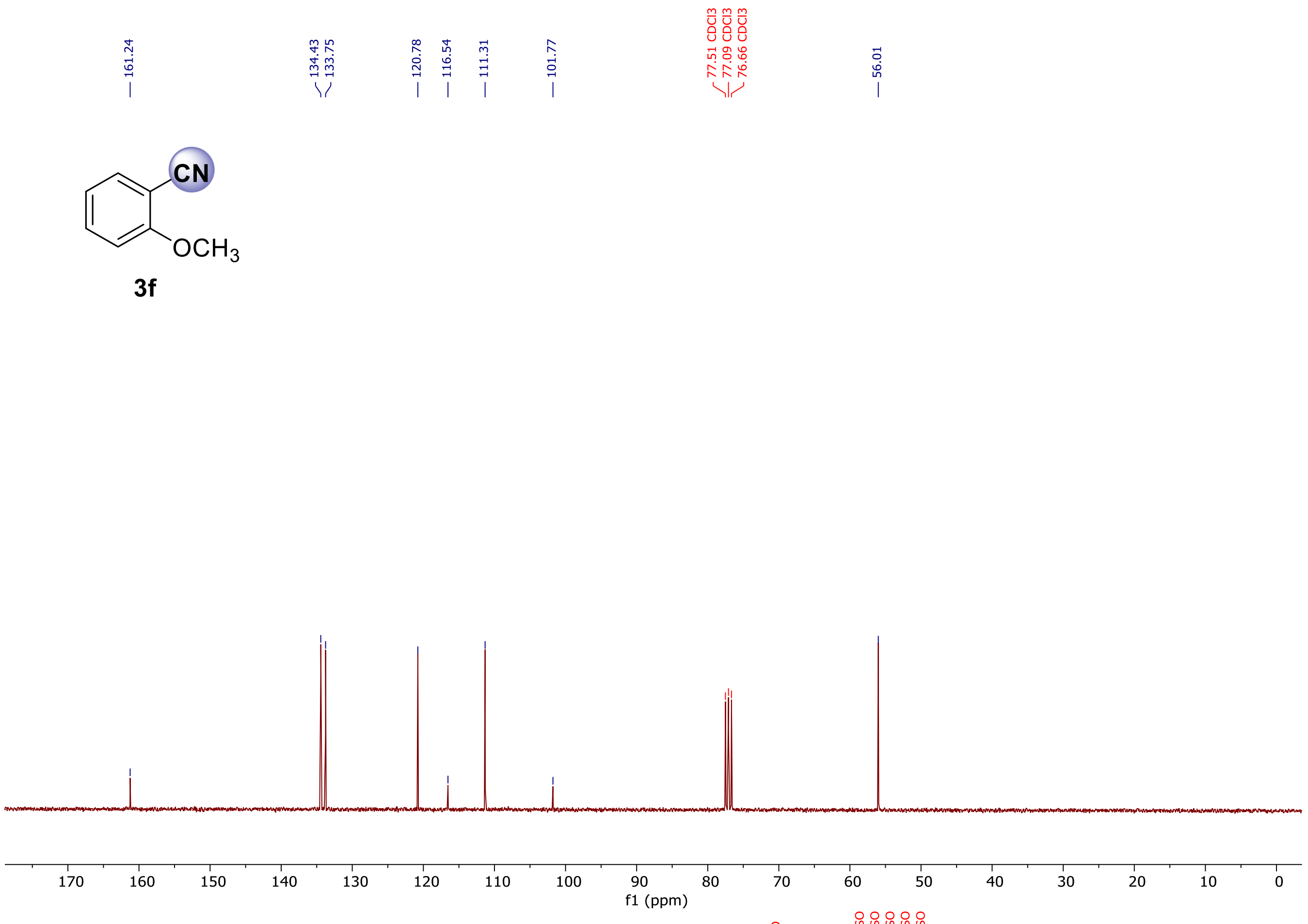
161.24
134.43
133.75
120.78
116.54
111.31
101.77
77.51 CDCl3
77.09 CDCl3
76.66 CDCl3
56.01
CN
OCH3
3f
170 160 150 140 130 120 110 100 90 80 70 60 50 40 30 20 10 0
f1 (ppm)

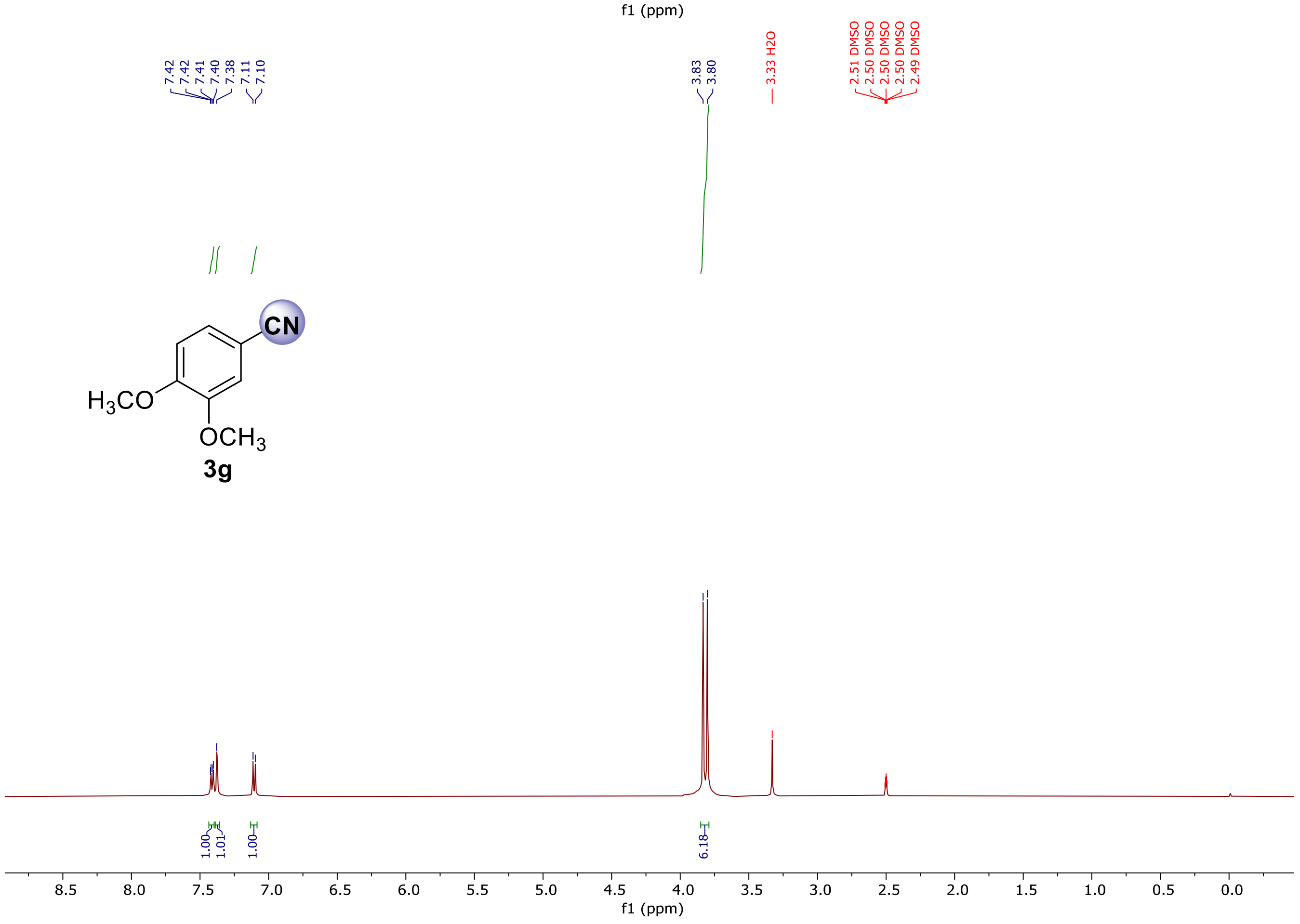
7.42
7.42
7.41
7.40
7.38
7.11
7.10
3.83
3.80
3.33 H2O
2.51 DMSO
2.50 DMSO
2.50 DMSO
2.50 DMSO
2.49 DMSO
CN
H3CO
OCH3
3g
1.00
1.01
1.00
6.18
8.5 8.0 7.5 7.0 6.5 6.0 5.5 5.0 4.5 4.0 3.5 3.0 2.5 2.0 1.5 1.0 0.5 0.0
f1 (ppm)

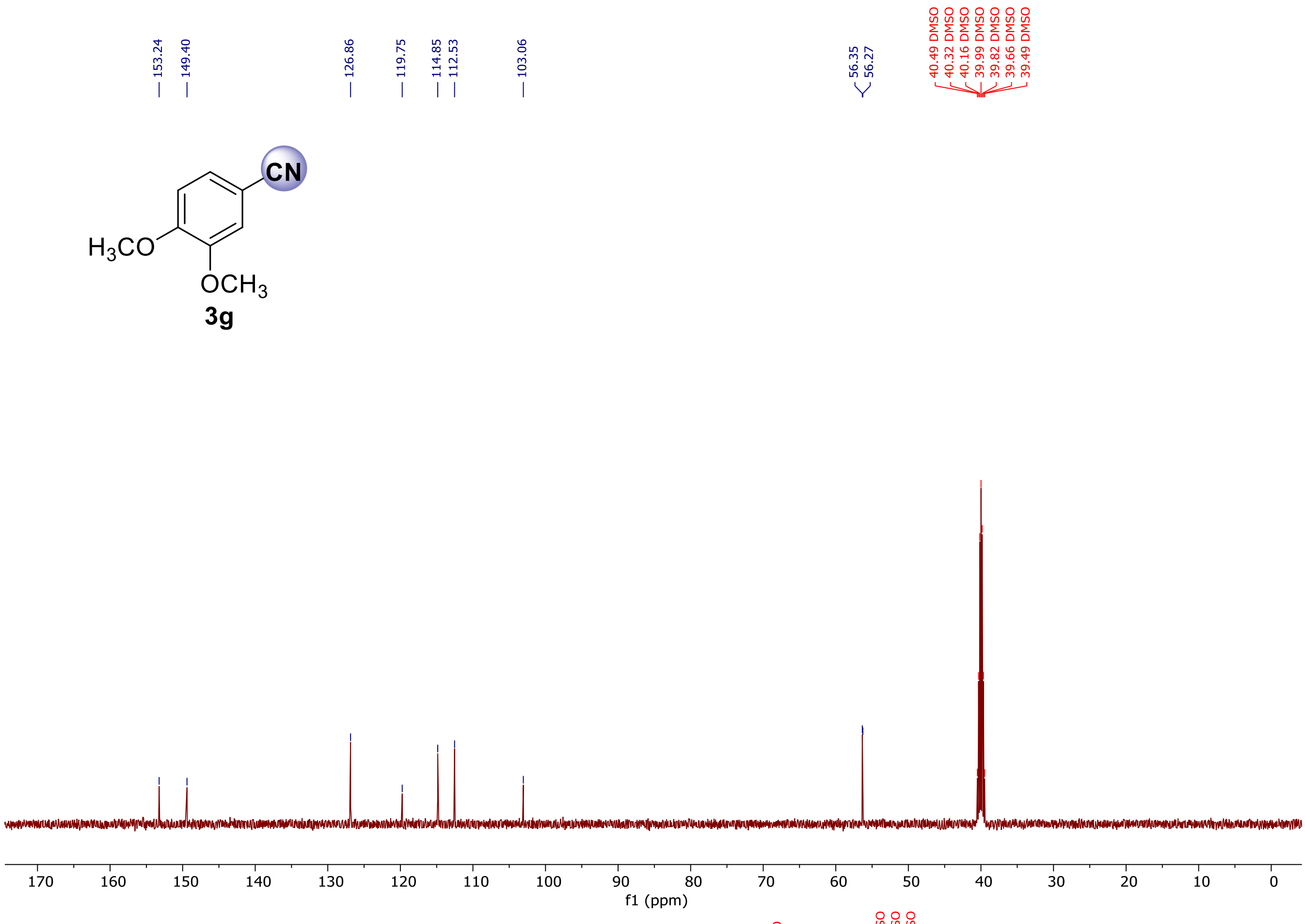

153.24
149.40
126.86
119.75
114.85
112.53
103.06
56.35
56.27
40.49 DMSO
40.32 DMSO
40.16 DMSO
39.99 DMSO
39.82 DMSO
39.66 DMSO
39.49 DMSO
CN
H3CO
OCH3
3g
170 160 150 140 130 120 110 100 90 80 70 60 50 40 30 20 10 0
f1 (ppm)

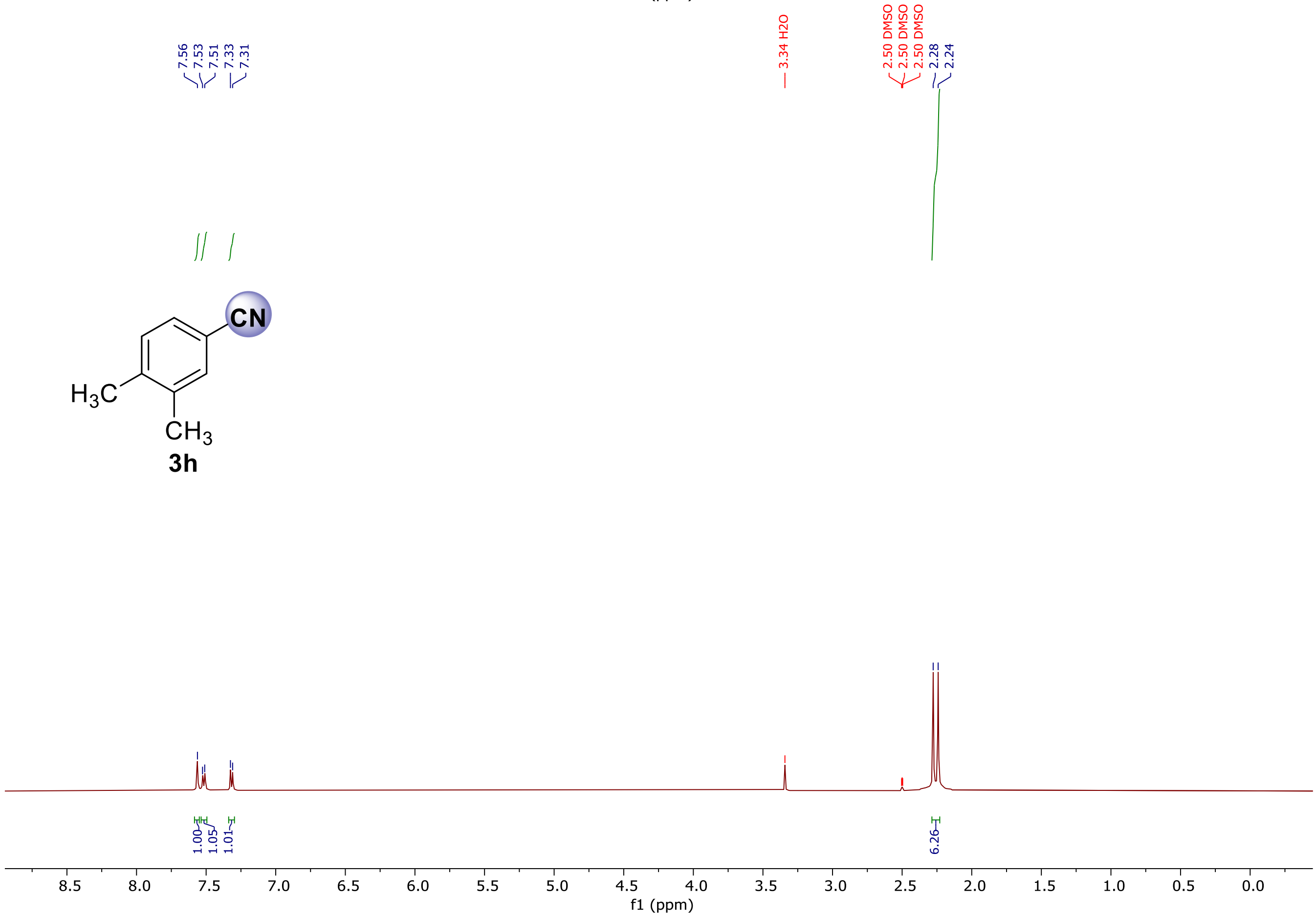

7.56
7.53
7.51
7.33
7.31
3.34 H2O
2.50 DMSO
2.50 DMSO
2.50 DMSO
2.28
2.24
CN
H3C
CH3
3h
1.00
1.05
1.01
6.26
8.5 8.0 7.5 7.0 6.5 6.0 5.5 5.0 4.5 4.0 3.5 3.0 2.5 2.0 1.5 1.0 0.5 0.0
f1 (ppm)

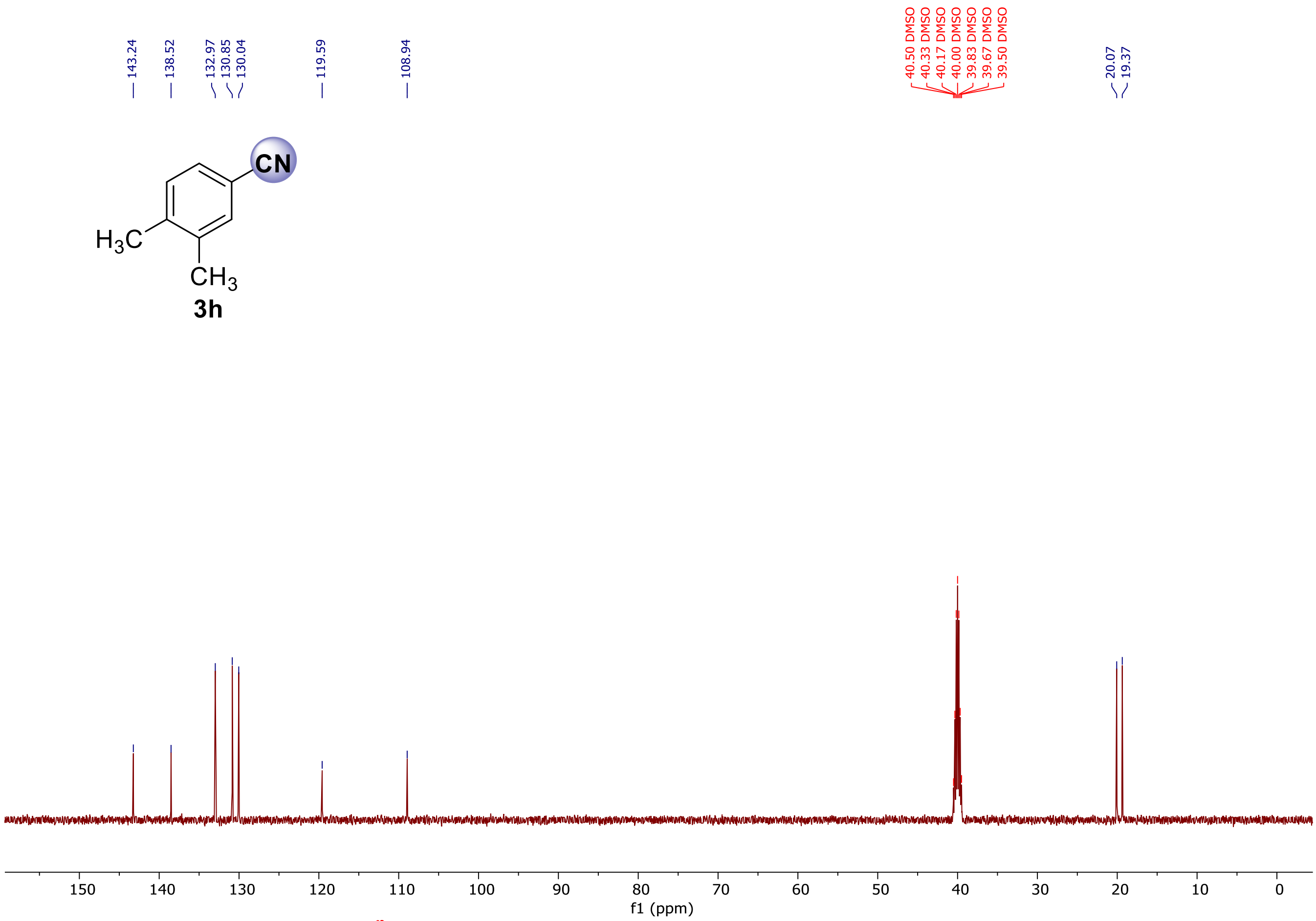
143.24
138.52
132.97
130.85
130.04
119.59
108.94
40.50 DMSO
40.33 DMSO
40.17 DMSO
40.00 DMSO
39.83 DMSO
39.67 DMSO
39.50 DMSO
20.07
19.37
CN
H_3C
CH_3
3h
f1 (ppm)

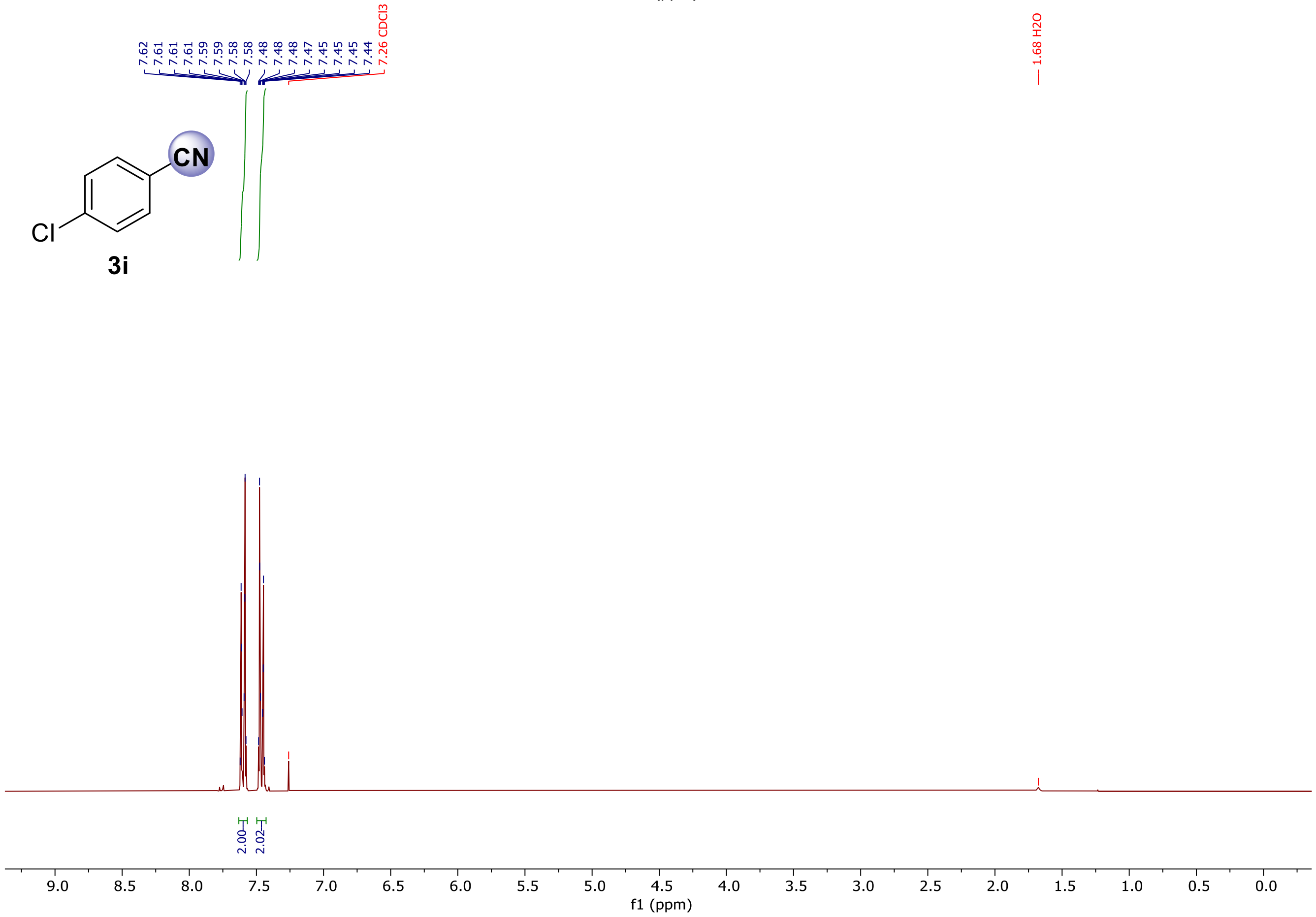
7.62
7.61
7.61
7.61
7.59
7.59
7.58
7.58
7.48
7.48
7.48
7.47
7.45
7.45
7.45
7.44
7.26 CDCl3
1.68 H2O
CN
Cl
3i
2.00
2.02
f1 (ppm)

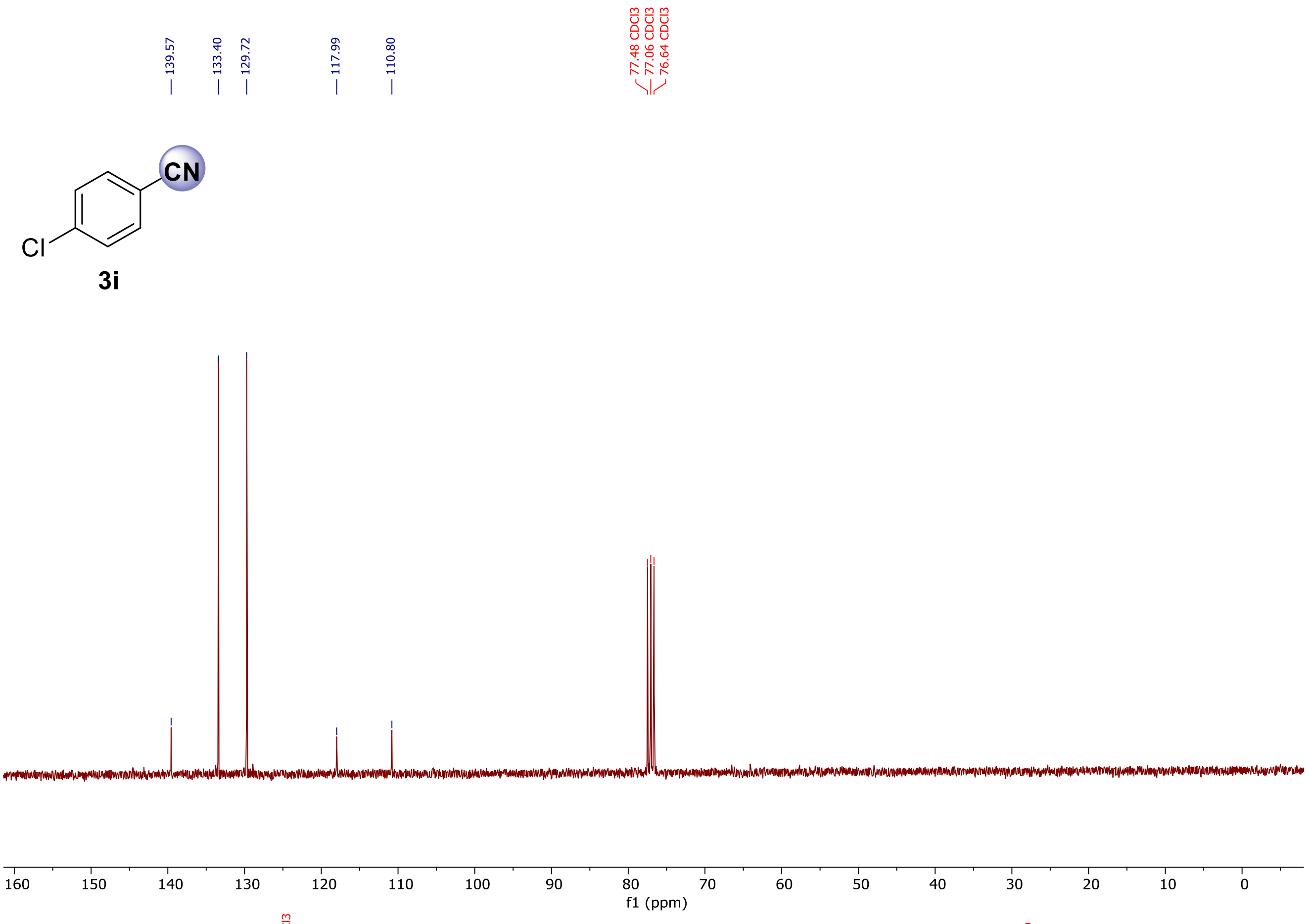
139.57
133.40
129.72
117.99
110.80
77.48 CDCl3
77.06 CDCl3
76.64 CDCl3
CN
Cl
3i
160 150 140 130 120 110 100 90 80 70 60 50 40 30 20 10 0
f1 (ppm)

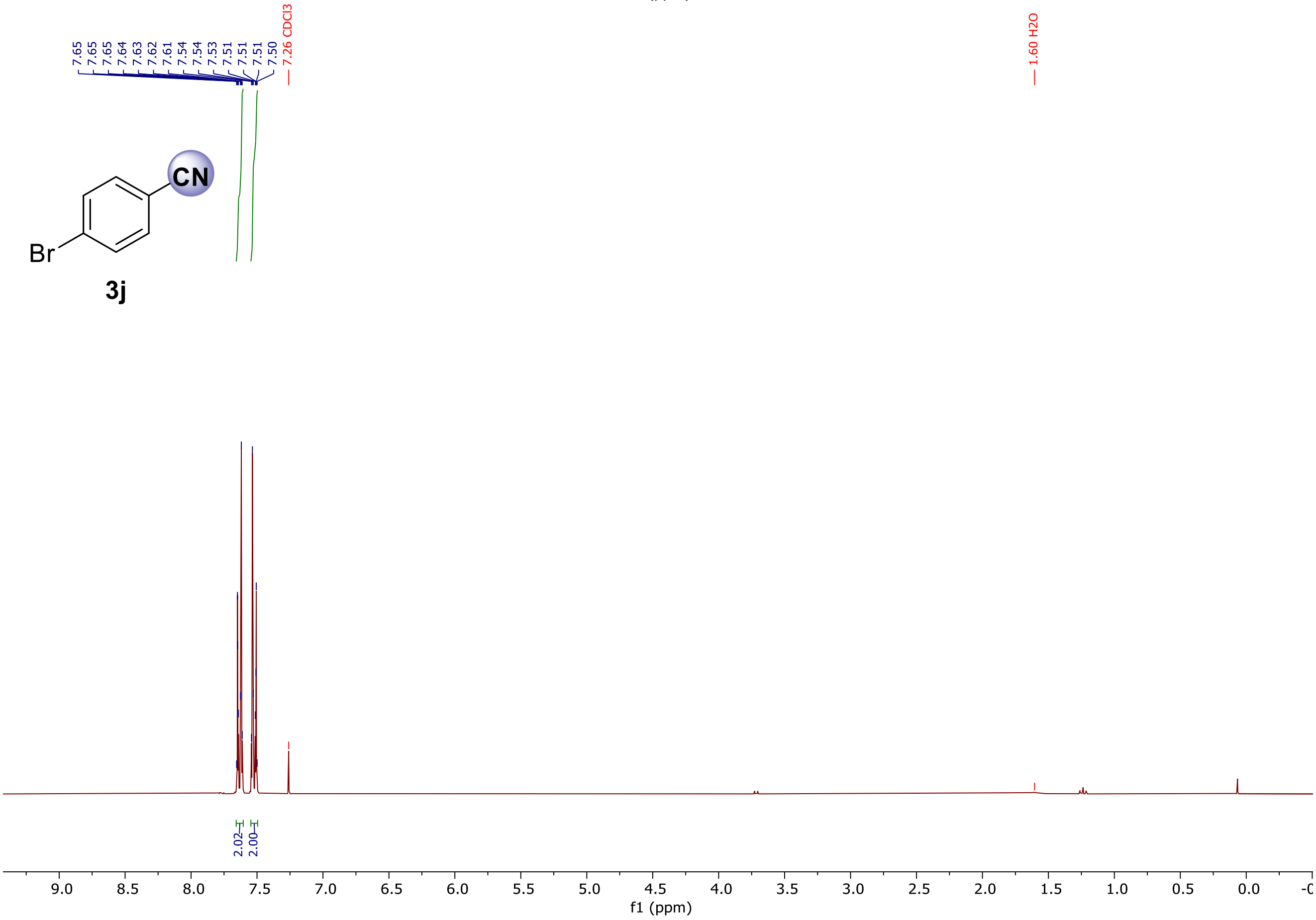
7.65
7.65
7.65
7.64
7.63
7.62
7.61
7.54
7.54
7.53
7.51
7.51
7.51
7.50
7.26 CDCl3
1.60 H2O
CN
Br
3j
2.02
2.00
9.0 8.5 8.0 7.5 7.0 6.5 6.0 5.5 5.0 4.5 4.0 3.5 3.0 2.5 2.0 1.5 1.0 0.5 0.0
f1 (ppm)

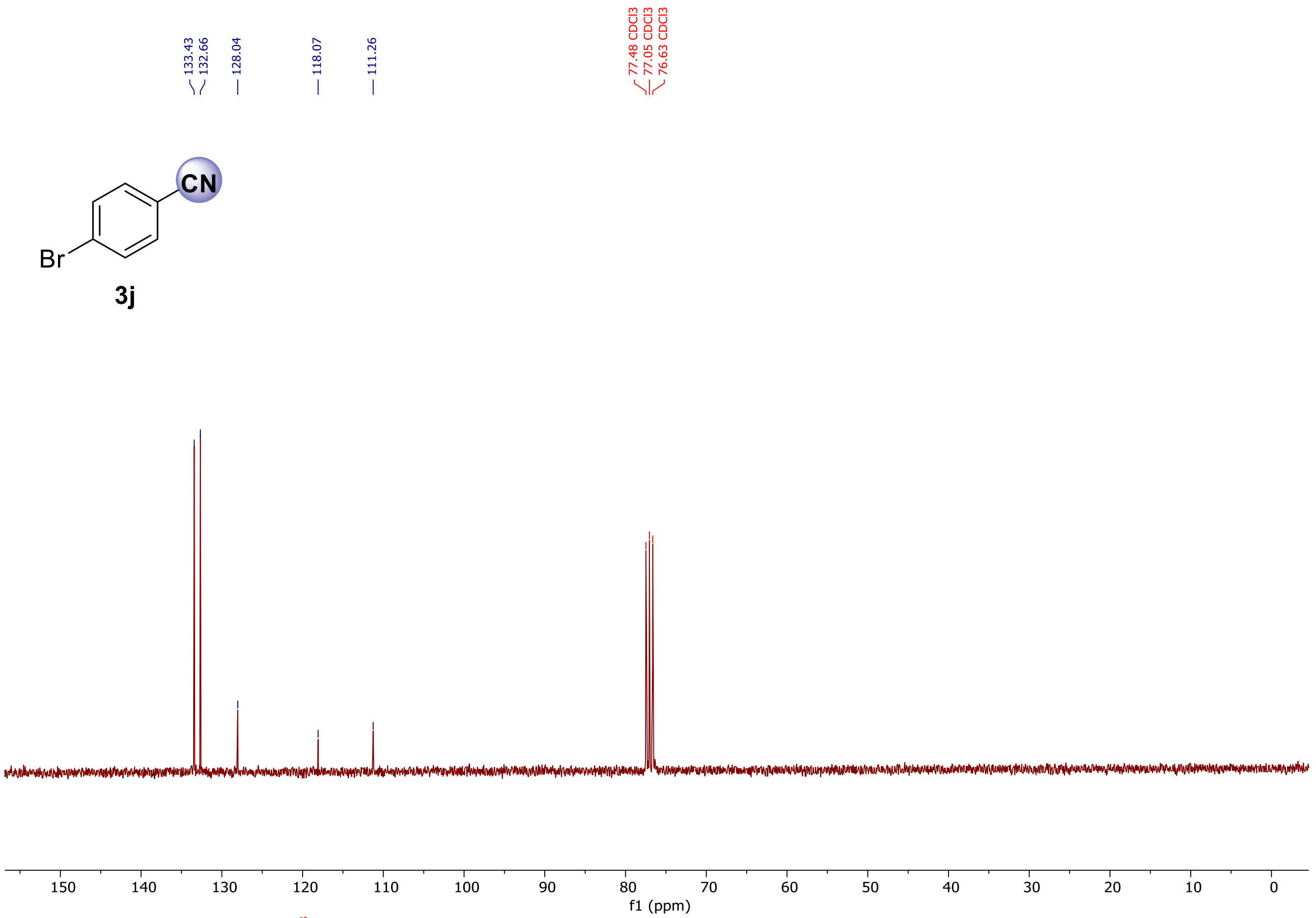
133.43
132.66
128.04
118.07
111.26
77.48 CDCl3
77.05 CDCl3
76.63 CDCl3
CN
Br
3j
150 140 130 120 110 100 90 80 70 60 50 40 30 20 10 0
f1 (ppm)

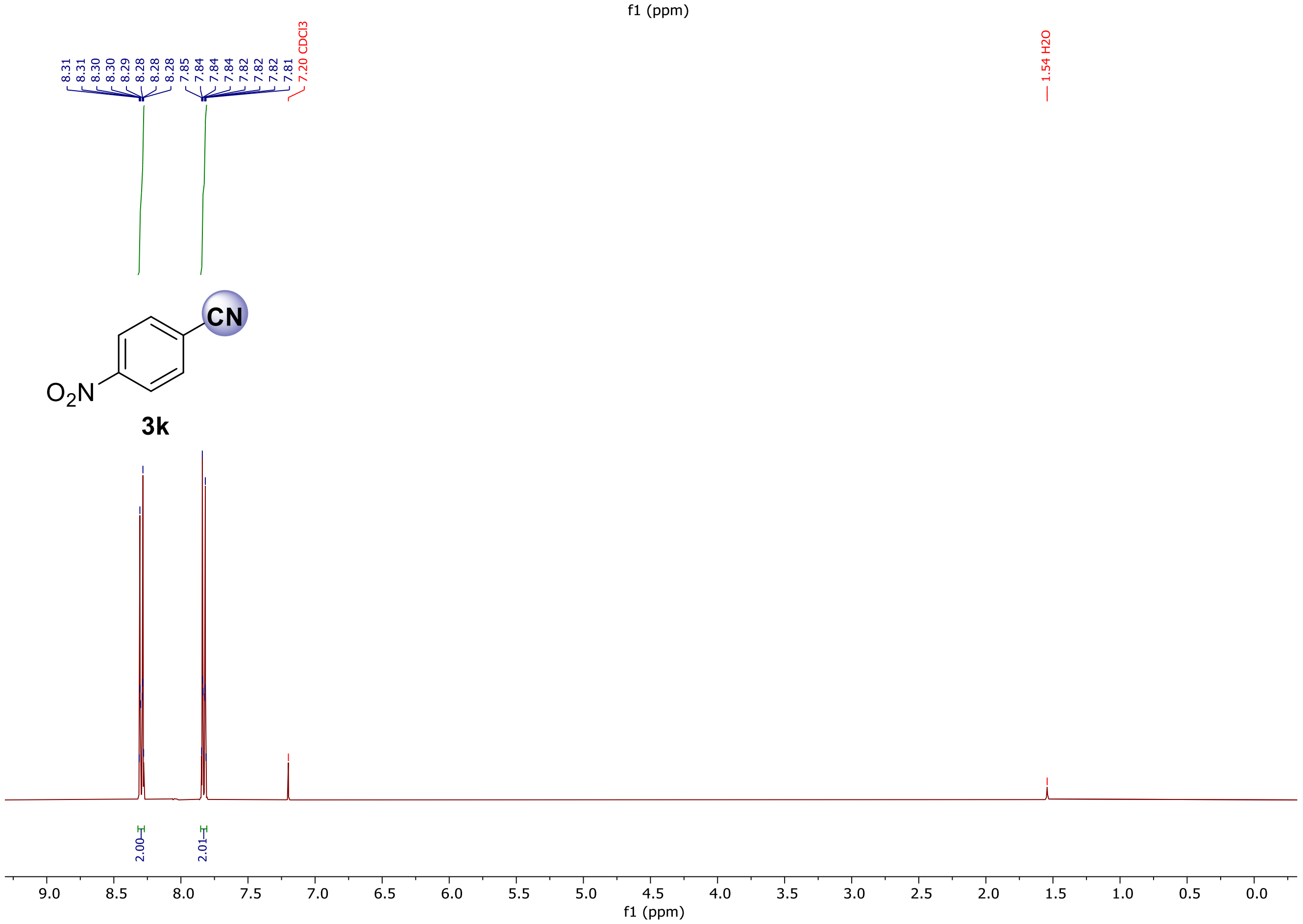
8.31
8.31
8.30
8.30
8.29
8.28
8.28
8.28
7.85
7.84
7.84
7.84
7.82
7.82
7.82
7.81
7.20 CDCl3
1.54 H2O
CN
O2N
3k
2.00
2.01
9.0 8.5 8.0 7.5 7.0 6.5 6.0 5.5 5.0 4.5 4.0 3.5 3.0 2.5 2.0 1.5 1.0 0.5 0.0
f1 (ppm)

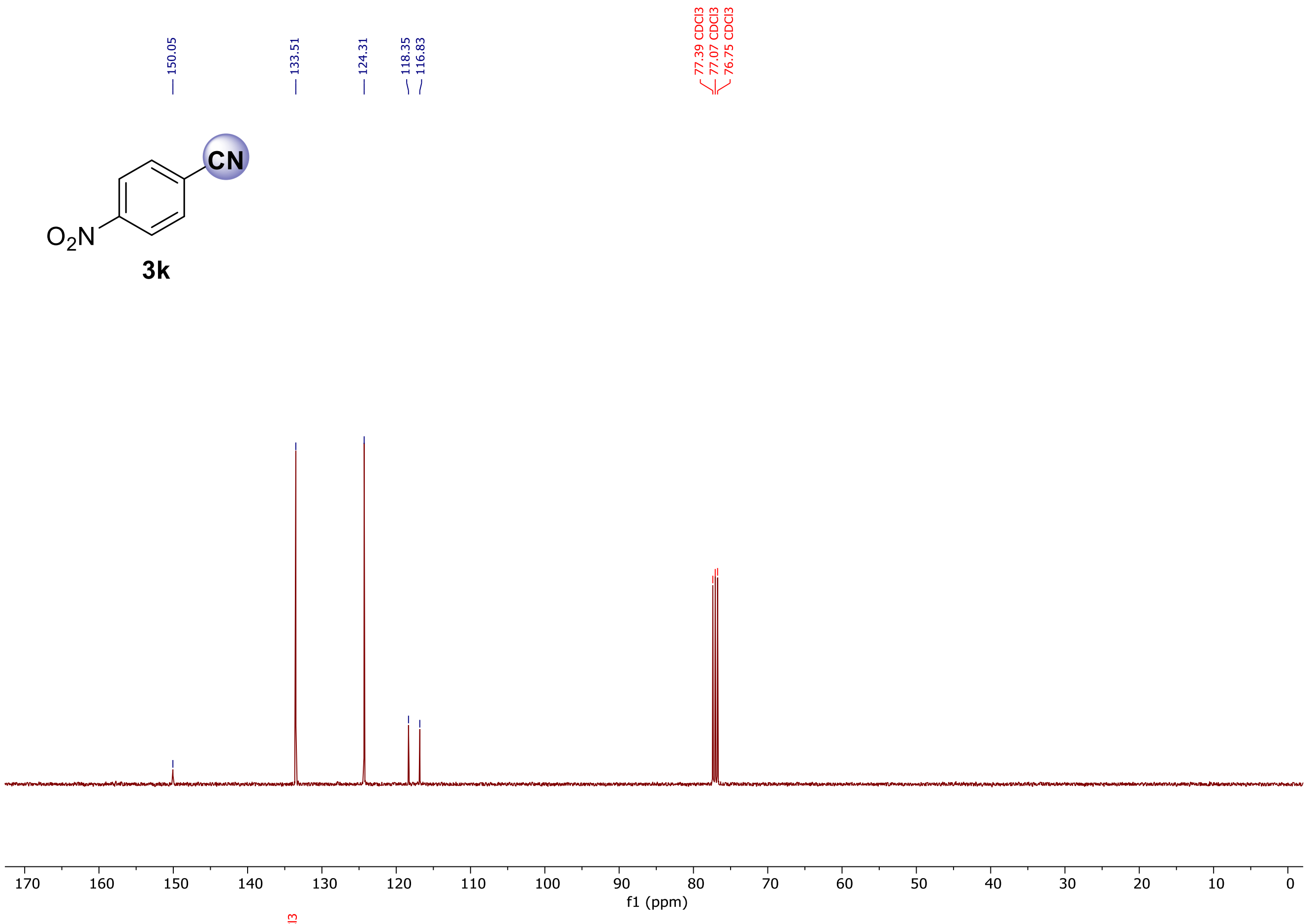
150.05
133.51
124.31
118.35
116.83
77.39 CDCl3
77.07 CDCl3
76.75 CDCl3
CN
O2N
3k
f1 (ppm)

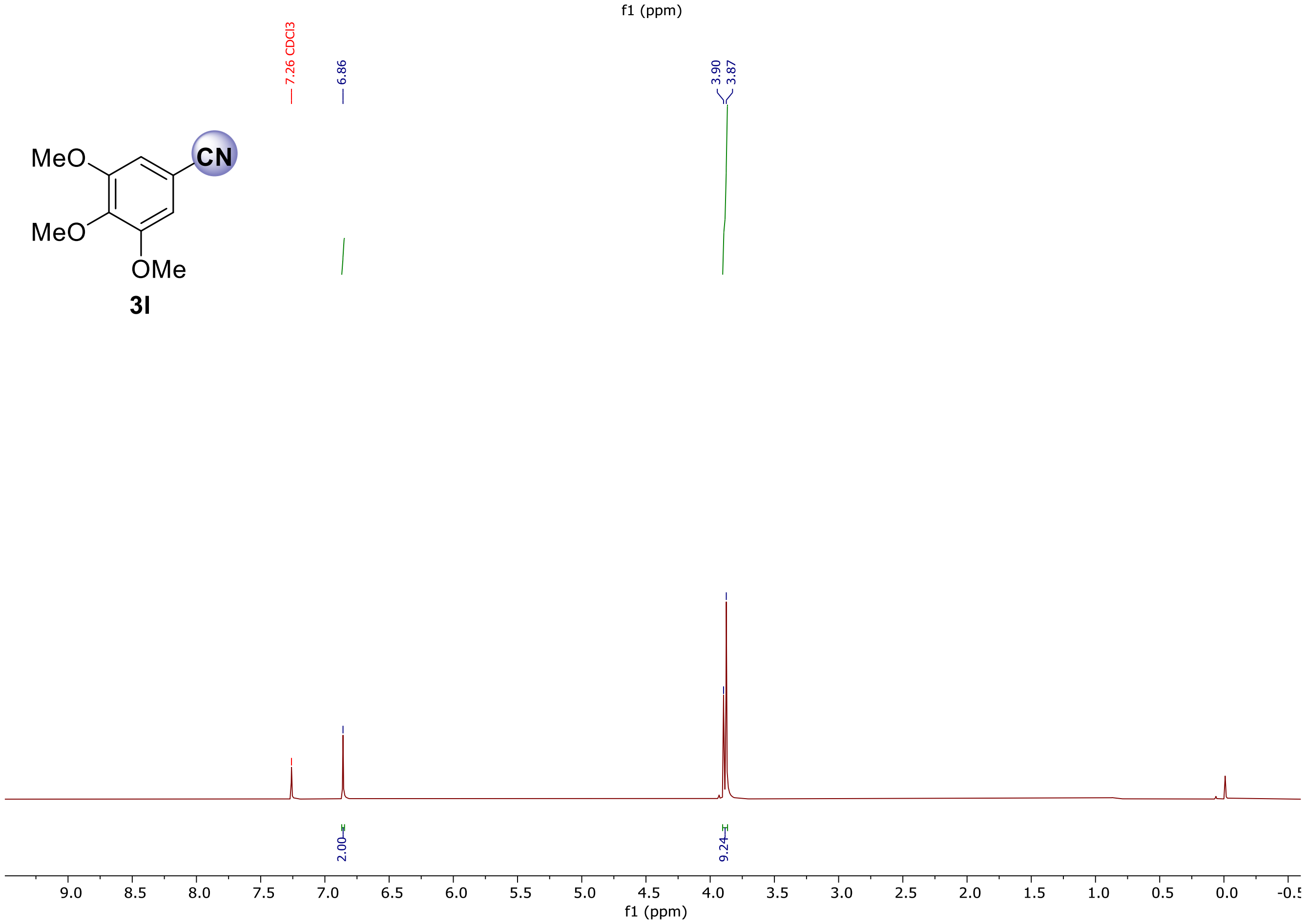
7.26 CDCl3
6.86
3.90
3.87
MeO
MeO
OMe
CN
3l
2.00
9.24
f1 (ppm)

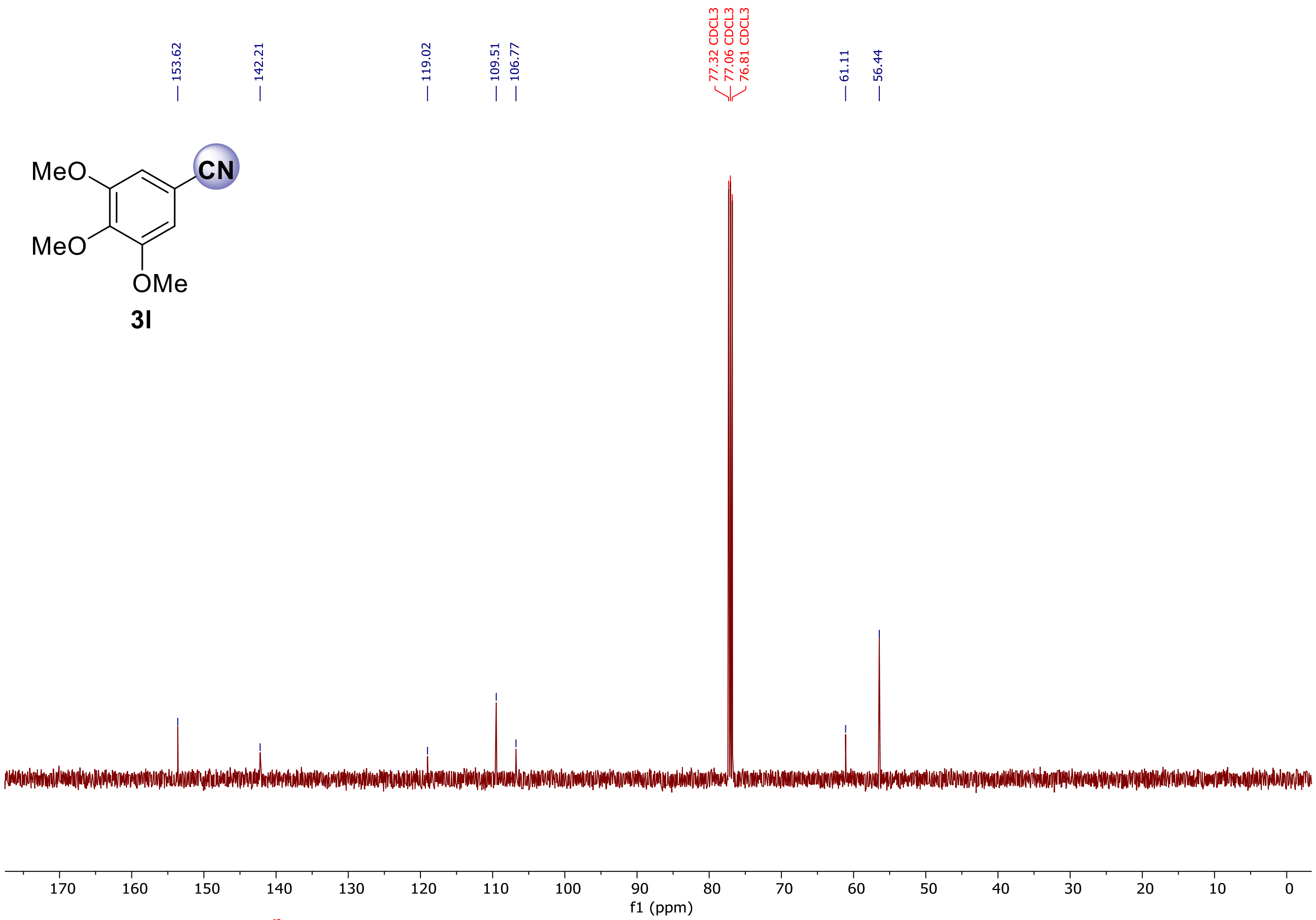
153.62
142.21
119.02
109.51
106.77
77.32 CDCL3
77.06 CDCL3
76.81 CDCL3
61.11
56.44
MeO
MeO
OMe
CN
f1 (ppm)

3l

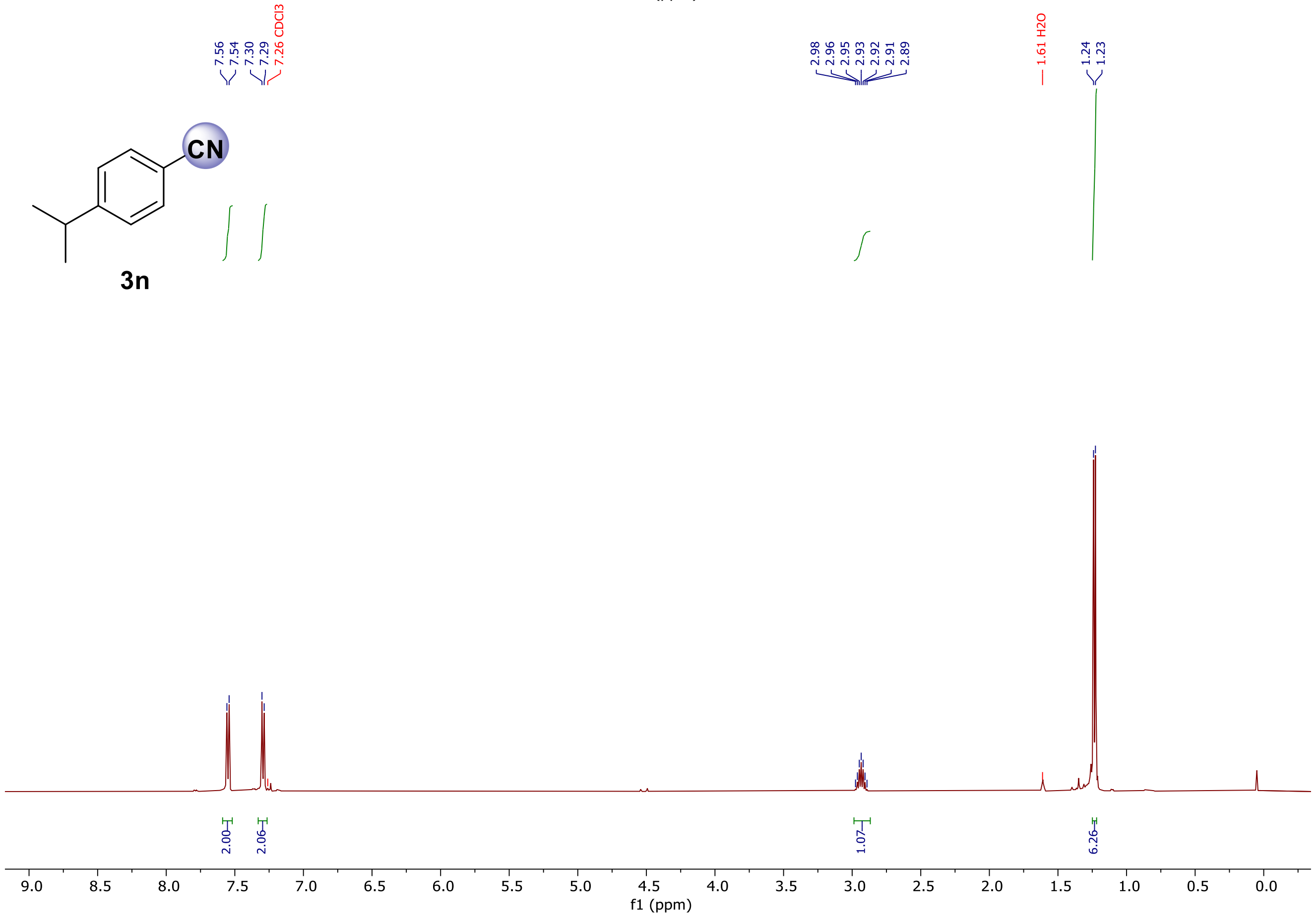
7.56
7.54
7.30
7.29
7.26 CDCl3
2.98
2.96
2.95
2.93
2.92
2.91
2.89
1.61 H2O
1.24
1.23
CN
2.00
2.06
1.07
6.26
f1 (ppm)

3n

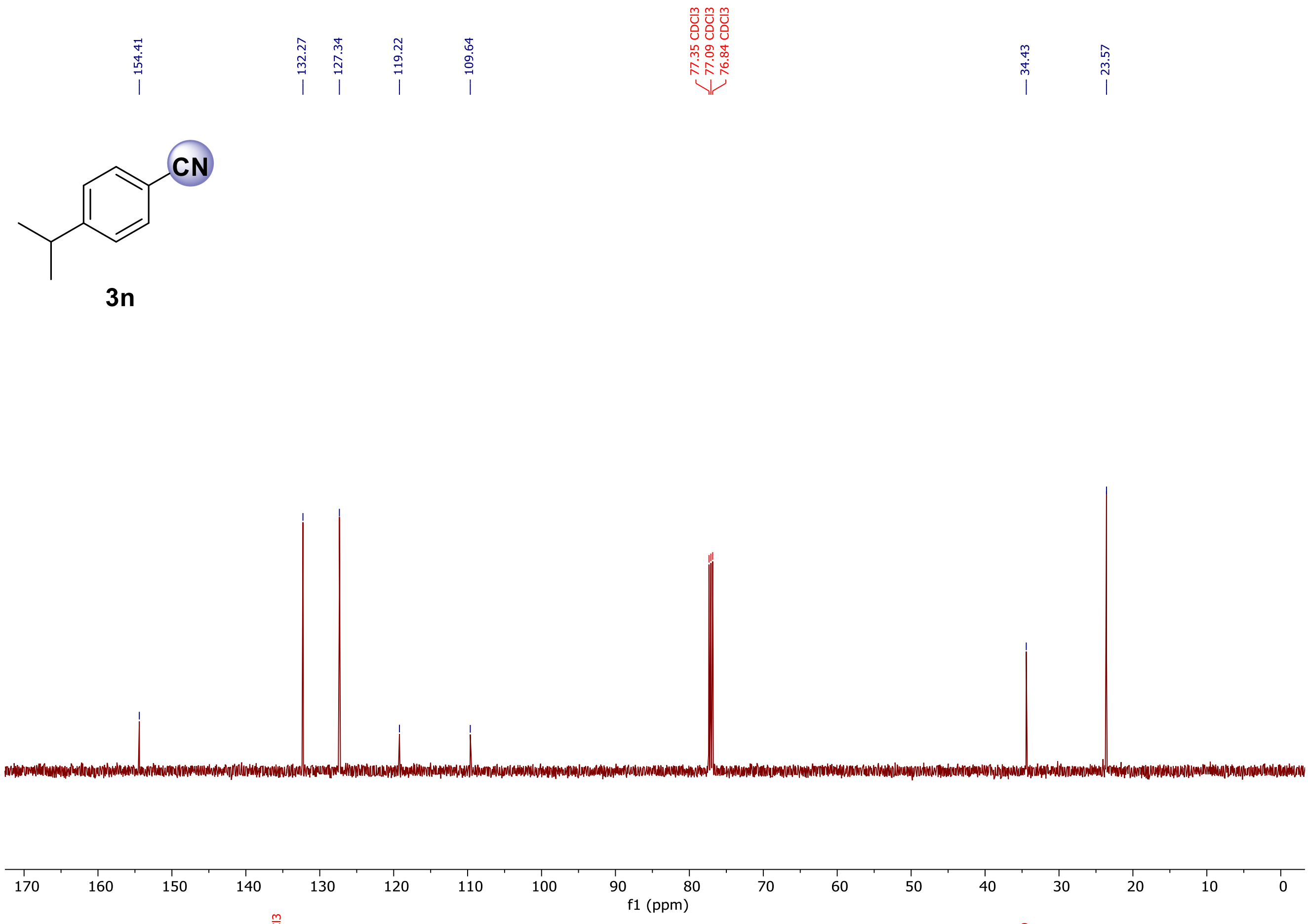
154.41
132.27
127.34
119.22
109.64
77.35 CDCl3
77.09 CDCl3
76.84 CDCl3
34.43
23.57
CN
3n
170 160 150 140 130 120 110 100 90 80 70 60 50 40 30 20 10 0
f1 (ppm)

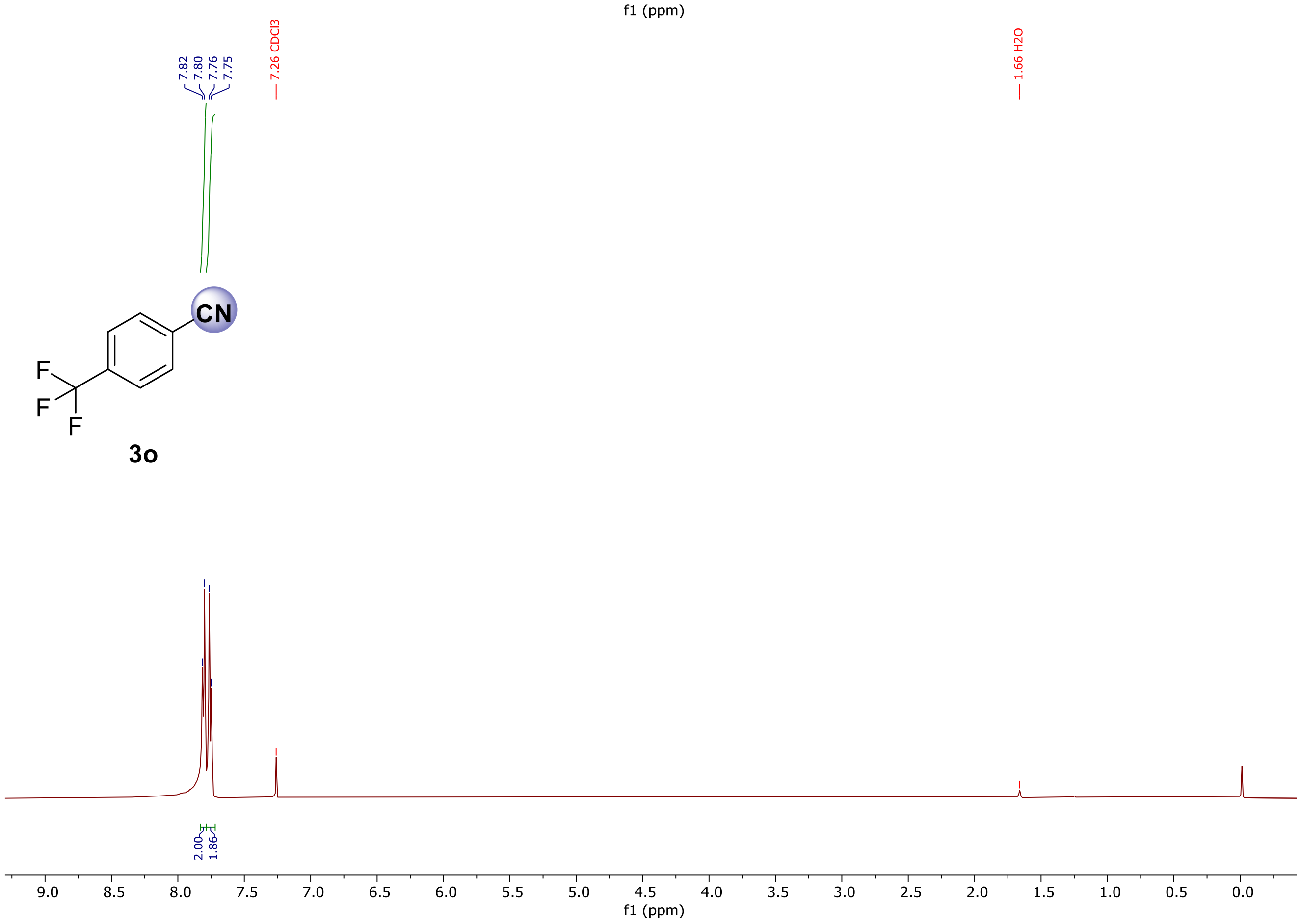
7.82
7.80
7.76
7.75
7.26 CDCl3
1.66 H2O
CN
F
F
F
3o
2.00
1.86
9.0 8.5 8.0 7.5 7.0 6.5 6.0 5.5 5.0 4.5 4.0 3.5 3.0 2.5 2.0 1.5 1.0 0.5 0.0
f1 (ppm)

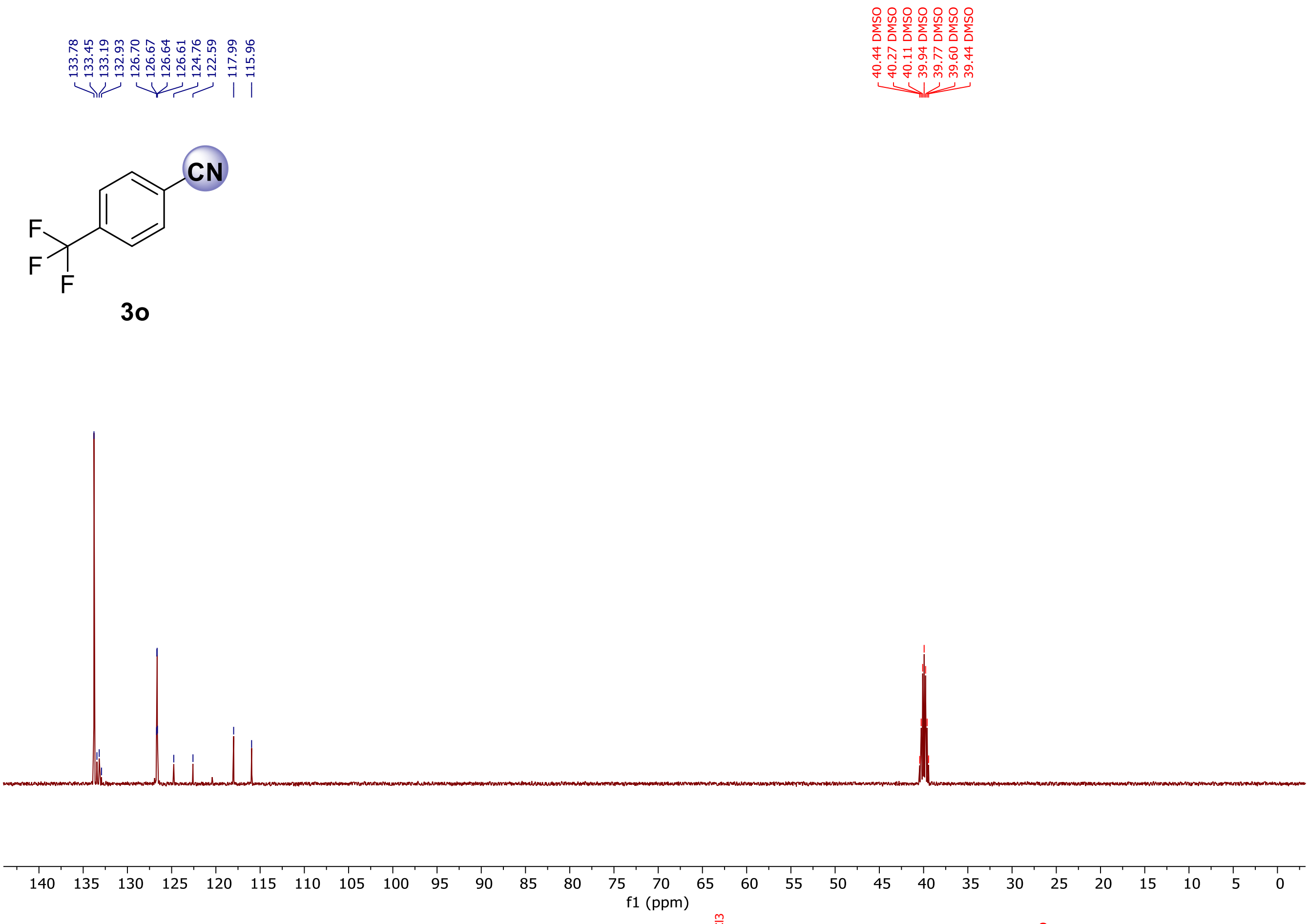
133.78
133.45
133.19
132.93
126.70
126.67
126.64
126.61
124.76
122.59
117.99
115.96
40.44 DMSO
40.27 DMSO
40.11 DMSO
39.94 DMSO
39.77 DMSO
39.60 DMSO
39.44 DMSO
CN
F
F
F
3o
140 135 130 125 120 115 110 105 100 95 90 85 80 75 70 65 60 55 50 45 40 35 30 25 20 15 10 5 0
f1 (ppm)

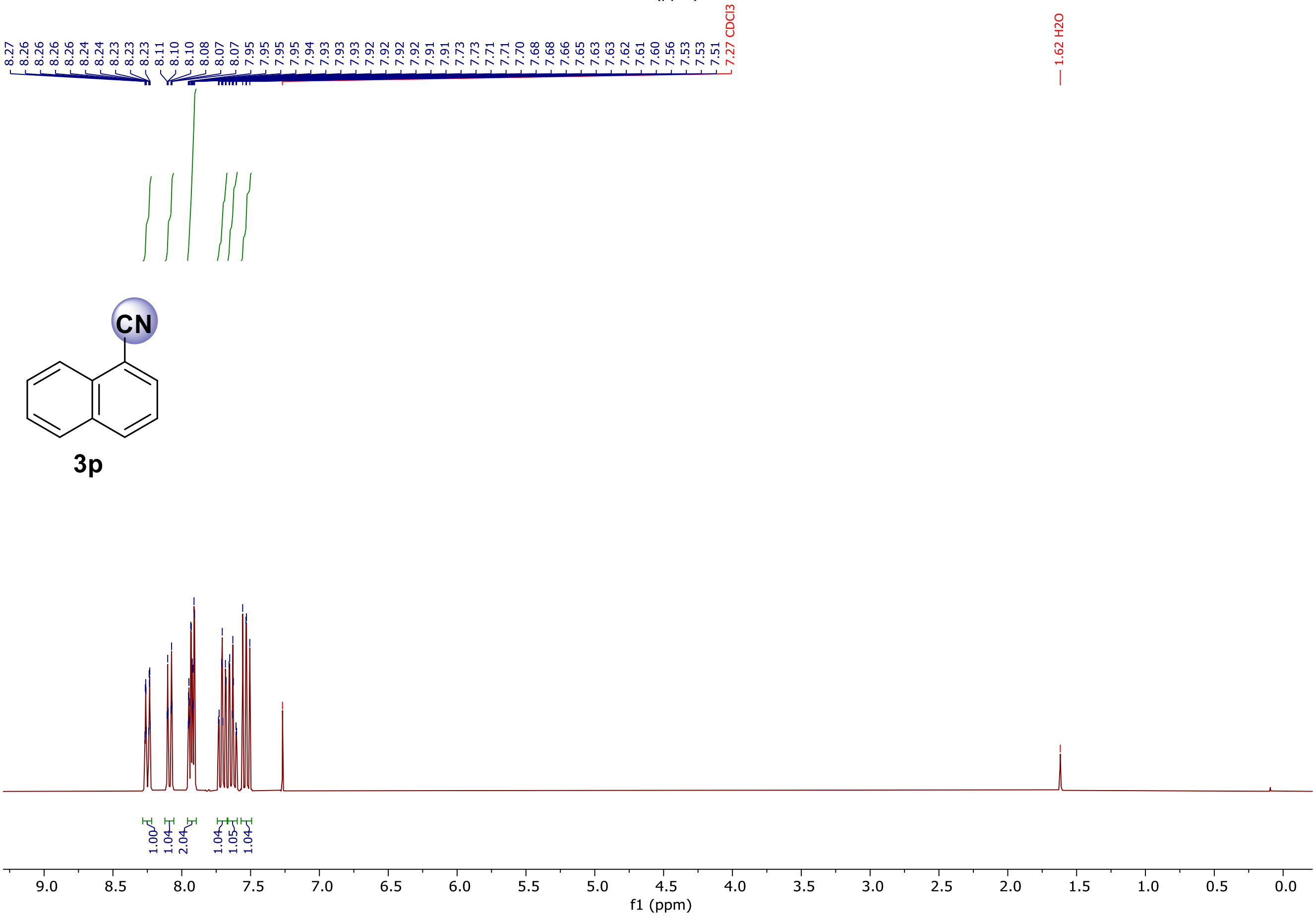
8.27
8.26
8.26
8.26
8.26
8.24
8.23
8.23
8.23
8.11
8.10
8.10
8.08
8.07
8.07
7.95
7.95
7.95
7.94
7.93
7.93
7.93
7.92
7.92
7.92
7.92
7.91
7.91
7.73
7.73
7.71
7.71
7.70
7.68
7.66
7.65
7.63
7.63
7.62
7.61
7.60
7.56
7.53
7.53
7.51
7.27 CDCl3
1.62 H2O
CN
3p
1.00
1.04
2.04
1.04
1.05
1.04
9.0 8.5 8.0 7.5 7.0 6.5 6.0 5.5 5.0 4.5 4.0 3.5 3.0 2.5 2.0 1.5 1.0 0.5 0.0
f1 (ppm)

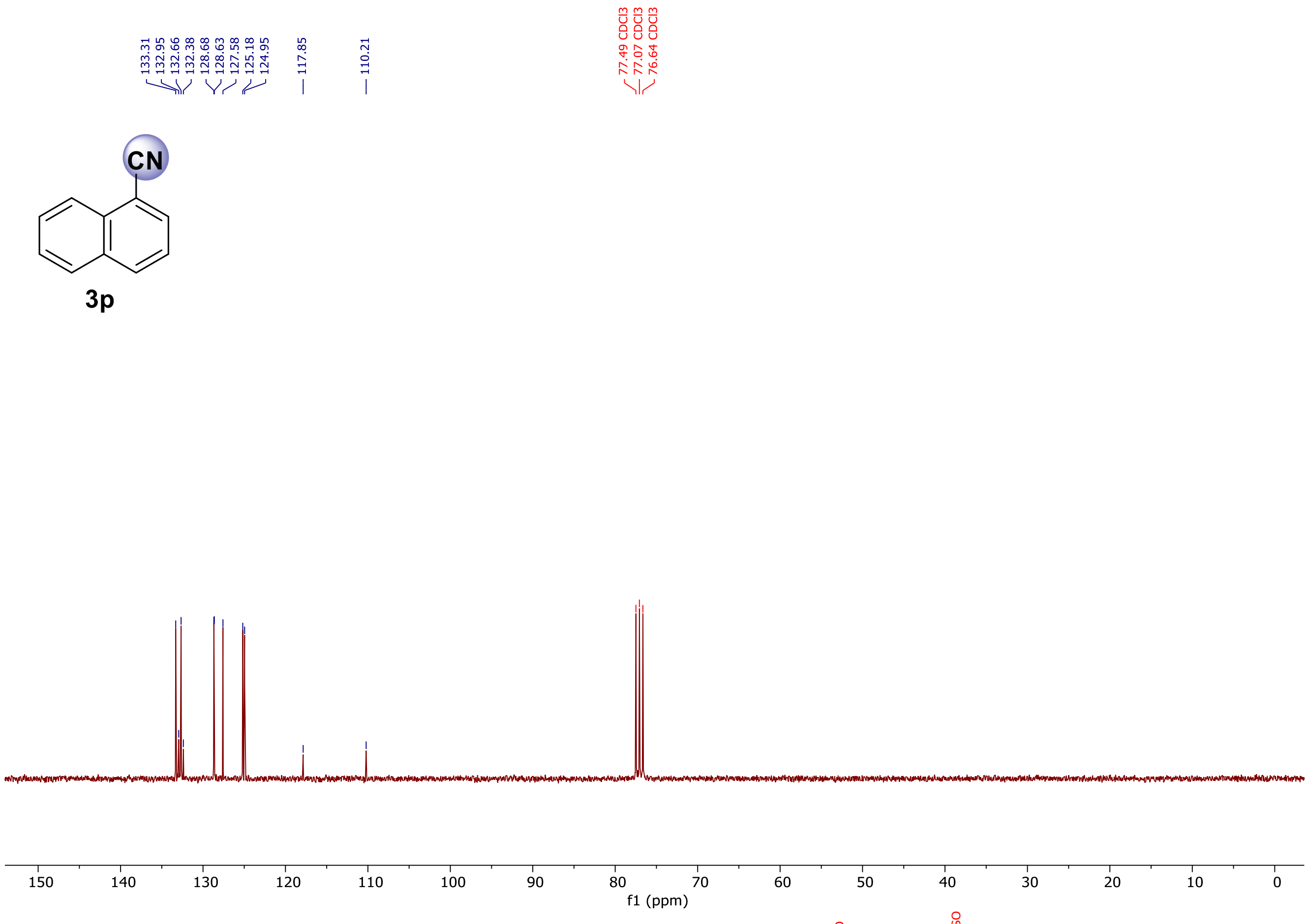
133.31
132.95
132.66
132.38
128.68
128.63
127.58
125.18
124.95
117.85
110.21
77.49 CDCl3
77.07 CDCl3
76.64 CDCl3
CN
3p
150 140 130 120 110 100 90 80 70 60 50 40 30 20 10 0
f1 (ppm)

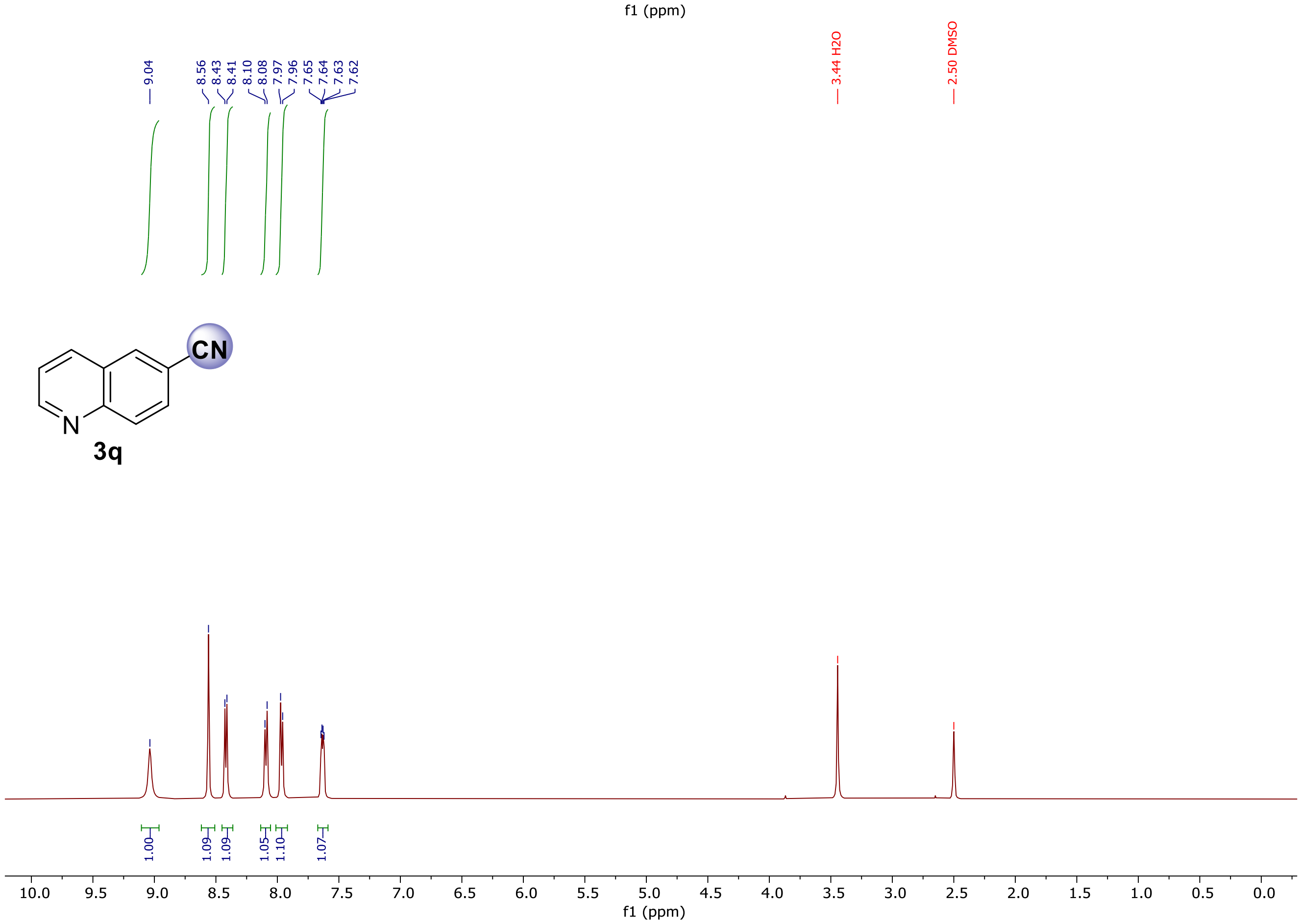
9.04
8.56
8.43
8.41
8.10
8.08
7.97
7.96
7.65
7.64
7.63
7.62
3.44 H2O
2.50 DMSO
CN
N
3q
1.00
1.09
1.09
1.05
1.10
1.07
10.0 9.5 9.0 8.5 8.0 7.5 7.0 6.5 6.0 5.5 5.0 4.5 4.0 3.5 3.0 2.5 2.0 1.5 1.0 0.5 0.0
f1 (ppm)

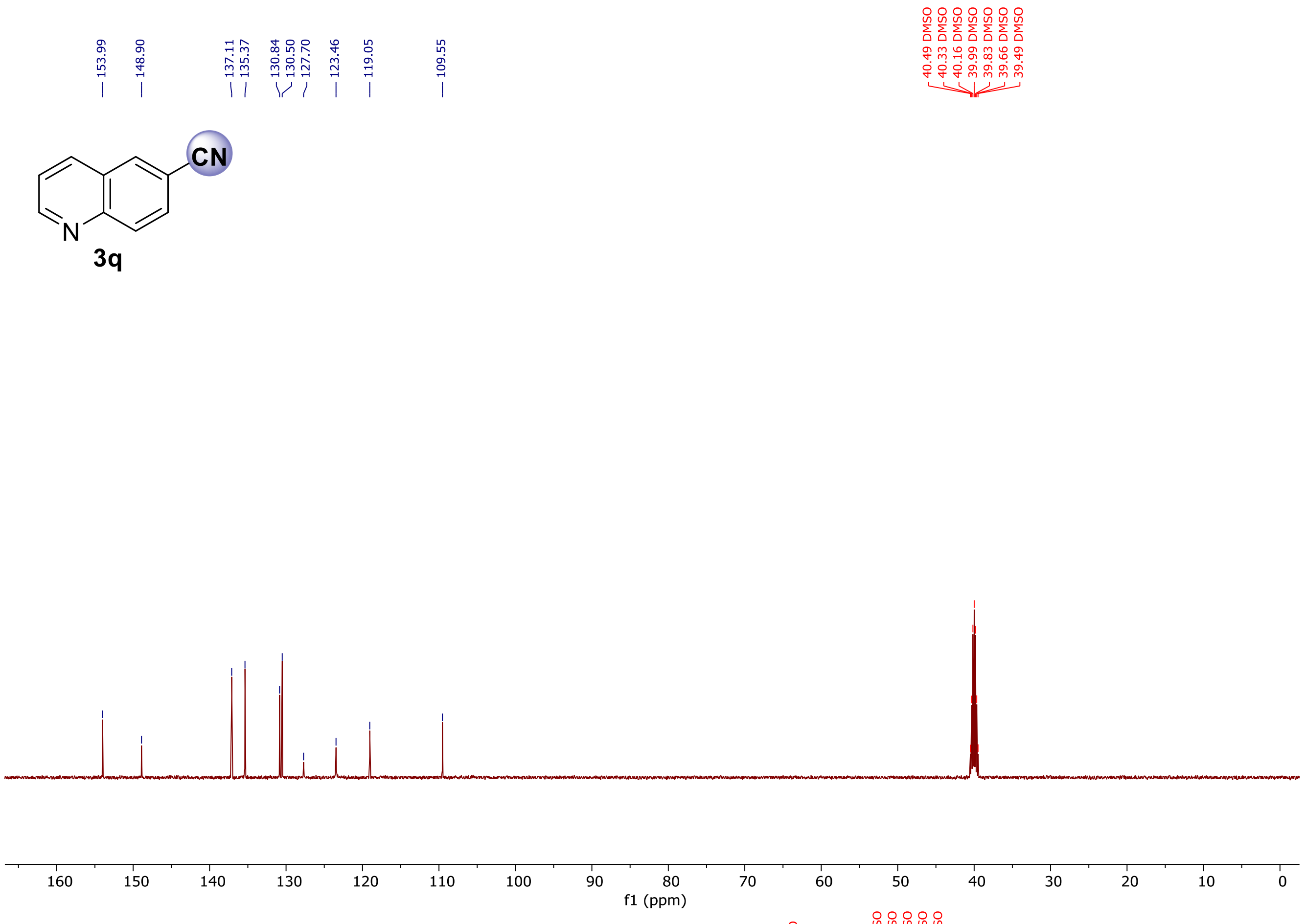
153.99
148.90
137.11
135.37
130.84
130.50
127.70
123.46
119.05
109.55
40.49 DMSO
40.33 DMSO
40.16 DMSO
39.99 DMSO
39.83 DMSO
39.66 DMSO
39.49 DMSO
CN
N
3q
160 150 140 130 120 110 100 90 80 70 60 50 40 30 20 10 0
f1 (ppm)

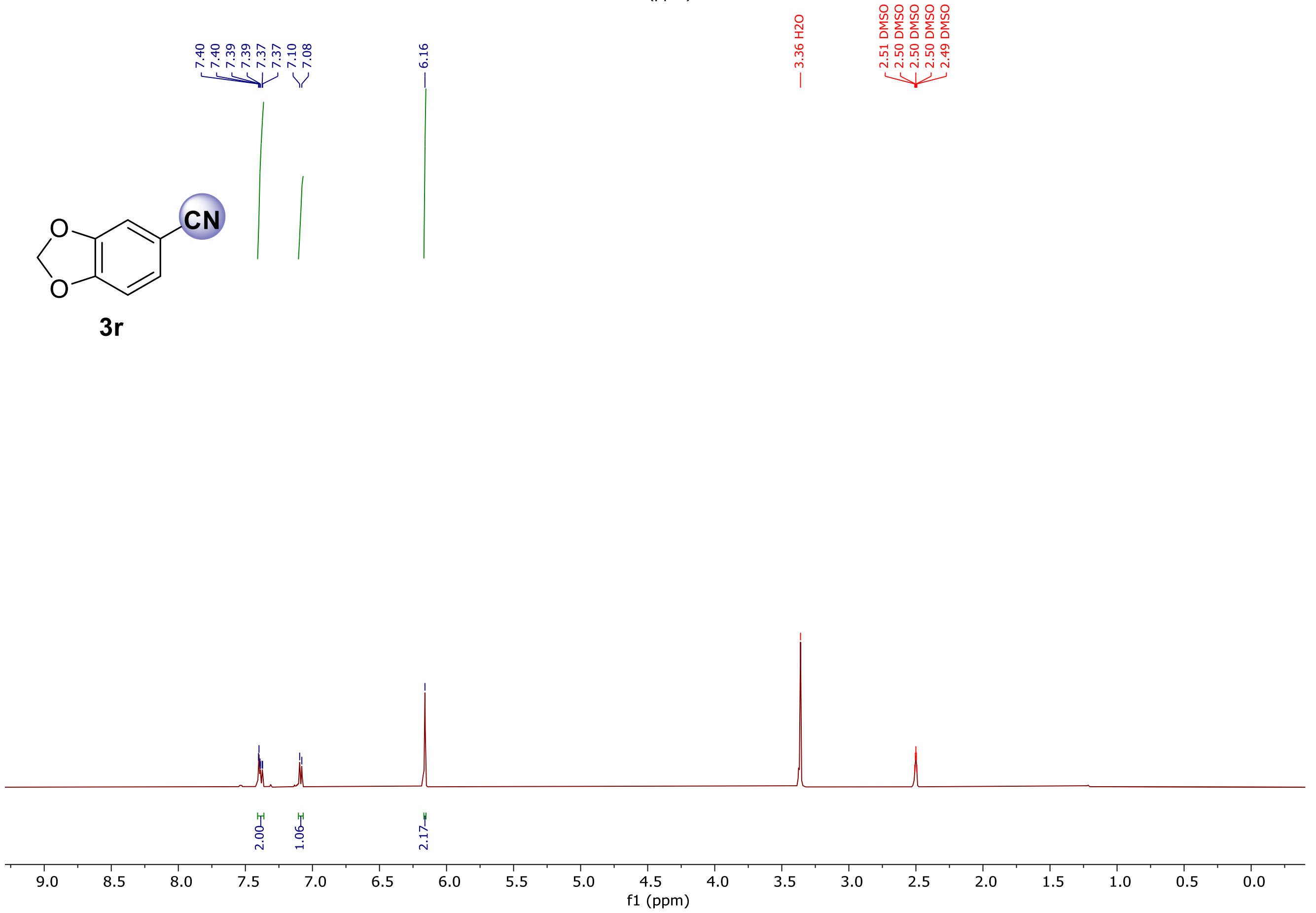
7.40
7.40
7.39
7.39
7.37
7.37
7.10
7.08
6.16
3.36 H2O
2.51 DMSO
2.50 DMSO
2.50 DMSO
2.50 DMSO
2.49 DMSO
O
O
CN
3r
2.00
1.06
2.17
9.0 8.5 8.0 7.5 7.0 6.5 6.0 5.5 5.0 4.5 4.0 3.5 3.0 2.5 2.0 1.5 1.0 0.5 0.0
f1 (ppm)

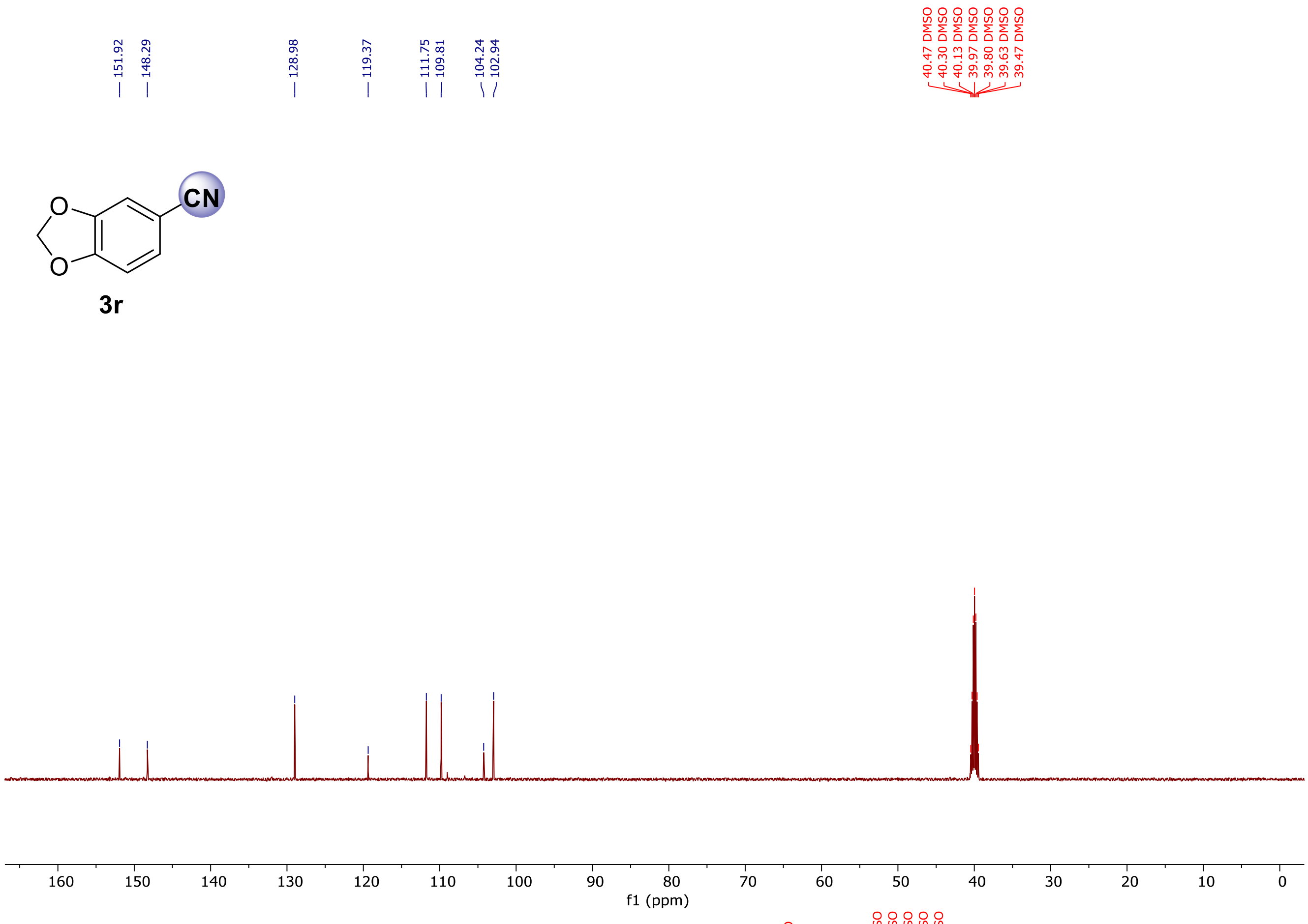
CN
3r
151.92
148.29
128.98
119.37
111.75
109.81
104.24
102.94
40.47 DMSO
40.30 DMSO
40.13 DMSO
39.97 DMSO
39.80 DMSO
39.63 DMSO
39.47 DMSO
f1 (ppm)

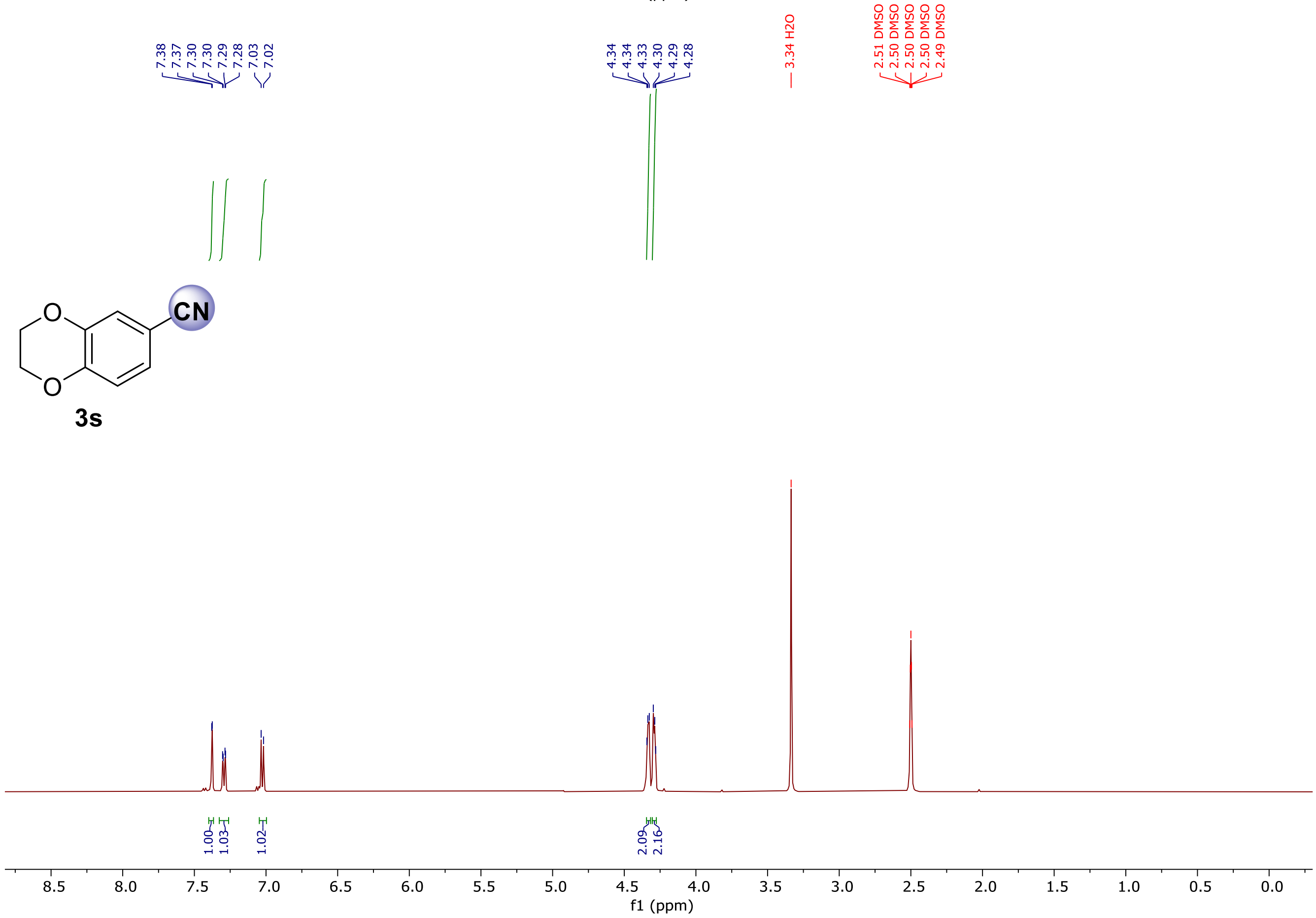
CN
3s
7.38
7.37
7.30
7.30
7.29
7.28
7.03
7.02
4.34
4.34
4.33
4.30
4.29
4.28
3.34 H2O
2.51 DMSO
2.50 DMSO
2.50 DMSO
2.50 DMSO
2.49 DMSO
1.00
1.03
1.02
2.09
2.16
f1 (ppm)

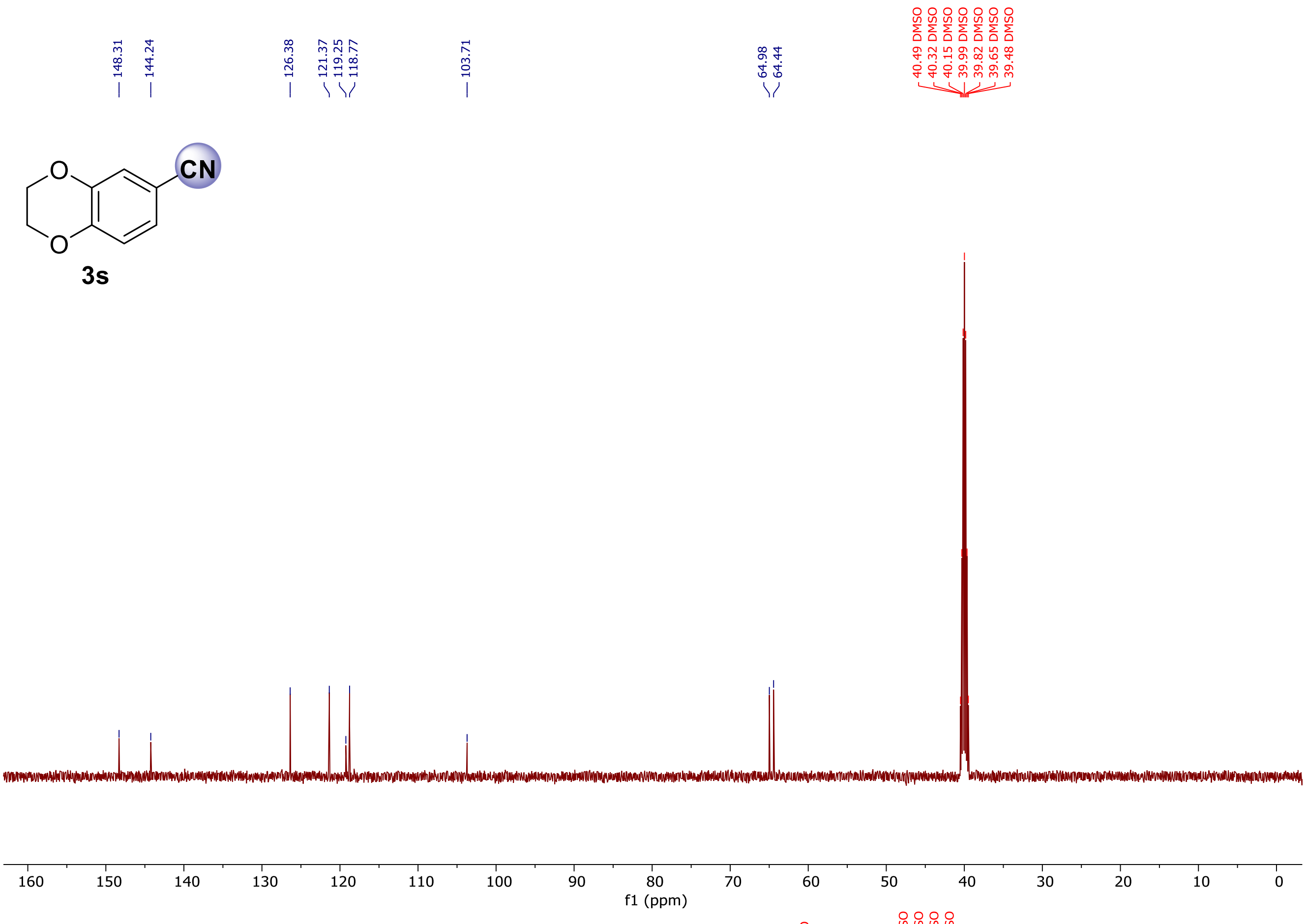
148.31
144.24
126.38
121.37
119.25
118.77
103.71
64.98
64.44
40.49 DMSO
40.32 DMSO
40.15 DMSO
39.99 DMSO
39.82 DMSO
39.65 DMSO
39.48 DMSO
CN
3s
f1 (ppm)

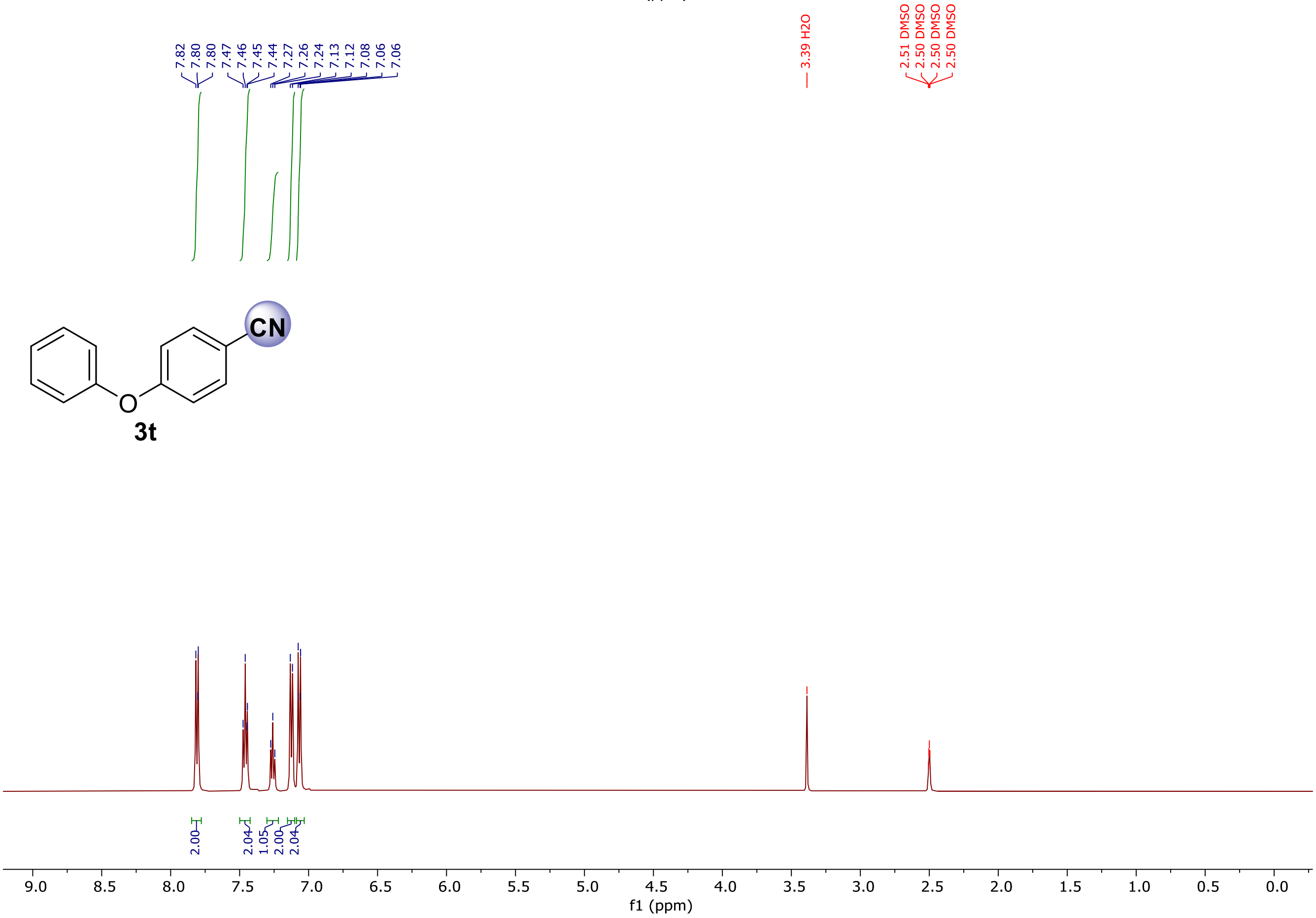
7.82
7.80
7.80
7.47
7.46
7.45
7.44
7.27
7.26
7.24
7.13
7.12
7.08
7.06
7.06
3.39 H2O
2.51 DMSO
2.50 DMSO
2.50 DMSO
2.50 DMSO
CN
3t
2.00
2.04
1.05
2.00
2.04
f1 (ppm)

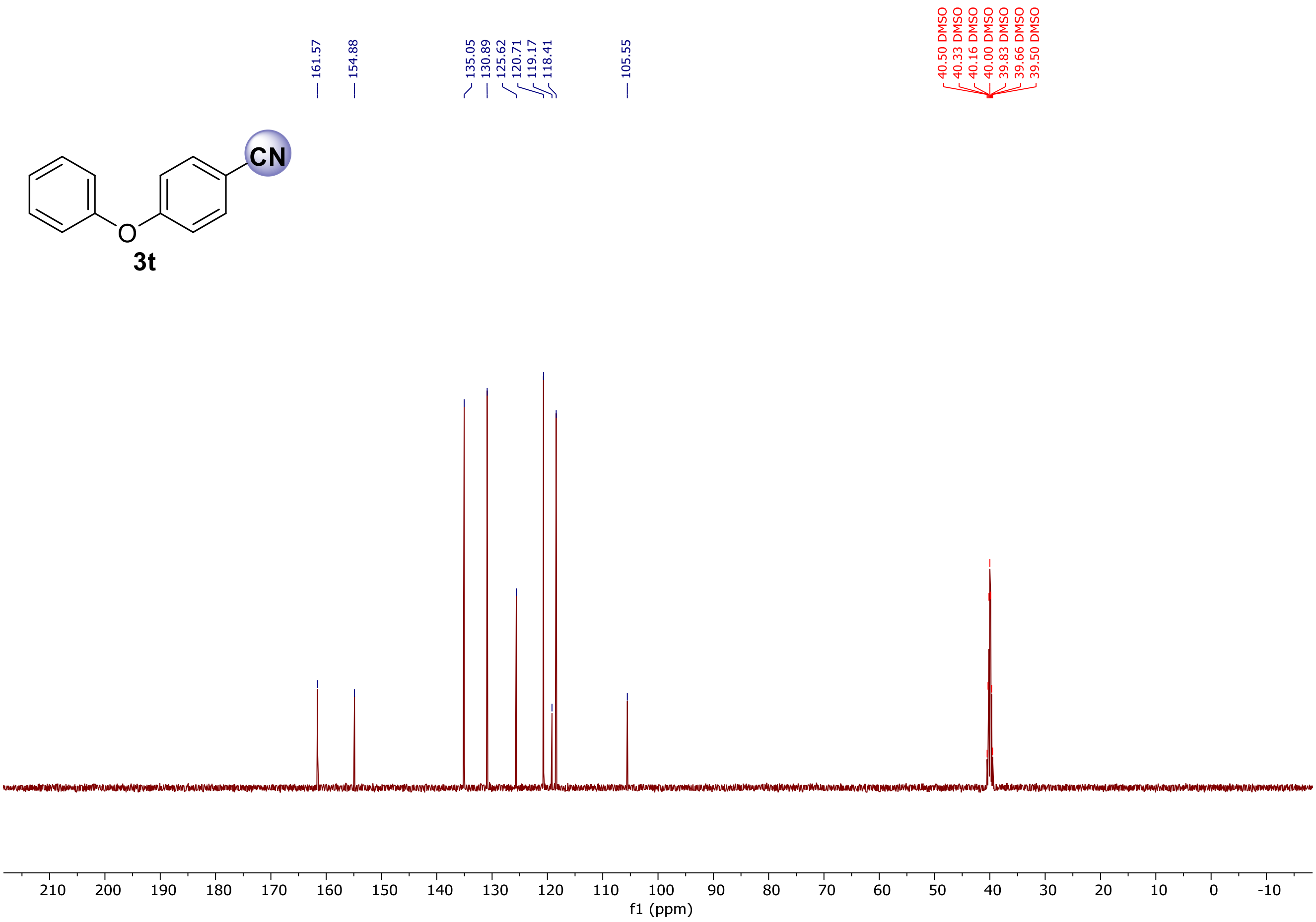
161.57
154.88
135.05
130.89
125.62
120.71
119.17
118.41
105.55
40.50 DMSO
40.33 DMSO
40.16 DMSO
40.00 DMSO
39.83 DMSO
39.66 DMSO
39.50 DMSO
CN
O
3t
210 200 190 180 170 160 150 140 130 120 110 100 90 80 70 60 50 40 30 20 10 0 -10
f1 (ppm)

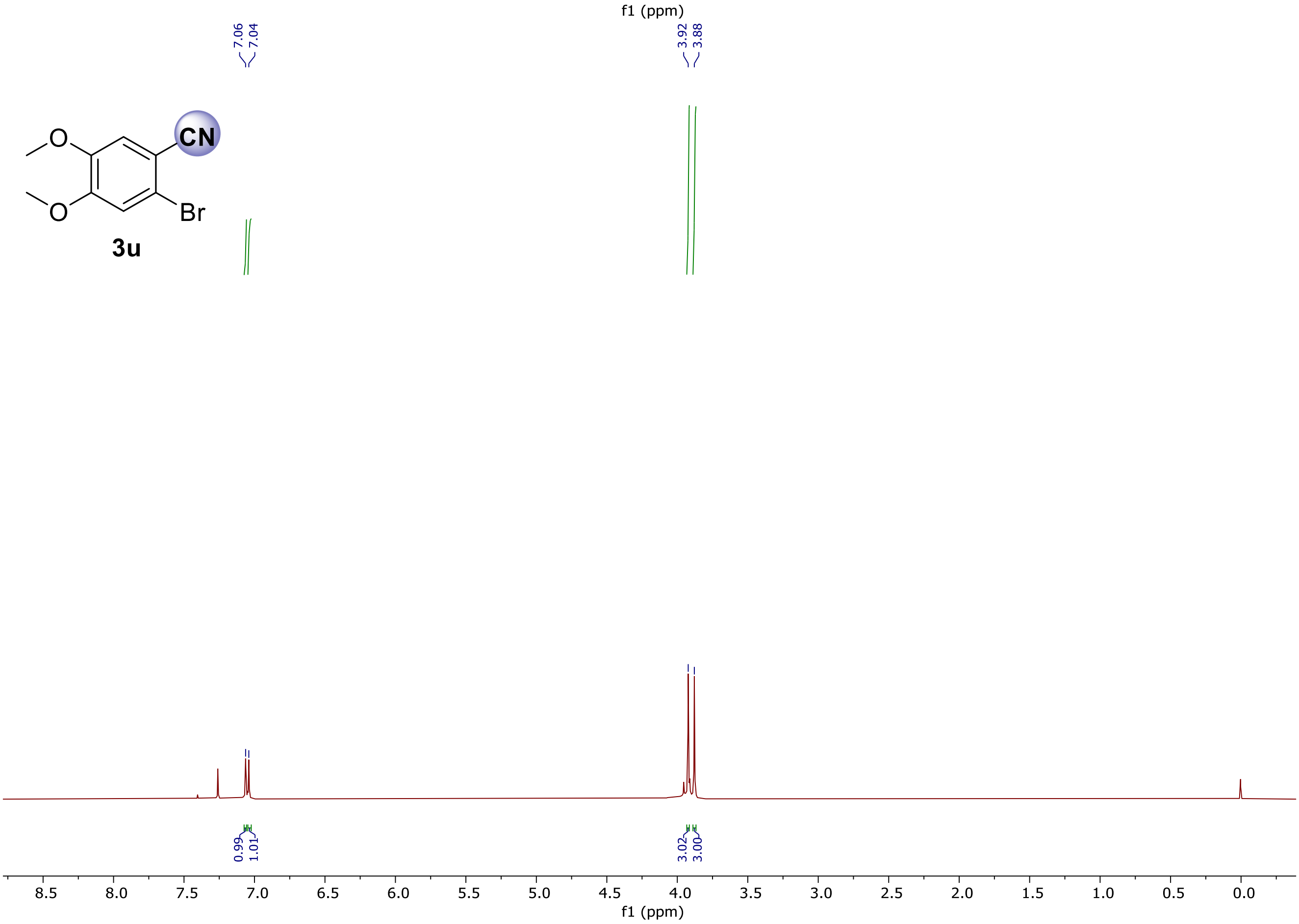
7.06
7.04
3.92
3.88
O
O
CN
Br
3u
0.99
1.01
3.02
3.00
8.5 8.0 7.5 7.0 6.5 6.0 5.5 5.0 4.5 4.0 3.5 3.0 2.5 2.0 1.5 1.0 0.5 0.0
f1 (ppm)

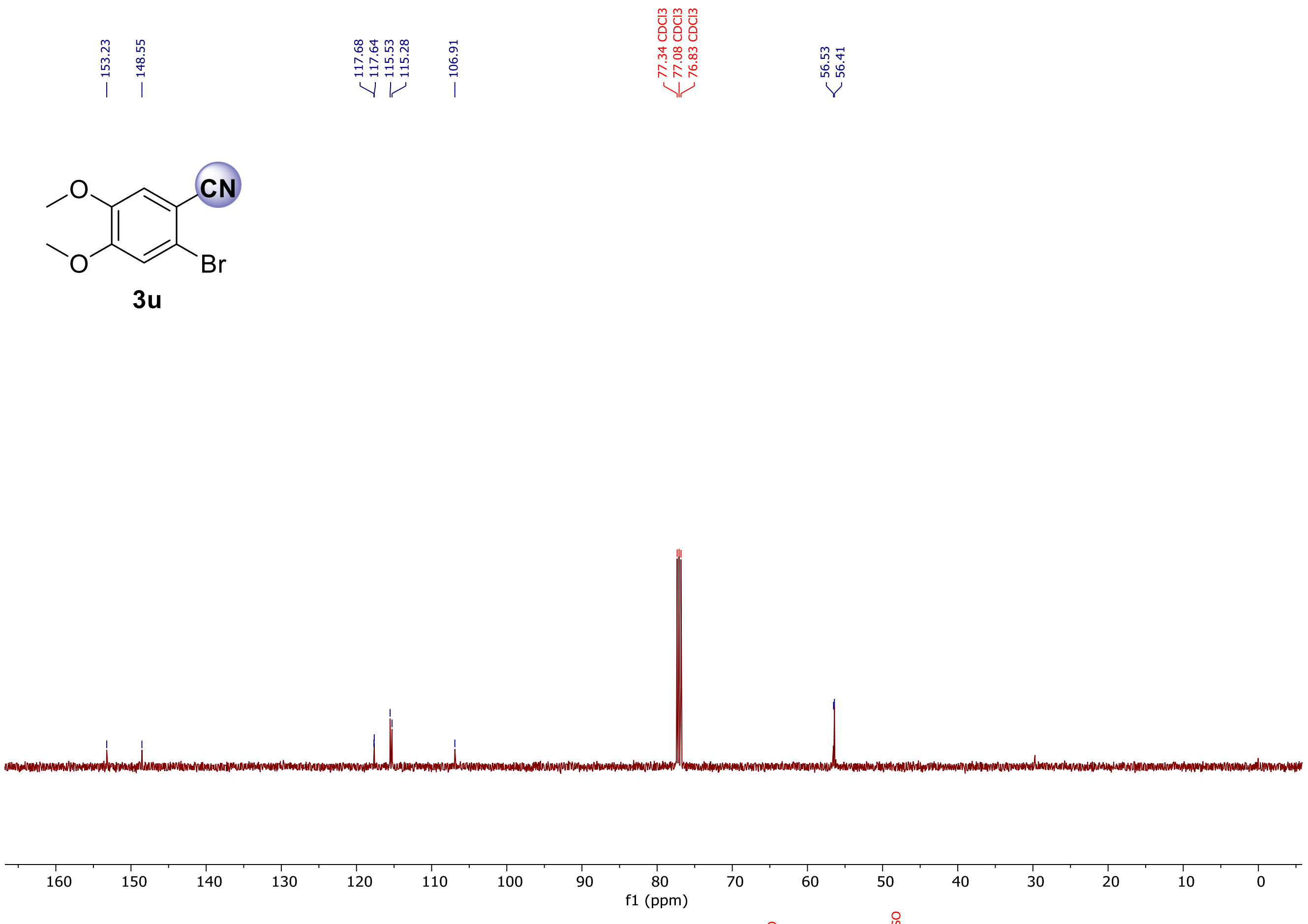
153.23
148.55
117.68
117.64
115.53
115.28
106.91
77.34 CDCl3
77.08 CDCl3
76.83 CDCl3
56.53
56.41
CN
O
O
Br
3u
f1 (ppm)

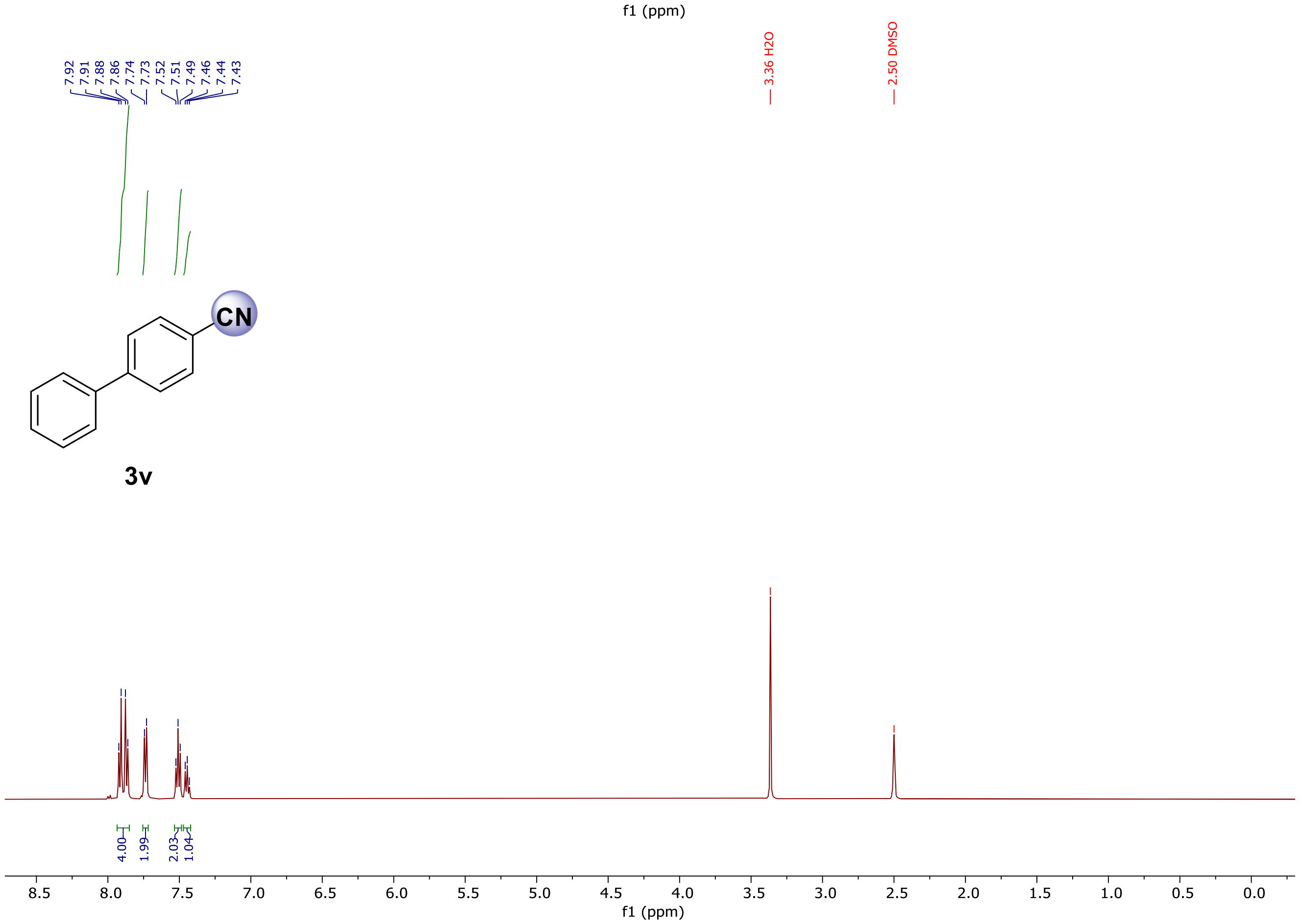
7.92
7.91
7.88
7.86
7.74
7.73
7.52
7.51
7.49
7.46
7.44
7.43
3.36 H2O
2.50 DMSO
CN
3v
4.00
1.99
2.03
1.04
f1 (ppm)

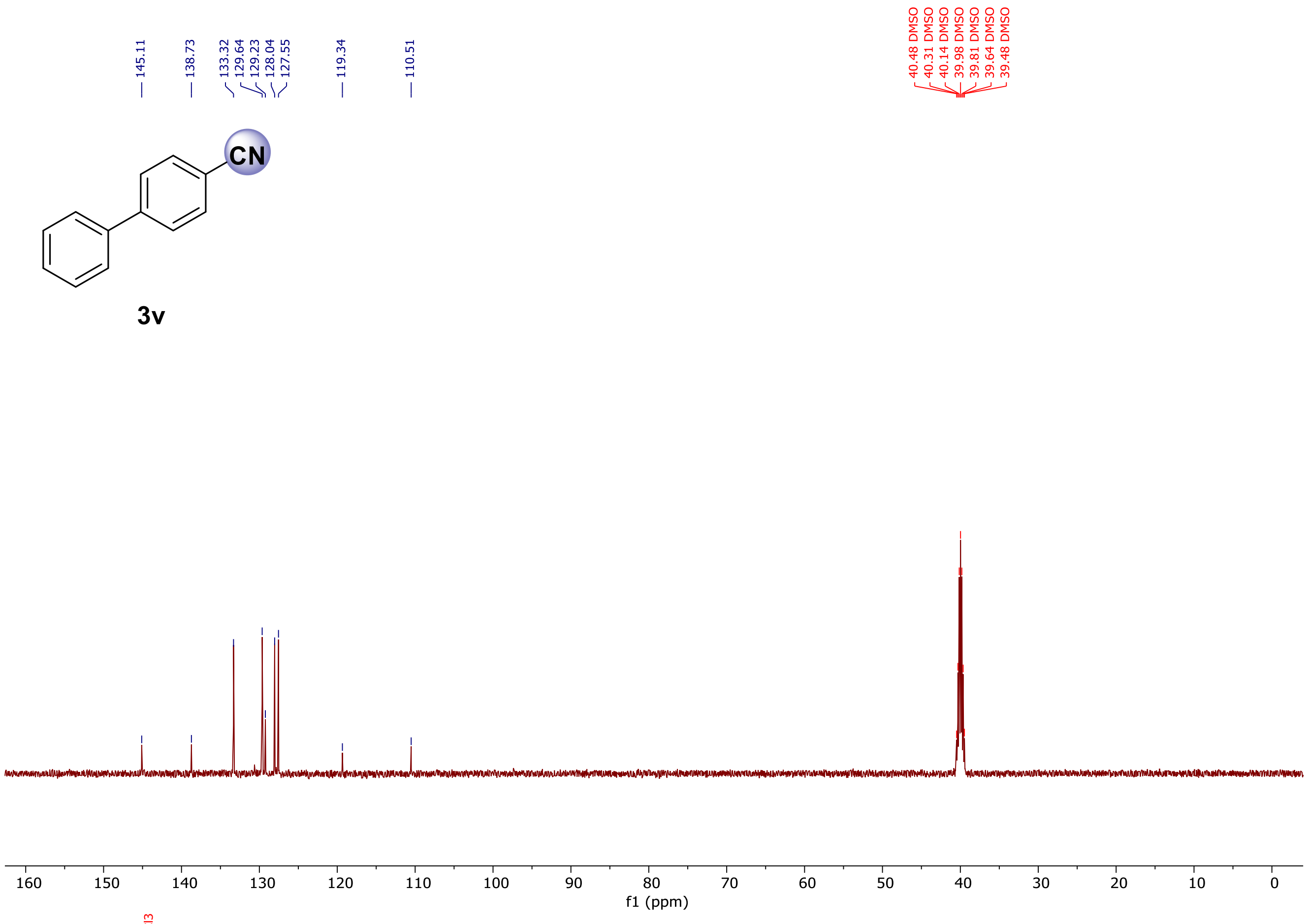
145.11
138.73
133.32
129.64
129.23
128.04
127.55
119.34
110.51
40.48 DMSO
40.31 DMSO
40.14 DMSO
39.98 DMSO
39.81 DMSO
39.64 DMSO
39.48 DMSO
CN
3v
f1 (ppm)

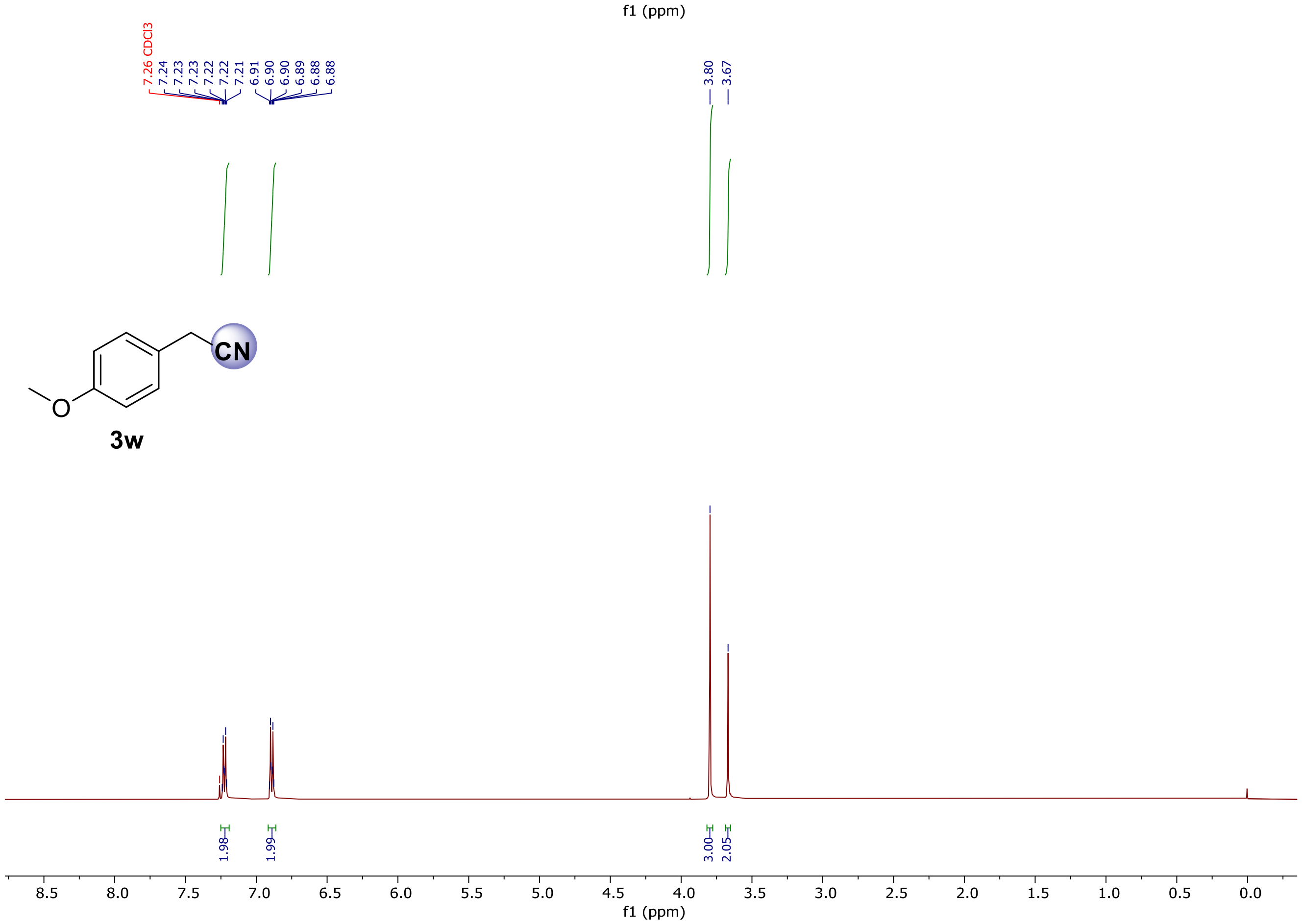
7.26 CDCl3
7.24
7.23
7.23
7.22
7.22
7.21
6.91
6.90
6.90
6.89
6.88
6.88
3.80
3.67
CN
O
3w
1.98
1.99
3.00
2.05
f1 (ppm)

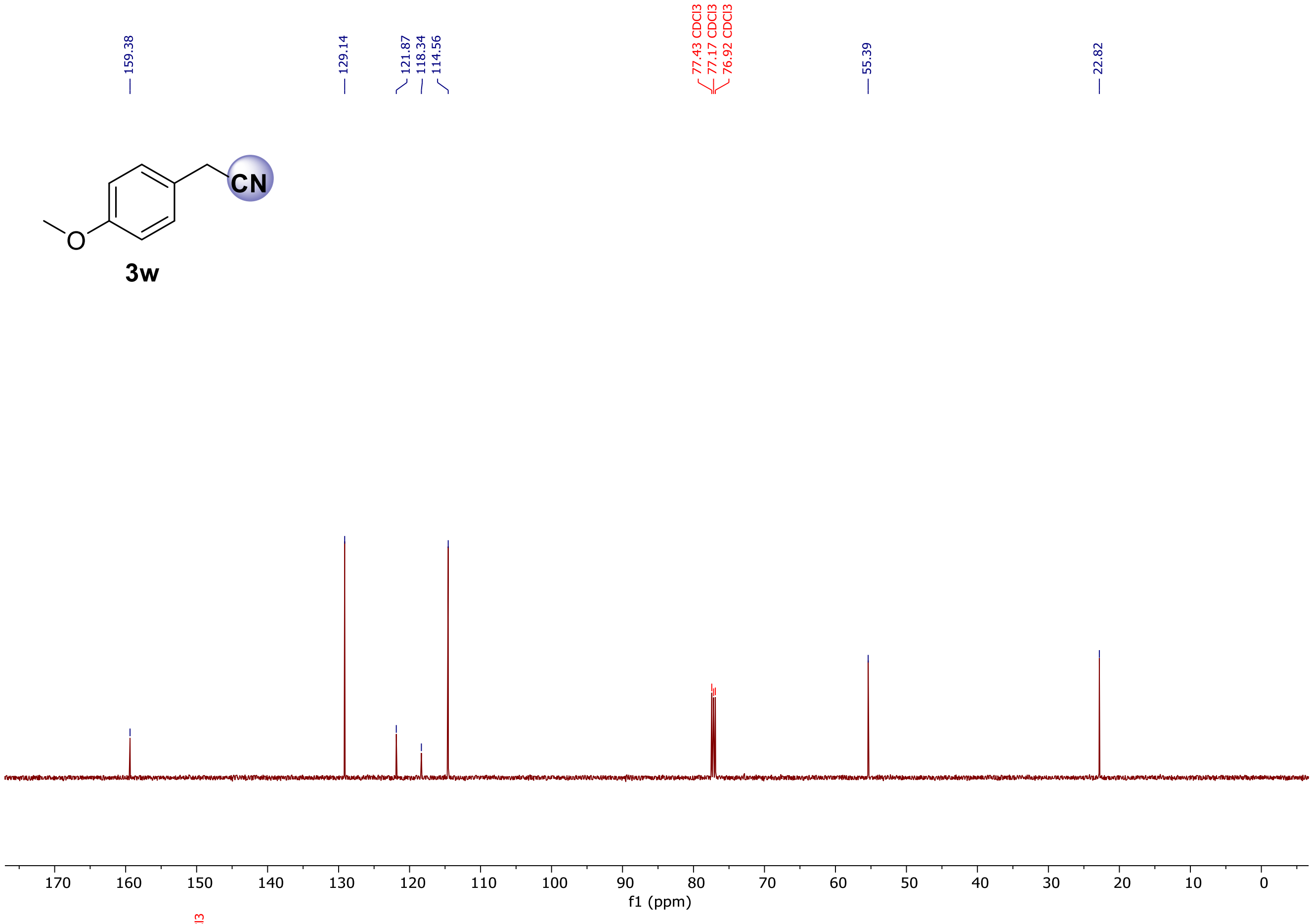
159.38
129.14
121.87
118.34
114.56
77.43 CDCl3
77.17 CDCl3
76.92 CDCl3
55.39
22.82
CN
O
3w
170 160 150 140 130 120 110 100 90 80 70 60 50 40 30 20 10 0
f1 (ppm)

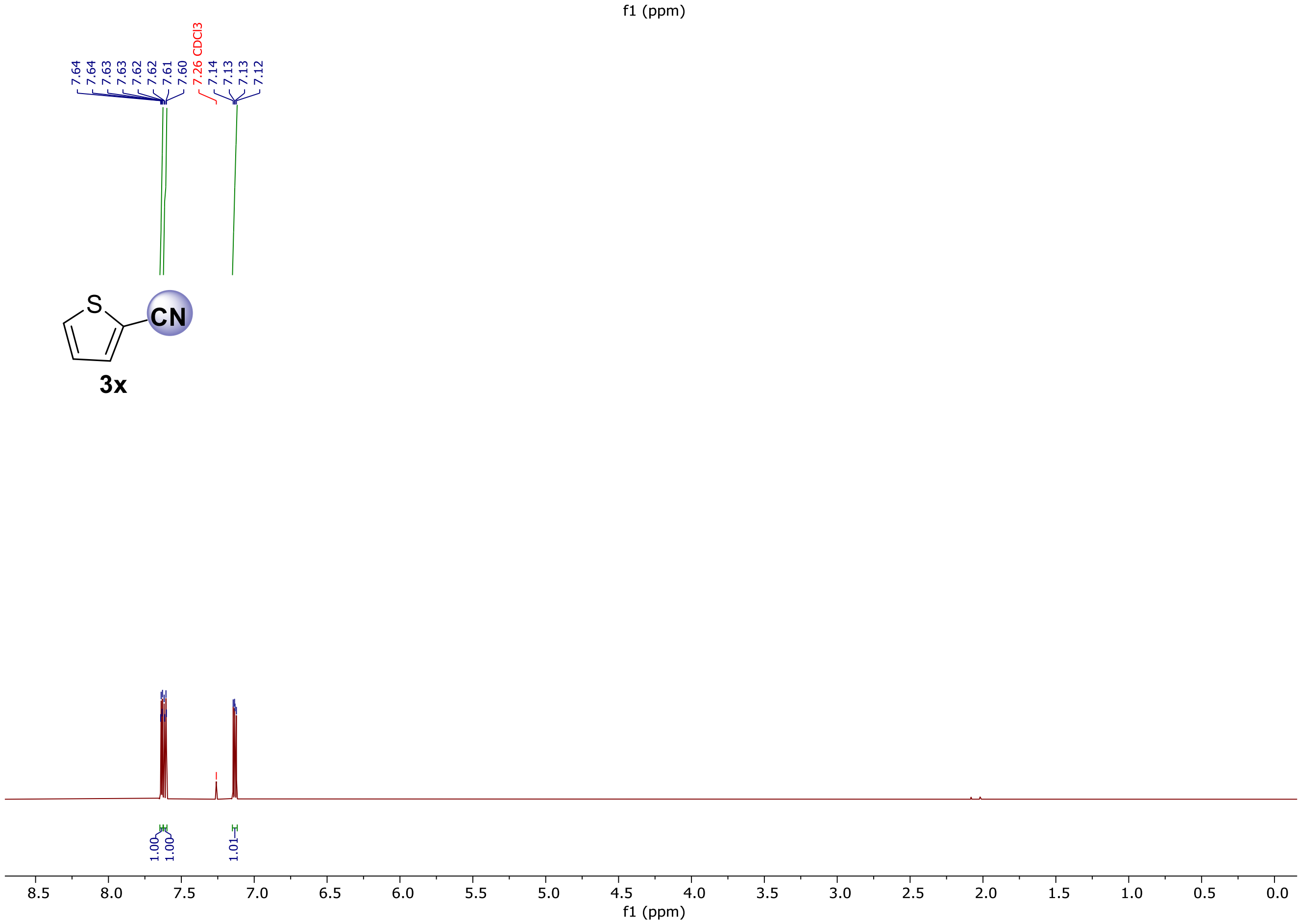
7.64
7.64
7.63
7.63
7.62
7.62
7.61
7.60
7.26 CDCl3
7.14
7.13
7.13
7.12
S
CN
3x
1.00
1.00
1.01
8.5 8.0 7.5 7.0 6.5 6.0 5.5 5.0 4.5 4.0 3.5 3.0 2.5 2.0 1.5 1.0 0.5 0.0
f1 (ppm)

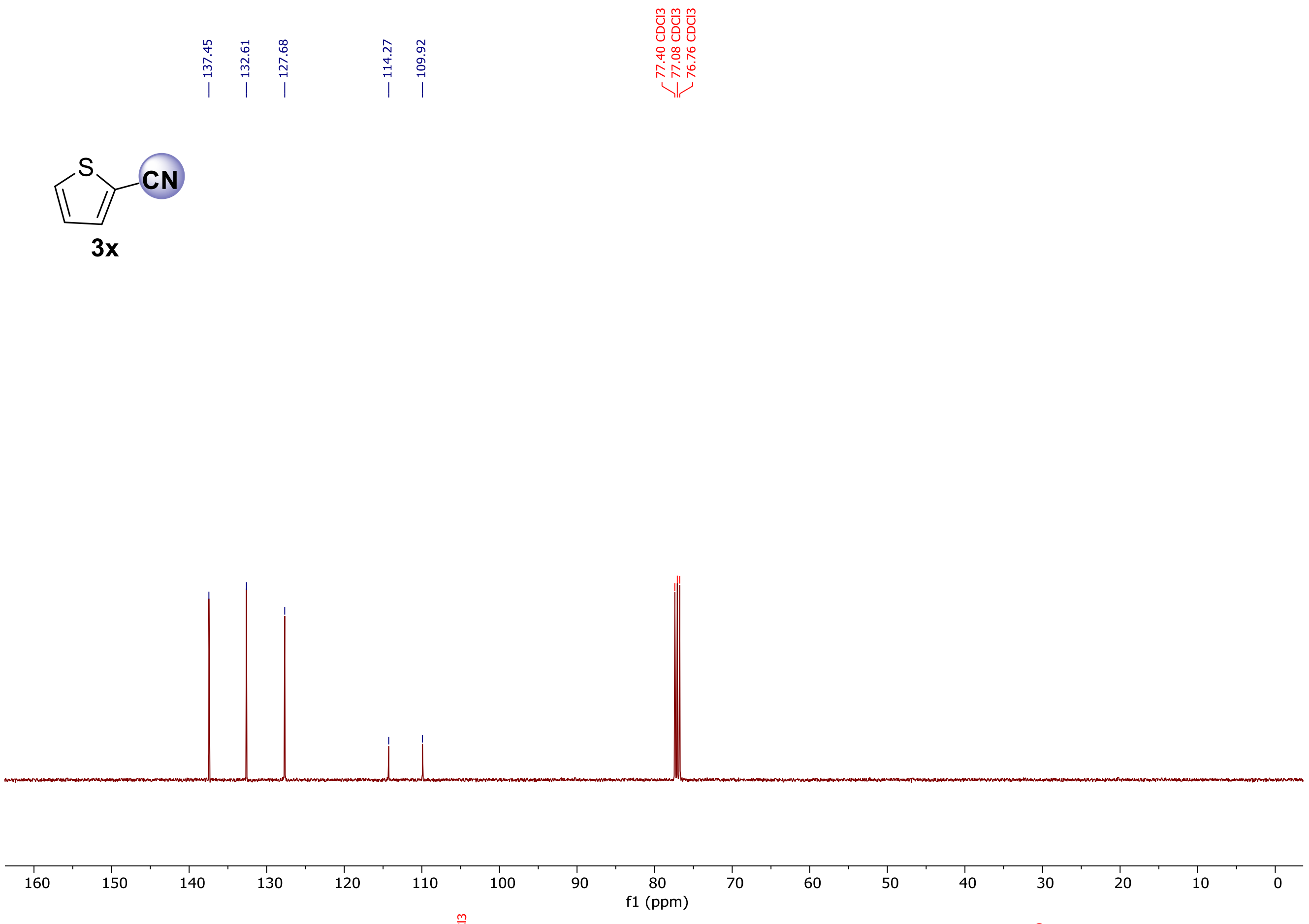
137.45
132.61
127.68
114.27
109.92
77.40 CDCl3
77.08 CDCl3
76.76 CDCl3
S
CN
3x
160 150 140 130 120 110 100 90 80 70 60 50 40 30 20 10 0
f1 (ppm)

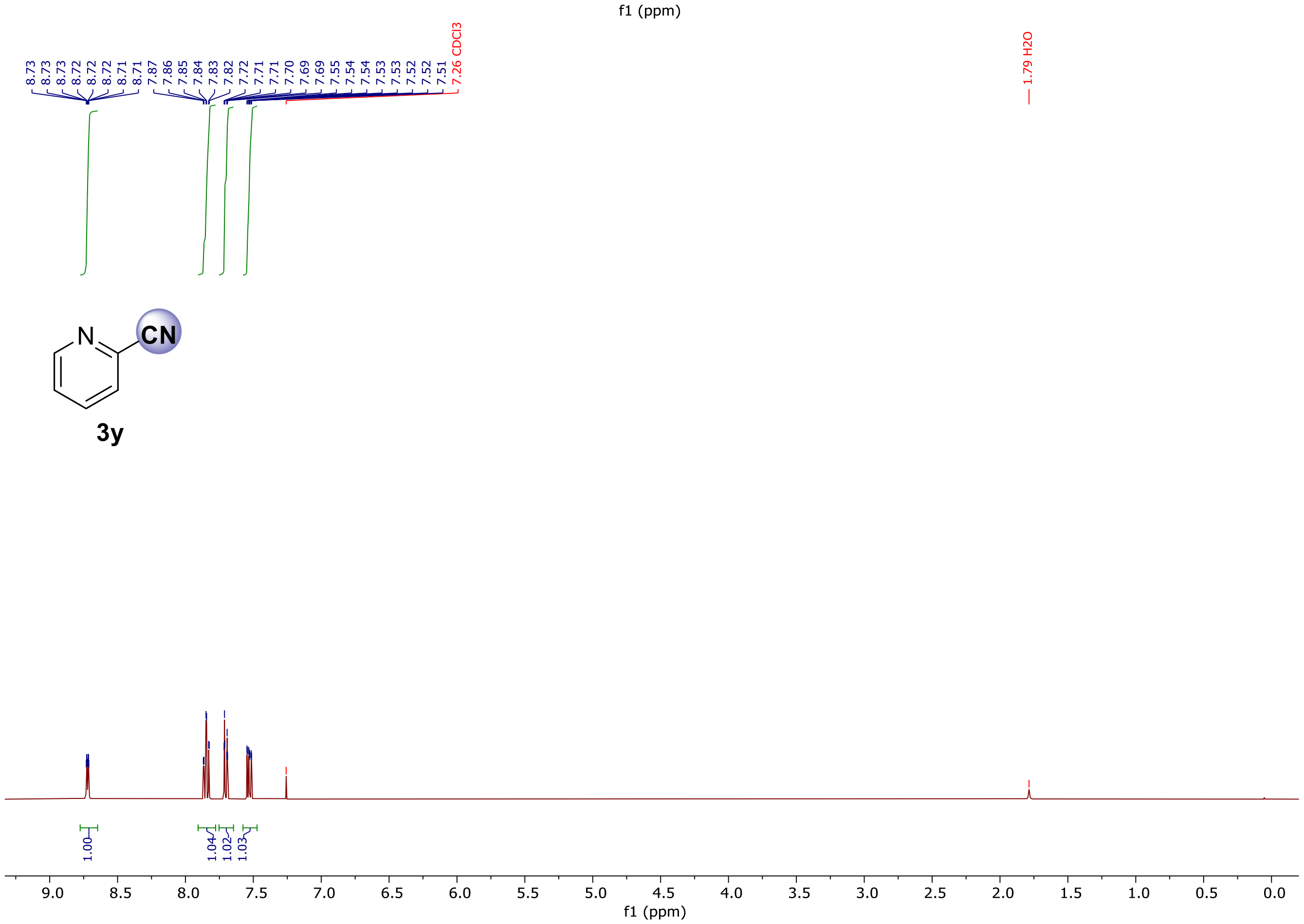
8.73
8.73
8.73
8.72
8.72
8.72
8.71
8.71
7.87
7.86
7.85
7.84
7.83
7.82
7.72
7.71
7.71
7.70
7.69
7.55
7.54
7.54
7.53
7.53
7.52
7.52
7.51
7.26 CDCl3
1.79 H2O
N
CN
3y
1.00
1.04
1.02
1.03
9.0 8.5 8.0 7.5 7.0 6.5 6.0 5.5 5.0 4.5 4.0 3.5 3.0 2.5 2.0 1.5 1.0 0.5 0.0
f1 (ppm)

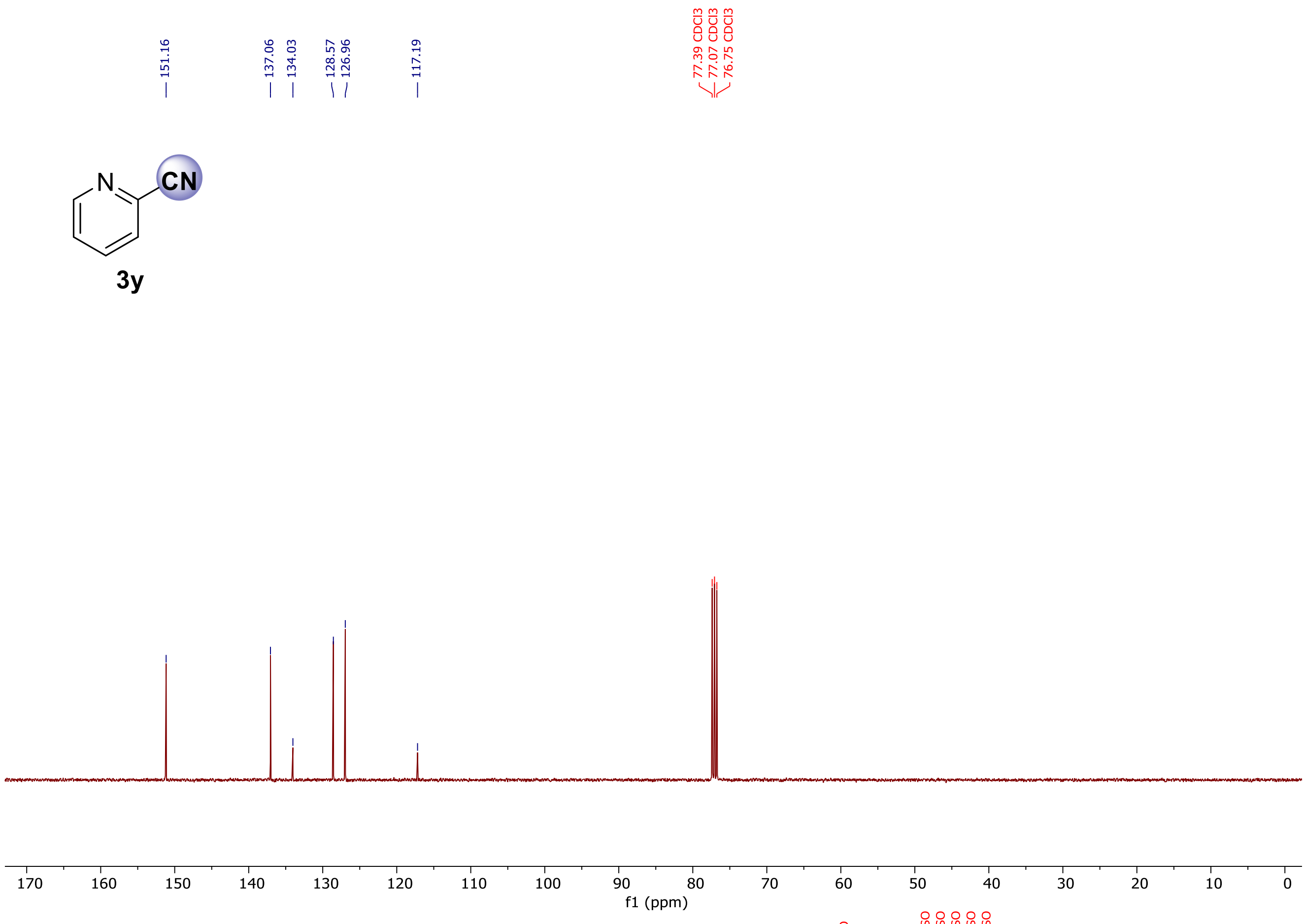

151.16
137.06
134.03
128.57
126.96
117.19
77.39 CDCl3
77.07 CDCl3
76.75 CDCl3
N
CN
3y
170 160 150 140 130 120 110 100 90 80 70 60 50 40 30 20 10 0
f1 (ppm)

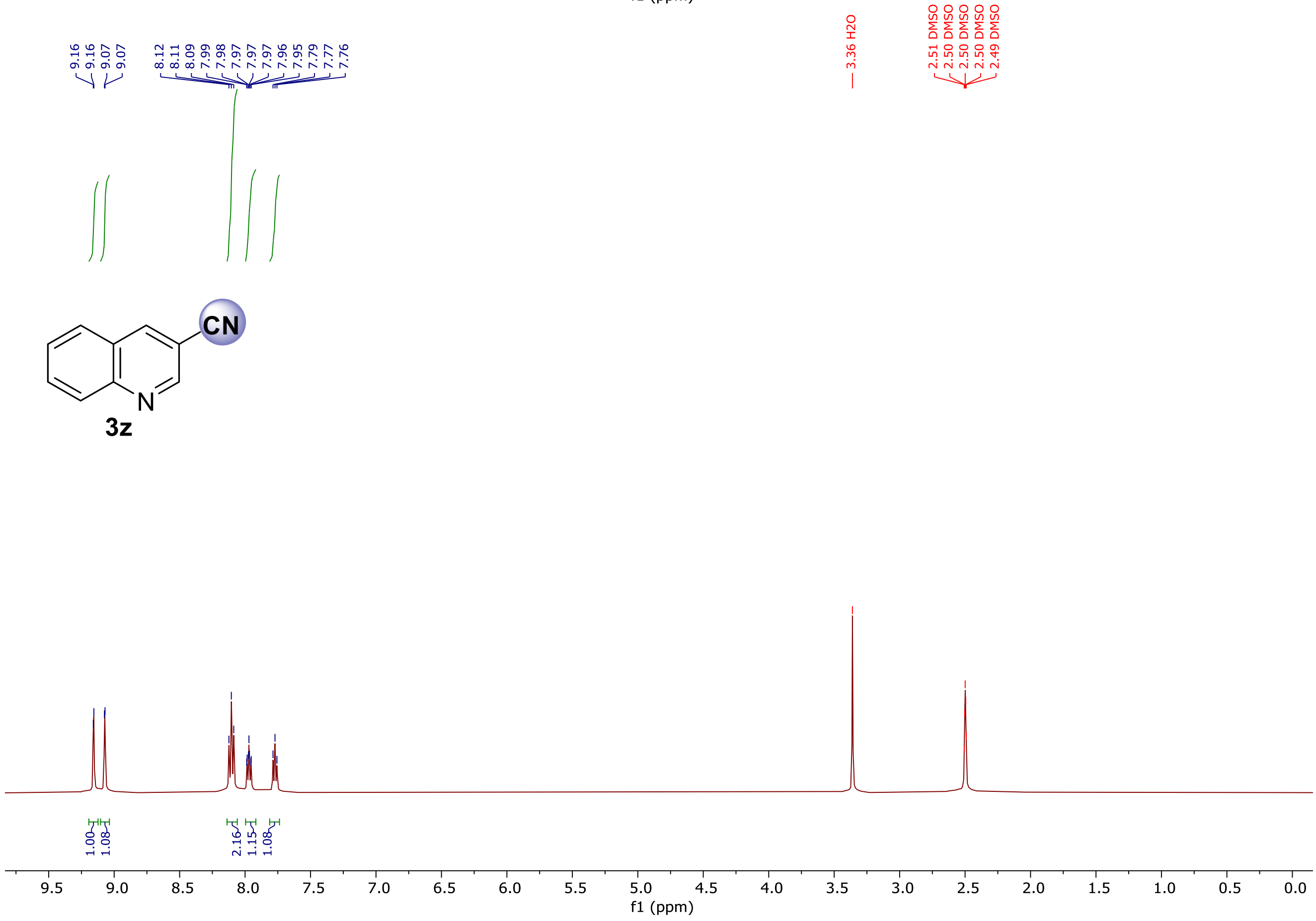

9.16
9.16
9.07
9.07
8.12
8.11
8.09
7.99
7.98
7.97
7.97
7.97
7.96
7.95
7.79
7.77
7.76
3.36 H2O
2.51 DMSO
2.50 DMSO
2.50 DMSO
2.50 DMSO
2.49 DMSO
CN
N
3z
1.00
1.08
2.16
1.15
1.08
9.5 9.0 8.5 8.0 7.5 7.0 6.5 6.0 5.5 5.0 4.5 4.0 3.5 3.0 2.5 2.0 1.5 1.0 0.5 0.0
f1 (ppm)

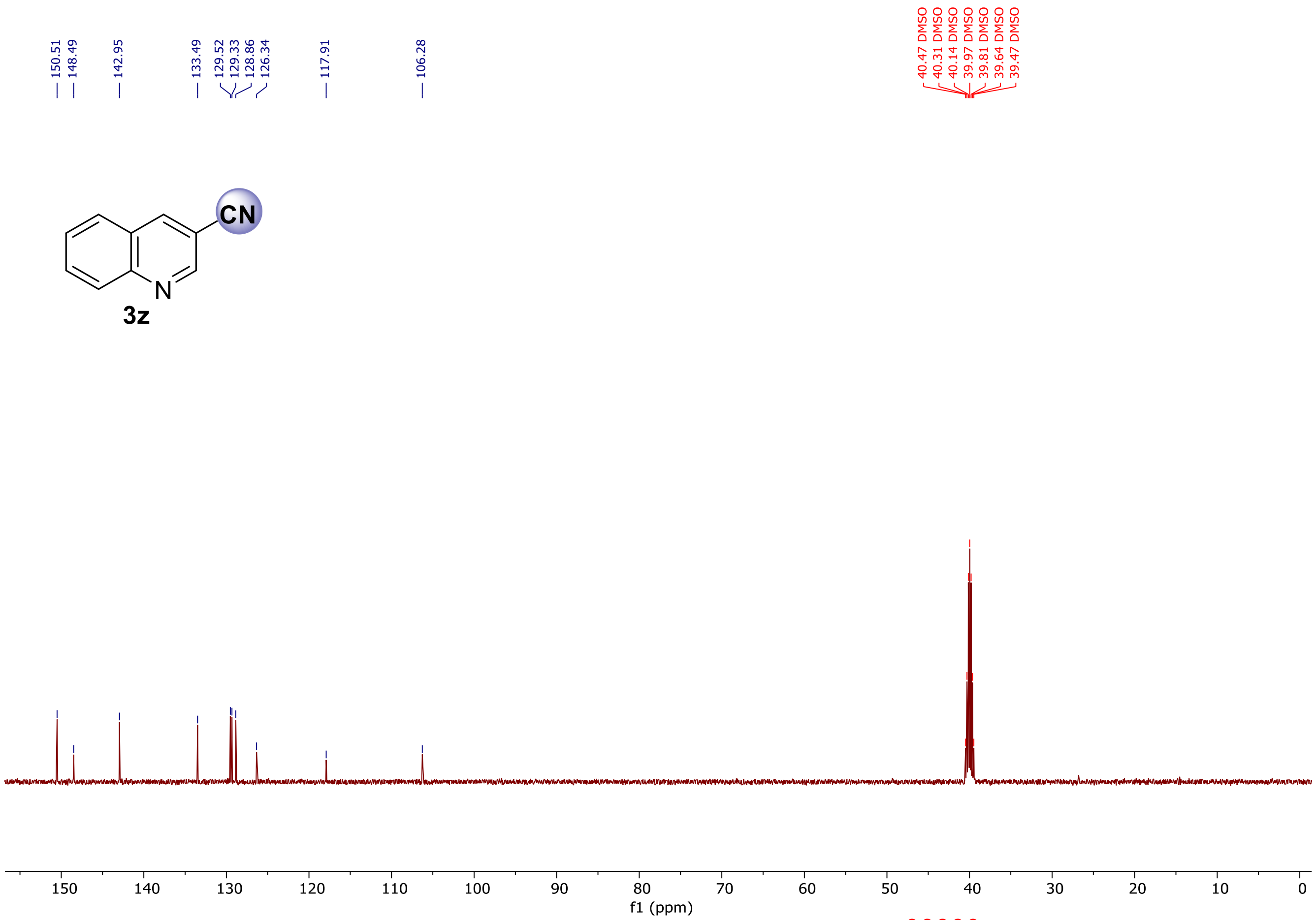
150.51
148.49
142.95
133.49
129.52
129.33
128.86
126.34
117.91
106.28
40.47 DMSO
40.31 DMSO
40.14 DMSO
39.97 DMSO
39.81 DMSO
39.64 DMSO
39.47 DMSO
CN
N
3z
f1 (ppm)

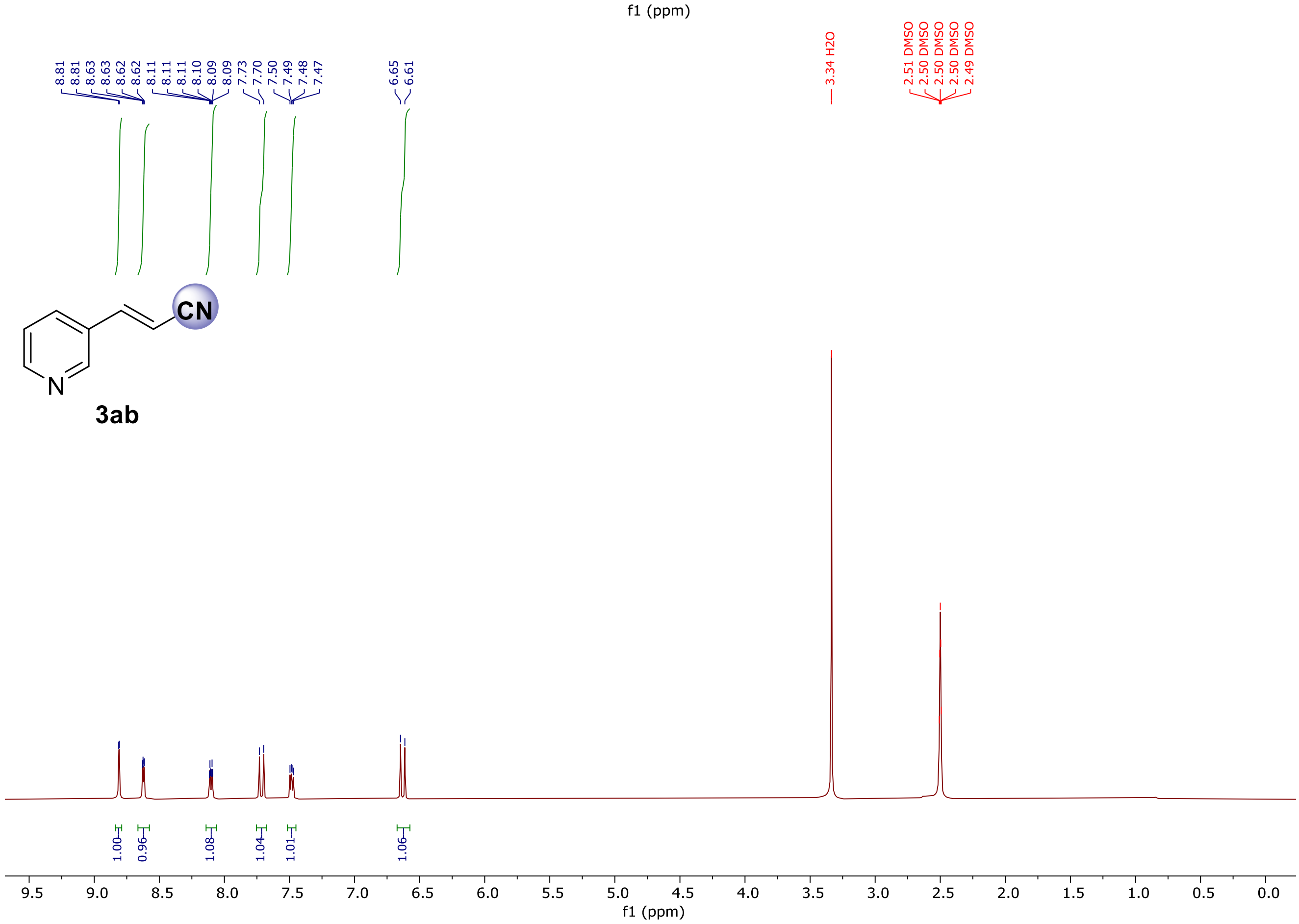
8.81
8.81
8.63
8.63
8.62
8.62
8.11
8.11
8.11
8.10
8.09
8.09
7.73
7.70
7.50
7.49
7.48
7.47
6.65
6.61
3.34 H2O
2.51 DMSO
2.50 DMSO
2.50 DMSO
2.50 DMSO
2.49 DMSO
CN
N
3ab
1.00
0.96
1.08
1.04
1.01
1.06
f1 (ppm)

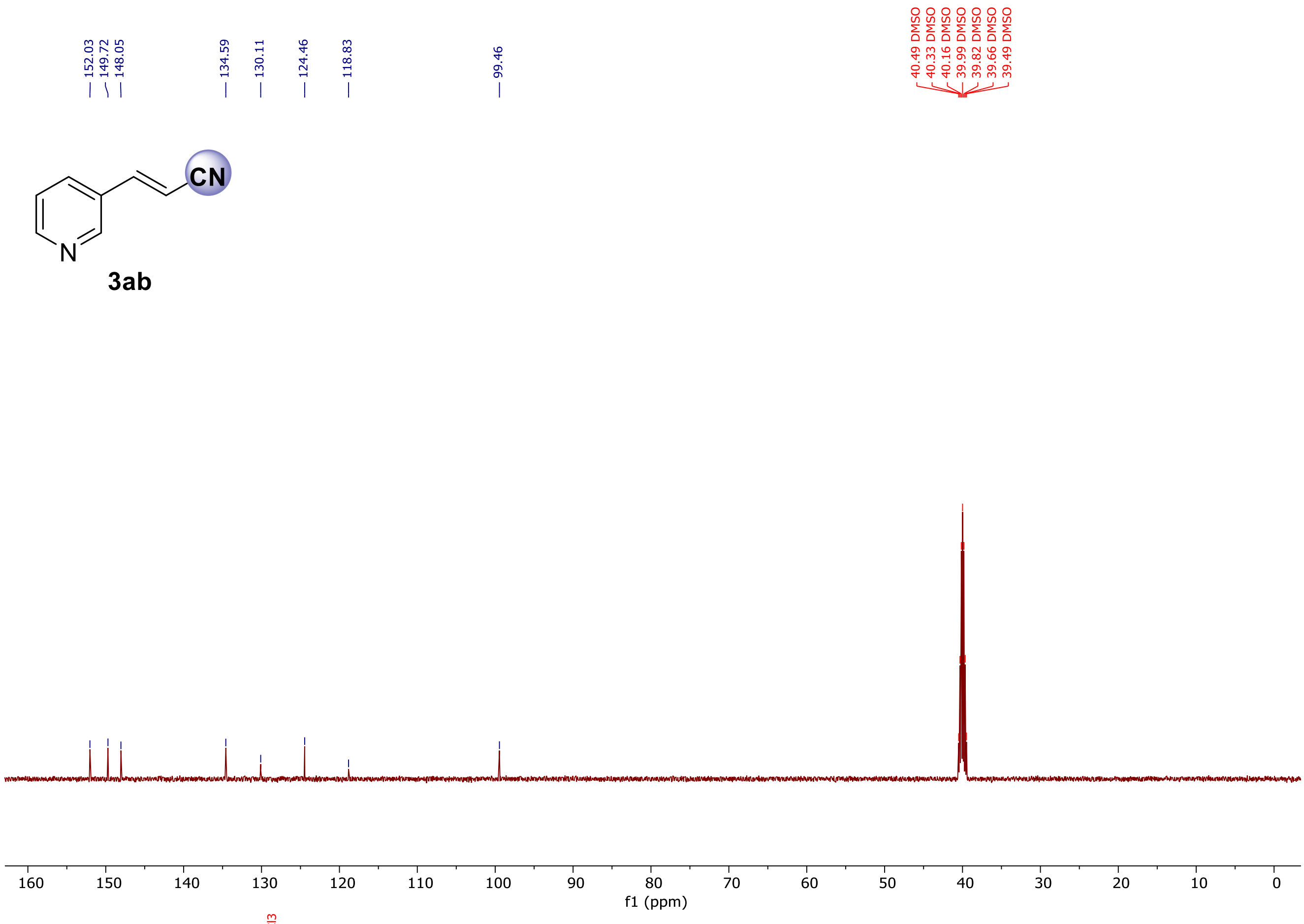
152.03
149.72
148.05
134.59
130.11
124.46
118.83
99.46
40.49 DMSO
40.33 DMSO
40.16 DMSO
39.99 DMSO
39.82 DMSO
39.66 DMSO
39.49 DMSO
CN
N
3ab
f1 (ppm)

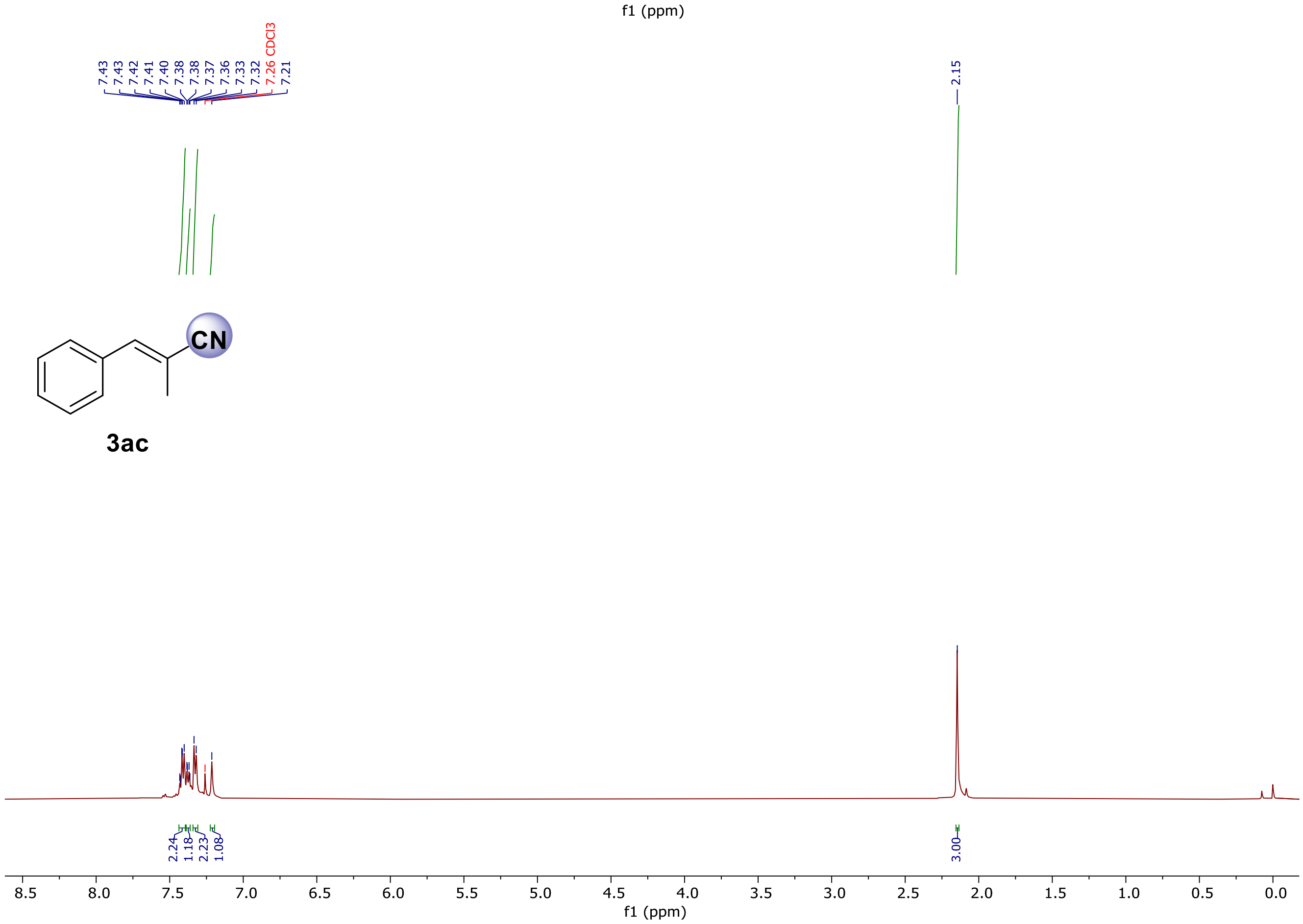
7.43
7.43
7.42
7.41
7.40
7.38
7.38
7.37
7.36
7.33
7.32
7.26 CDCl3
7.21
2.15
CN
3ac
2.24
1.18
2.23
1.08
3.00
f1 (ppm)

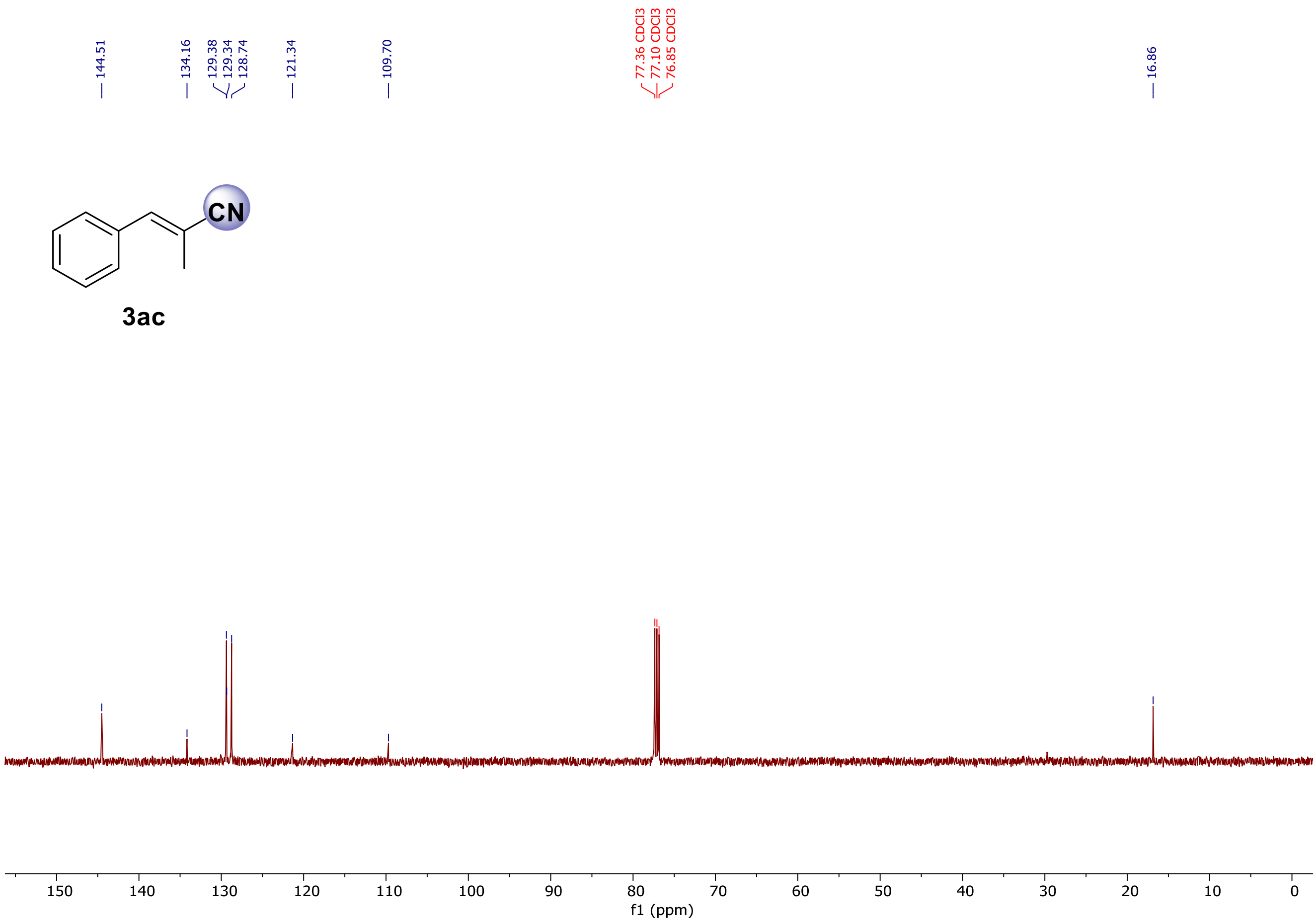

Mass spectrometry

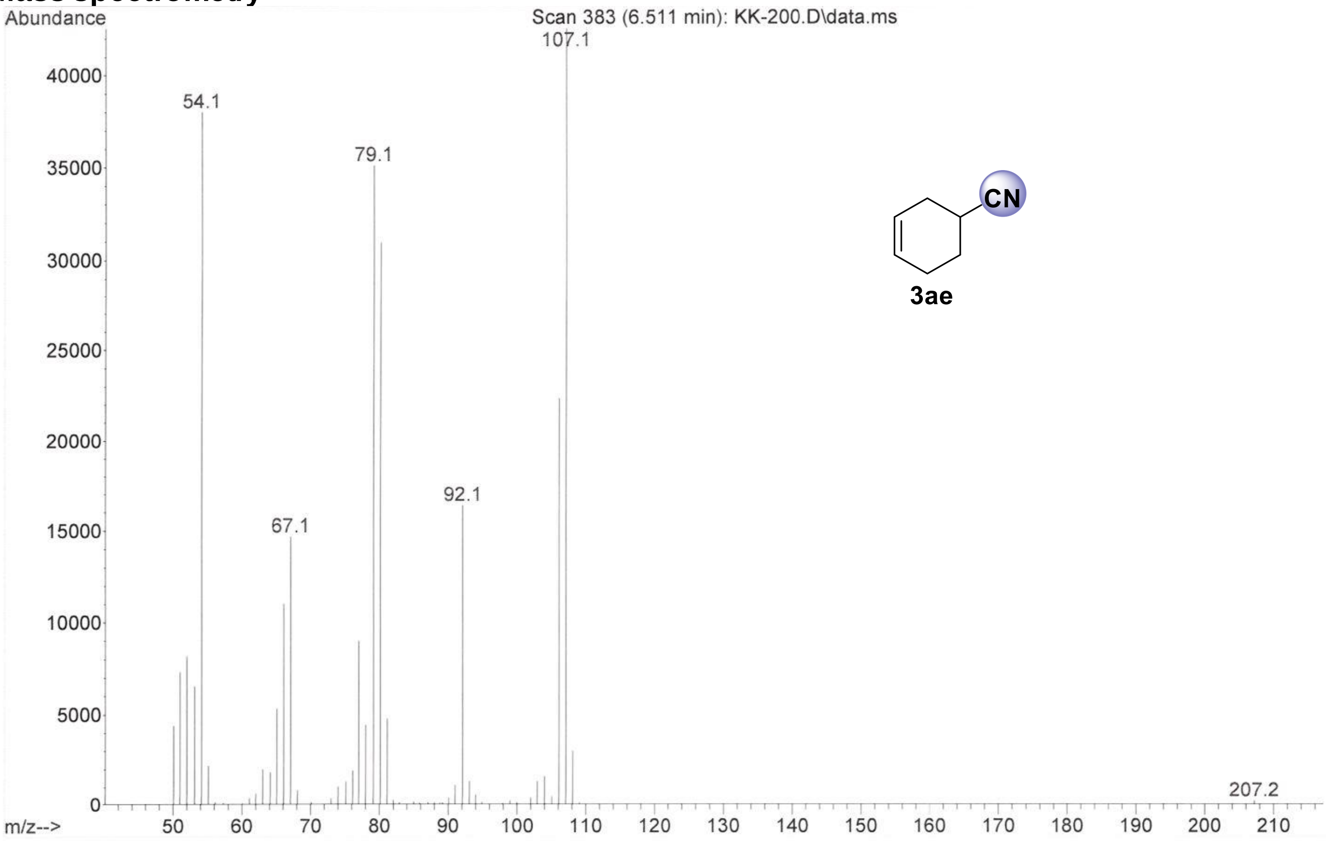

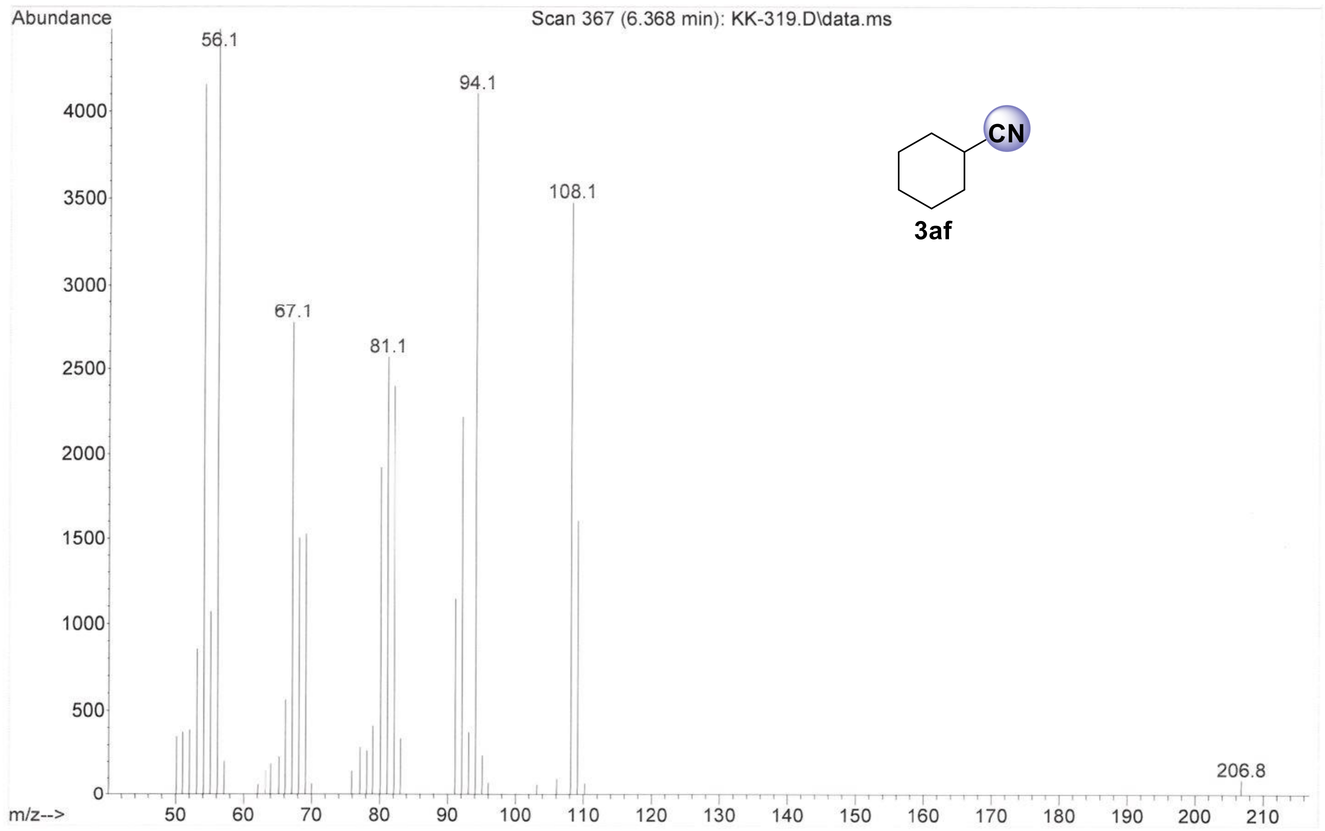
Abundance
Scan 367 (6.368 min): KK-319.D\data.ms
56.1
94.1
108.1
67.1
81.1
206.8
CN
3af
m/z-->

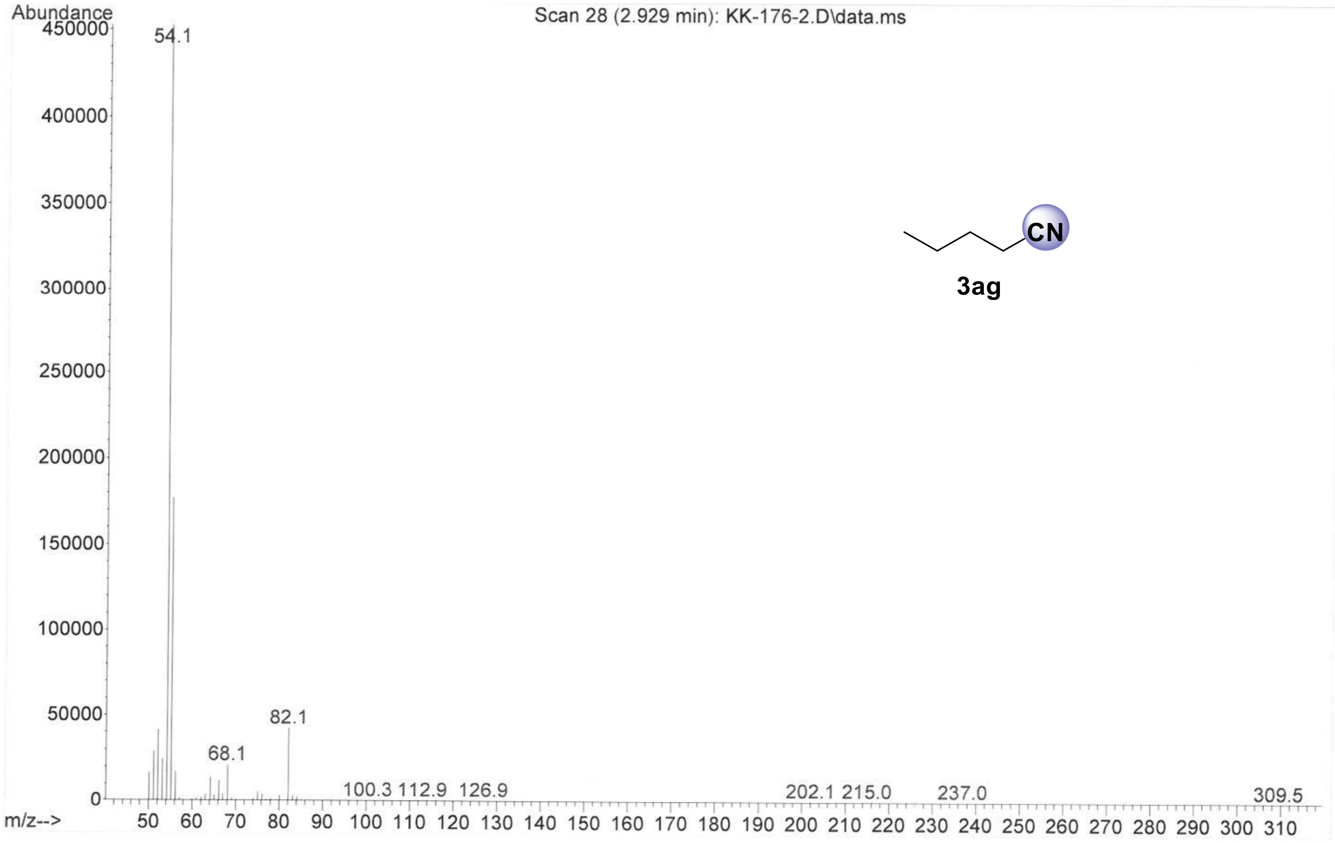
Abundance
Scan 28 (2.929 min): KK-176-2.D\data.ms
54.1
CN
3ag
82.1
68.1
100.3 112.9 126.9
202.1 215.0
237.0
309.5
m/z-->

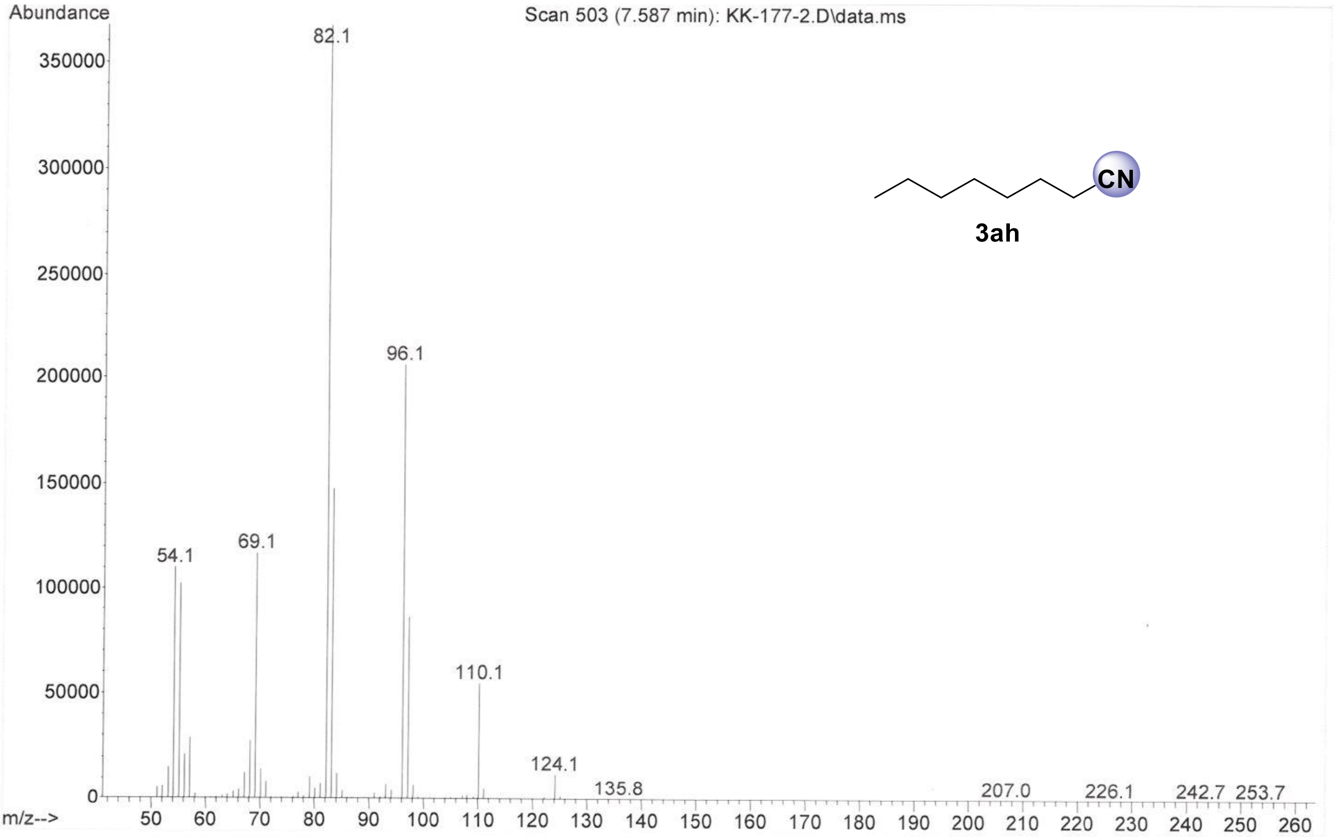
Abundance
Scan 503 (7.587 min): KK-177-2.D\data.ms
350000
300000
250000
200000
150000
100000
50000
0
m/z-->
82.1
96.1
54.1
69.1
110.1
124.1
135.8
207.0
226.1
242.7
253.7
CN
3ah

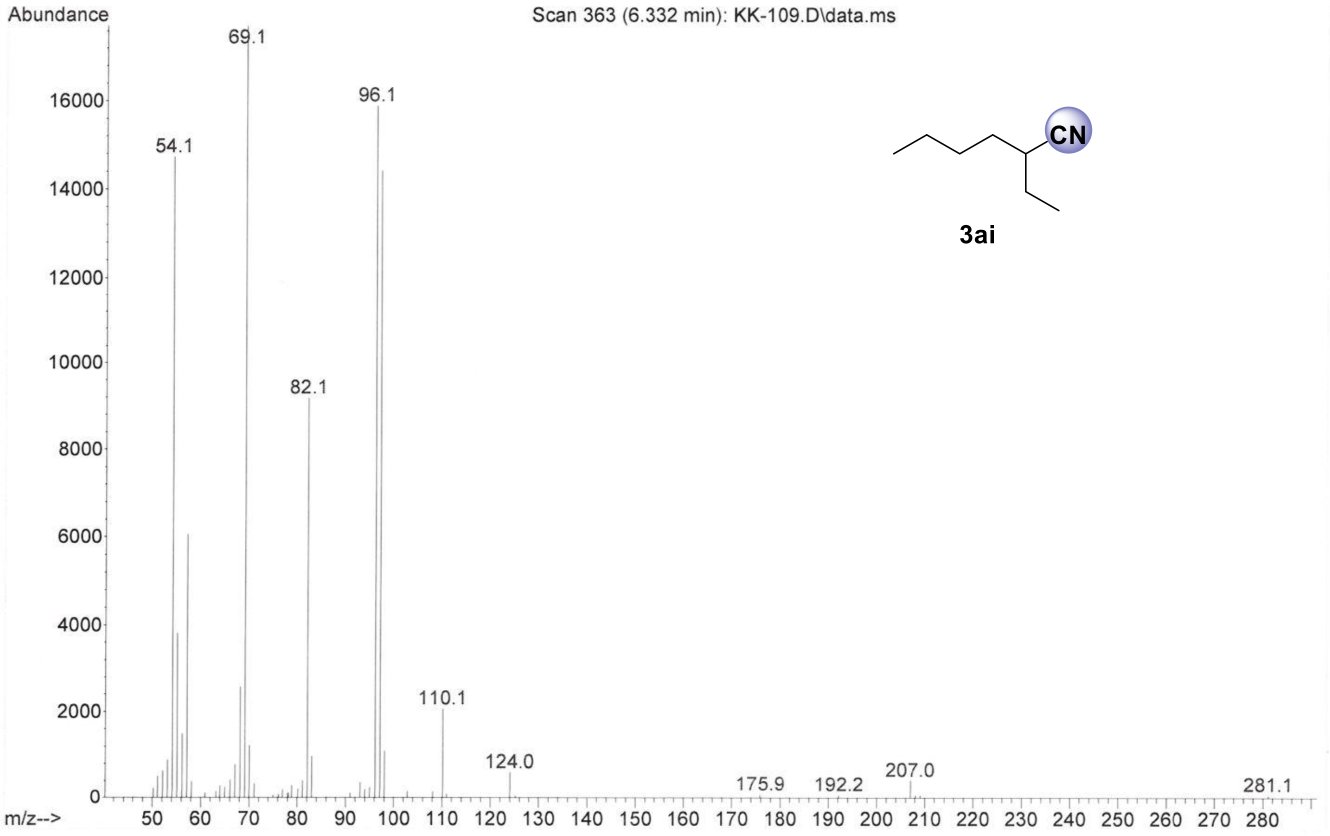
Abundance
Scan 363 (6.332 min): KK-109.D\data.ms
16000
14000
12000
10000
8000
6000
4000
2000
0
m/z-->
69.1
96.1
54.1
82.1
110.1
124.0
175.9
192.2
207.0
281.1
CN
3ai

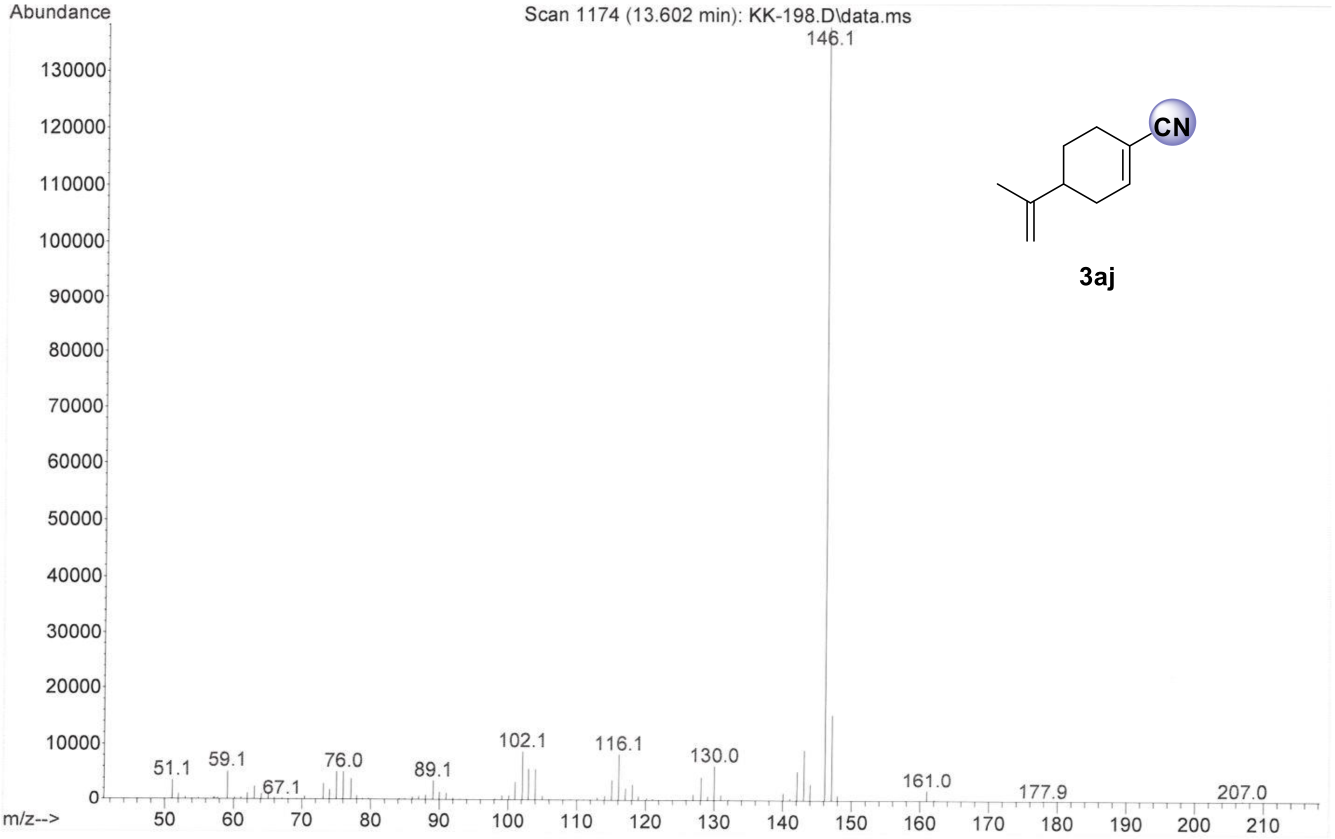
Abundance
Scan 1174 (13.602 min): KK-198.D\data.ms
146.1
130000
120000
110000
100000
90000
80000
70000
60000
50000
40000
30000
20000
10000
0
51.1
59.1
67.1
76.0
89.1
102.1
116.1
130.0
161.0
177.9
207.0
m/z-->
50 60 70 80 90 100 110 120 130 140 150 160 170 180 190 200 210
CN

3aj

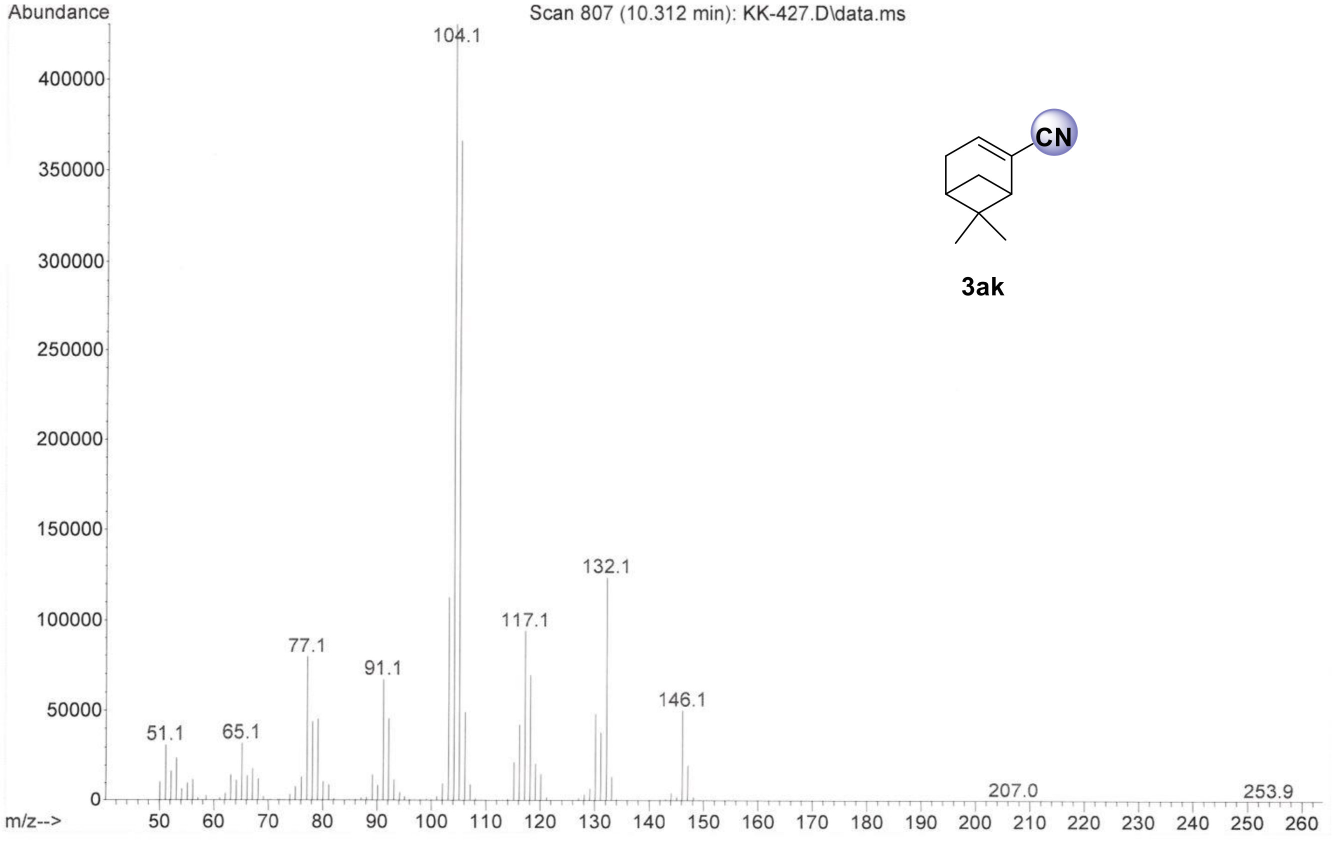
Abundance
Scan 807 (10.312 min): KK-427.D\data.ms
104.1
400000
350000
300000
250000
200000
150000
100000
50000
0
132.1
117.1
77.1
91.1
146.1
51.1
65.1
207.0
253.9
m/z-->
50 60 70 80 90 100 110 120 130 140 150 160 170 180 190 200 210 220 230 240 250 260
CN

3ak

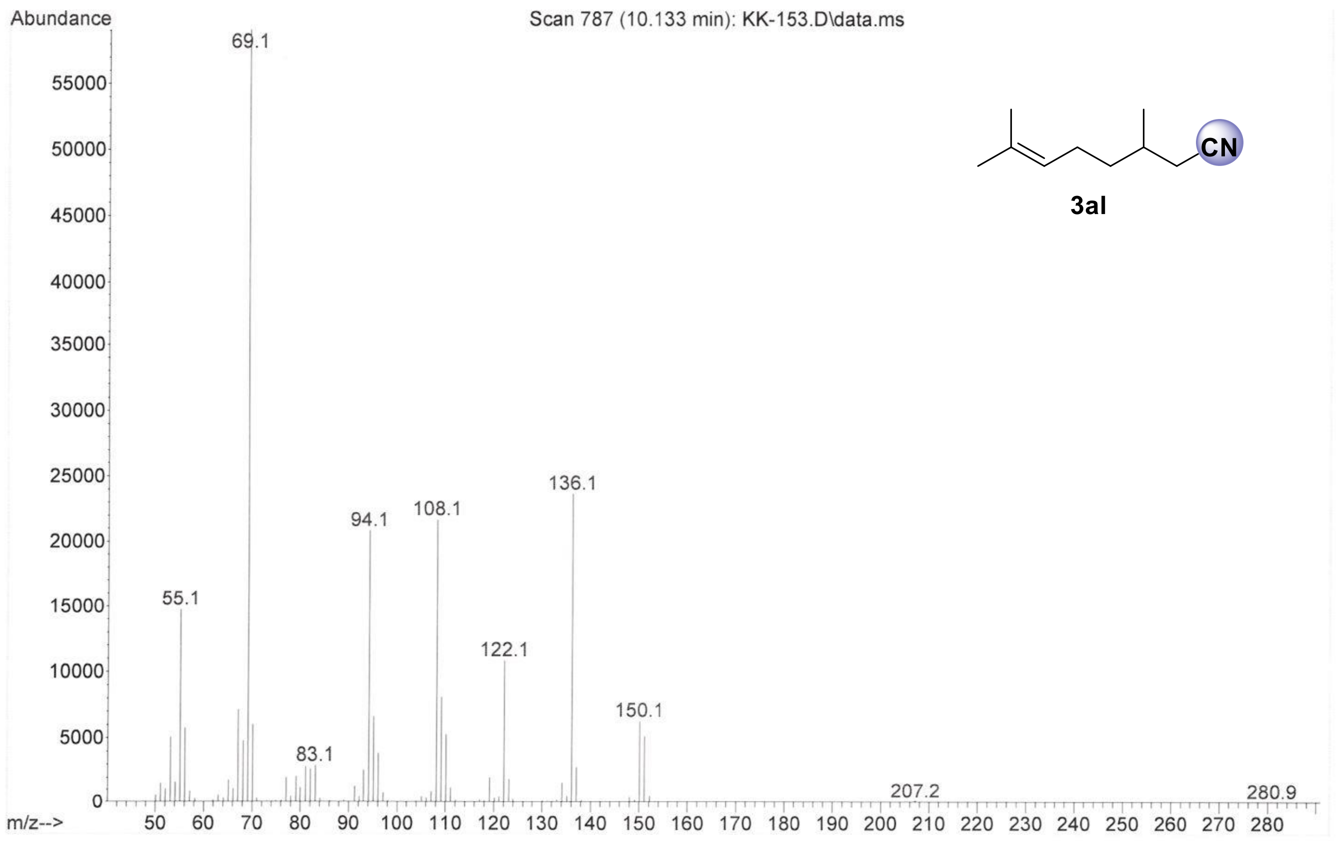

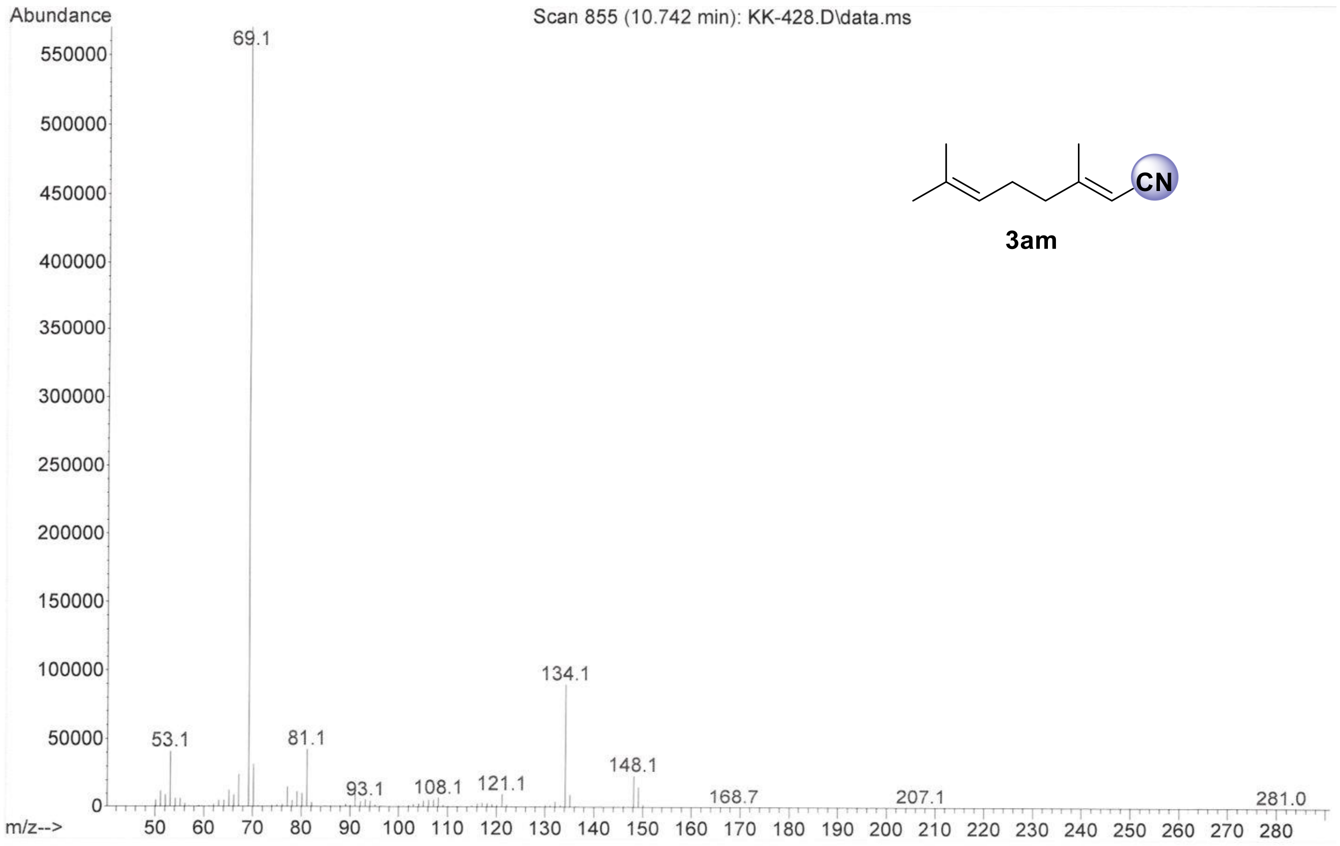

Supplementary References

1. Oishi, T., Yamaguchi, K. & Mizuno, N. Catalytic oxidative synthesis of nitriles directly from primary alcohols and ammonia. *Angew. Chem. Int. Ed.* **48**, 6286-6288 (2009).

2. Preger, Y., Root, T. W. & Stahl, S. S. Platinum-Based Heterogeneous Catalysts for Nitrile Synthesis via Aerobic Oxidative Coupling of Alcohols and Ammonia. *ACS Omega* **3**, 6091-6096 (2018).
3. Hashemi, A. N., Eshghi, H. & Lamei, K. Uniform silver nanoparticles on tunable porous N-doped carbon nanospheres for aerobic oxidative synthesis of aryl nitriles from benzylic alcohols. *Appl. Organomet. Chem.* **33**, e4835 (2019).
4. Wang, H. et al. Atomically Dispersed Ru on Manganese Oxide Catalyst Boosts Oxidative Cyanation. *ACS Catal.* **10**, 6299-6308 (2020).
5. Jagadeesh, R. V., Junge, H. & Beller, M. Green synthesis of nitriles using non-noble metal oxides-based nanocatalysts. *Nat. Commun.* **5**, 4123, (2014).
6. Shang, S. et al. High catalytic activity of mesoporous Co–N/C catalysts for aerobic oxidative synthesis of nitriles. *Catal. Sci. Technol.* **6**, 5746-5753 (2016).
7. Shang, S., Dai, W., Wang, L., Lv, Y. & Gao, S. Metal-free catalysis of nitrogen-doped nanocarbons for the ammoxidation of alcohols to nitriles. *Chem. Commun.* **53**, 1048-1051 (2017).
8. Sun, K.-k., Sun, J.-l., Lu, G.-P. & Cai, C. Enhanced catalytic activity of cobalt nanoparticles encapsulated with an N-doped porous carbon shell derived from hollow ZIF-8 for efficient synthesis of nitriles from primary alcohols in water. *Green Chem.* **21**, 4334-4340 (2019).
9. Yasukawa, T., Yang, X. & Kobayashi, S. Earth-Abundant Bimetallic Nanoparticle Catalysts for Aerobic Ammoxidation of Alcohols to Nitriles. *J. Org. Chem.* **85**, 7543-7548 (2020).
